# Supplementary material for: Unexpected assembly machinery for 4(3H)-quinazolinone scaffold synthesis
Source: Nat Commun. 2022 Oct 31;13:6522. doi: 10.1038/s41467-022-34340-3 (PMC9622831; doi:10.1038/s41467-022-34340-3)
Supplement: Supplementary file 1 — Supplementary Information [file 41467_2022_34340_MOESM1_ESM.pdf]

Supplementary Information

Unexpected assembly machinery for 4(3*H*)-quinazolinone scaffold synthesis

Xi-Wei Chen[1], Li Rao[1], Jia-Li Chen[1] and Yi Zou*[,1]

[1] College of Pharmaceutical Sciences, Southwest University, Chongqing 400715, P. R. China

* E-mail: zouyi31@swu.edu.cn

Table of contents

1. Supplementary Methods........5
1.1 Strains and culture conditions........5
1.2 Isolation of the gDNA and cDNA synthesis........5
1.3 Plasmid construction........5
1.4 The protein expression and purification in *E. coli*........7
1.5 The preparation and transformation of *A. nidulans* protoplasts........8
1.6 MALDI-TOF mass data acquisition........9
1.7 Bioinformatic analysis........10
1.8 Purification and structural characterization of compounds........11
2. Supplementary Tables........14
Supplementary Table 1. Plasmids used in this study........14
Supplementary Table 2. Products from gene (*chyC*, *chyE* and *chyH*) individual KO mutant of *P. chrysogenum*.........16
Supplementary Table 3. Analysis of *ftchy* gene cluster from *F. tricinctum*........17
Supplementary Table 4. HRMS data of compounds in this study........18
Supplementary Table 5. NMR data of compound **1** in DMSO-d_6........20
Supplementary Table 6. NMR data of compound **2** in DMSO-d_6........21
Supplementary Table 7. NMR data of compound **3** in DMSO-d_6........22
Supplementary Table 8. NMR data of compound **4** in DMSO-d_6........23
Supplementary Table 9. NMR data of compound **5** in DMSO-d_6........24
........24
Supplementary Table 10. NMR data of compound **6** in DMSO-d_6........25
Supplementary Table 11. NMR data of compound **7** in DMSO-d_6........26
Supplementary Table 12. NMR data of compound **8** in DMSO-d_6........27
Supplementary Table 13. NMR data of compound **9** in DMSO-d_6........28
Supplementary Table 14. NMR data of compound **10** in DMSO-d_6........29
Supplementary Table 15. NMR data of compound **11** in DMSO-d_6........30
Supplementary Table 16. NMR data of compound **14** in DMSO-d_6........31
3. Supplementary Figures........32
Supplementary Fig. 1 Marketed drugs with 4(3*H*)-quinazolinone scaffold........32
Supplementary Fig. 2 Representative natural 4(3*H*)-quinazolinone peptidyl alkaloids from fungi.........33
Supplementary Fig. 3 Representative natural 4(3*H*)-quinazolinone alkaloids from plants and bacteria.........34
Supplementary Fig. 4 Construction of plasmids for overexpression of *ftchy* cluster in *A. nidulans*........35
Supplementary Fig. 5 Confirmation of the on-pathway intermediates of **1** by chemical feeding experiments.........36
Supplementary Fig. 6 SDS-PAGE of proteins purified from *E. coli* BL21 in this study.........37
Supplementary Fig. 7 ftChyC-catalysed reduction or dehydrogenation reactions with different substrates........38
Supplementary Fig. 8 Schematic diagrams of ftChyA and its mutants.........39
Supplementary Fig. 9 Time-course assays of the stability of **10**........40
Supplementary Fig.10 LC-MS analyses of the *AN-ftchyA* with feeding different concentrations of Ant........41
Supplementary Fig. 11 Sequence clustering analysis of the ftChyA-C_I and ftChyA-C_T with other fungal C domains........42

Supplementary Fig. 12 Sequences alignments of ftChyA-C_1 with other identified fungal NRPS C domains........43
Supplementary Fig. 13 The genome-mined homologous *chy* clusters from different fungi. ...44
Supplementary Fig. 14 Sequence alignments of ftChyA-C_T with homologous domains from other *chy* clusters........45
Supplementary Fig. 15 The ftChyA-C_T domain is essential for catalysing the formation of **10** and **3**........47
Supplementary Fig. 16 Bioinformatic analysis of ftChyD........48
Supplementary Fig. 17 Biochemical confirmation of the amidation of ftChyD towards **3**......49
Supplementary Fig. 18 The kinetic parameters of ftChyD toward **3** and **10**........50
Supplementary Fig. 19 Sequence alignments of ftChyE with homologous enzymes and other identified glutaminase subunits of class I glutamine amidotransferases........51
Supplementary Fig. 20 LC-MS analysis of the *AN-ftchyACDEHM* in $H_2{}^{18}O$-medium showing the oxygen atom of the α-OH group of **1** could be from water........52
Supplementary Fig. 21 Conserved domain analysis of ftChyH and ftChyM........53
Supplementary Fig. 22 Sequence clustering analysis of ftChyM with other fungal α-ketoglutarate dependent dioxygenases........54
Supplementary Fig. 23 The biochemical assays of ftChyM with different substrates........55
Supplementary Fig. 24 The biochemical assays of ftChyM with **11** in ~80% $H_2{}^{18}O$-Tris-HCl buffer........56
Supplementary Fig. 25 The proposed mechanism of the (**a**) ftChyM-catalyzed C-N bond oxidative cleavage **11** to form **4**; (**b**) the ftChyM-catalyzed oxidative deamination of **2** to form **4**; (**c**) the alkaline-induced spontaneous C-2-N-3 bond closure of **4** to form **6**. 57
Supplementary Fig. 26 Time-course assays of the spontaneous conversion of **4** to **6**........58
Supplementary Fig. 27 Spontaneous cyclization of compound **2**, **5** and **4** in Tris-HCl buffer (pH 7.5)........59
Supplementary Fig. 28 Biochemical confirmation of the function of ftChyH........60
Supplementary Fig. 29 SDS-PAGE analyses of the expression of ftChyH-His, ftChyH-MBP and ftChyH-GST in *E. coli* BL21, respectively........61
Supplementary Fig. 30 Biochemical assay of ftChyM with **14** *in vitro*........62
Supplementary Fig. 31 LC-MS analyses of the *A. nidulans* with feeding **2** showing that **2** was converted to **7** by an unknown acetyltransferase of *A. nidulans*........63
Supplementary Fig. 32 HRMS spectrum (positive ionization) of compound **1**........64
Supplementary Fig. 33 HRMS spectrum (positive ionization) of compound **2**........64
Supplementary Fig. 34 HRMS spectrum (positive ionization) of compound **3**........65
Supplementary Fig. 35 HRMS spectrum (positive ionization) of compound **4**........65
Supplementary Fig. 36 HRMS spectrum (positive ionization) of compound **5**........66
Supplementary Fig. 37 HRMS spectrum (positive ionization) of compound **6**........66
Supplementary Fig. 38 HRMS spectrum (positive ionization) of compound **7**........67
Supplementary Fig. 39 HRMS spectrum (positive ionization) of compound **8**........67
Supplementary Fig. 40 HRMS spectrum (positive ionization) of compound **9**........68
Supplementary Fig. 41 HRMS spectrum (positive ionization) of compound **10**........68
Supplementary Fig. 42 HRMS spectrum (positive ionization) of compound **11**........69
Supplementary Fig. 43 HRMS spectrum (positive ionization) of compound **14**........69
Supplementary Fig. 44 HRMS spectrum (positive ionization) of compound Ant-Me-**3**. 70
Supplementary Fig. 45 HRMS spectrum (positive ionization) of compound Ant-Me-**10**. 70
Supplementary Fig. 46 ^1H NMR spectrum of compound **1** in DMSO-d_6 (400 MHz).........71
Supplementary Fig. 47 ^{13}C NMR spectrum of compound **1** in DMSO-d_6 (100 MHz)........72

Supplementary Fig. 48 ^{1}H NMR spectrum of compound **2** in DMSO-d_6 (400 MHz).............73
Supplementary Fig. 49 ^{13}C NMR spectrum of compound **2** in DMSO-d_6 (100 MHz)............74
Supplementary Fig. 50 HMBC spectrum of compound **2** in DMSO-d_6..................................75
Supplementary Fig. 51 HSQC spectrum of compound **2** in DMSO-d_6...................................76
Supplementary Fig. 52 ^{1}H-^{1}H-COSY spectrum of compound **2** in DMSO-d_6........................77
Supplementary Fig. 53 ^{1}H NMR spectrum of compound **3** in DMSO-d_6 (400 MHz).............78
Supplementary Fig. 54 ^{13}C NMR spectrum of compound **3** in DMSO-d_6 (100 MHz)............79
Supplementary Fig. 55 HMBC spectrum of compound **3** in DMSO-d_6..................................80
Supplementary Fig. 56 HSQC spectrum of compound **3** in DMSO-d_6...................................81
Supplementary Fig. 57 ^{1}H-^{1}H-COSY spectrum of compound **3** in DMSO-d_6........................82
Supplementary Fig. 58 ^{1}H NMR spectrum of compound **4** in DMSO-d_6 (400 MHz).............83
Supplementary Fig. 59 ^{13}C NMR spectrum of compound **4** in DMSO-d_6 (100 MHz)............84
Supplementary Fig. 60 ^{1}H NMR spectrum of compound **5** in DMSO-d_6 (400 MHz).............85
Supplementary Fig. 61 ^{13}C NMR spectrum of compound **5** in DMSO-d_6 (100 MHz)............86
Supplementary Fig. 62 ^{1}H NMR spectrum of compound **6** in DMSO-d_6 (400 MHz). 87
Supplementary Fig. 63 ^{13}C NMR spectrum of compound **6** in DMSO-d_6 (100 MHz)............88
Supplementary Fig. 64 ^{1}H NMR spectrum of compound **7** in DMSO-d_6 (400 MHz).............89
Supplementary Fig. 65 ^{13}C NMR spectrum of compound **7** in DMSO-d_6 (100 MHz)............90
Supplementary Fig. 66 ^{1}H NMR spectrum of compound **8** in DMSO-d_6 (400 MHz).............91
Supplementary Fig. 67 ^{13}C NMR spectrum of compound **8** in DMSO-d_6 (100 MHz)............92
Supplementary Fig. 68 ^{1}H NMR spectrum of compound **9** in DMSO-d_6 (400 MHz).............93
Supplementary Fig. 69 ^{13}C NMR spectrum of compound **9** in DMSO-d_6 (100 MHz)............94
Supplementary Fig. 70 ^{1}H NMR spectrum of compound **10** in DMSO-d_6 (400 MHz)...........95
Supplementary Fig. 71 ^{13}C NMR spectrum of compound **10** in DMSO-d_6 (100 MHz)...........96
Supplementary Fig. 72 HMBC spectrum of compound **10** in DMSO-d_6................................97
Supplementary Fig. 73 HSQC spectrum of compound **10** in DMSO-d_6.................................98
Supplementary Fig. 74 ^{1}H-^{1}H COSY spectrum of compound **10** in DMSO-d_6 99
Supplementary Fig. 75 DEPT-135° spectrum of compound **10** in DMSO-d_6.......................100
Supplementary Fig. 76 ^{1}H NMR spectrum of compound **11** in DMSO-d_6 (400 MHz).........101
Supplementary Fig. 77 ^{13}C NMR spectrum of compound **11** in DMSO-d_6 (100 MHz).........102
Supplementary Fig. 78 HMBC spectrum of compound **11** in DMSO-d_6..............................103
Supplementary Fig. 79 HSQC spectrum of compound **11** in DMSO-d_6...............................104
Supplementary Fig. 80 ^{1}H-^{1}H COSY spectrum of compound **11** in DMSO-d_6 105
Supplementary Fig. 81 DEPT-135° spectrum of compound **11** in DMSO-d_6.......................106
Supplementary Fig. 82 ^{1}H NMR spectrum of compound **14** in DMSO-d_6 (400 MHz).........107
Supplementary Fig. 83 ^{13}C NMR spectrum of compound **14** in DMSO-d_6 (100 MHz).........108
Supplementary Fig. 84 An efficient self-circulation system among ftChyA, ftChyD and ftChyM-catalysed reactions..109
4. Supplementary References..110

1. Supplementary Methods

1.1 Strains and culture conditions

Fusarium tricinctum CGMCC 3.4731 was cultured at 25 °C, 7 days on PDA medium (26 g/L Potato Dextrose Water, 2% agar) for sporulation or culture at 25 °C on PDB medium for 4 days to extract genome DNA (gDNA). *A. nidulans* L08030 was culture at 37 °C for 3-4 days on solid CD medium (10 g/L glucose, 50 mL/L 20 × nitrate salts, 1 mL/L trace elements, 20 g/L agar) containing 10 mM uridine, 5 mM uracil, 1 μg/mL pyridoxine HCl and 0.25 μg/mL riboflavin for sporulation. The *A. nidulans* mutants was culture at 25 °C for 3.5 days on solid CD-ST medium (20 g/L starch, 10 g/L casein hydrolysate (acid), 50 mL/L nitrate salts, 1 mL/L trace elements, 20 g/L Agar) for heterologous expression and compounds production.

1.2 Isolation of the gDNA and cDNA synthesis

Fusarium tricinctum was cultured in PDB medium (26 g/L Potato Dextrose Water) at 25 °C and 220 rpm for 4 days. The mycelium was collected and used for gDNA extraction by Cetyltrimethylammonium Bromide (CTAB) method (20 g/L CTAB, 81.8 g/L NaCl, 186.1 g /L $Na_2EDTA \cdot 2H_2O$ and 0.1 M Tris-HCl pH 8.0). The total RNA of the *A. nidulans::ftchy* was isolated from the strain grown in liquid CD-ST production medium (20 g/L starch, 20 g/L casein hydrolysate, 50 mL/L nitrate salts and 1 mL/L trace elements) at 25 °C and 220 rpm for 3 days. RNA was extracted by TRLZOL® Reagent (Ambion), followed by cDNA reverse transcription with the Transcriptor First Strand cDNA Synthesis Kit (Roche).

1.3 Plasmid construction

The sequence of *ftchy* gene cluster is deposited in GenBank under accession number OP651004. The gDNA sequences of *ftchy* gene, the protein sequences of ftChy protein, the primer sequences are listed in the Source Data file. The plasmids are summarized in **Supplementary Table 1**.

To construct the expression plasmids of *ftchyA~H* genes for *A. nidulans*, each gene with its terminator (~ 400 bp) was amplified from the gDNA of *F. tricinctum* CGMCC 3.4731 by PCR using primer pairs pANR-*ftchyA*-F1/R1, pANR-*ftchyA*-F2/R2, pANR-*ftchyA*-F3/R3, pANU-*ftchyC*-F/R, pANU-*ftchyD*-F/R, pANU-*ftchyE*-F/R, pANP-*ftchyM*-F/R, pANP-*ftchyH*-F/R, pANU-*ftchyC*-F/R2, pANU-*ftchyH*-F/R2, pANP-*ftchyM*-F2/R yielding plasmids pIM 3201-3206, through yeast homologous recombination in *S. cerevisiae* BJ5464-NpgA. The *glaA*, *gpdA* and *amyB* promoters were amplified from vectors pANU, pANR and pANP by using primer pairs *glaA*-F/*glaA*-R, *gpdA*-F/*gpdA*-R and *amyB*-F/*amyB*-R, respectively. Gene *ftchyA* was cloned into vector pANR with *gpdA* promoter yielding plasmid pIM 3201. Genes *ftchyC, ftchyD* and *ftchyE* were cloned into vector pANU yielding plasmid pIM 3202, while the promoters *gpdA*, *glaA* and *amyB* were used for *ftchyC, ftchyD* and *ftchyE*, respectively.

Genes *ftchyM* and *ftchyH* were cloned into vector pANP yielding plasmid pIM 3203, while the promoters *gpdA* and *amyB* were used for *ftchyM* and *ftchyH*, respectively. Genes *ftchyC* and *ftchyD* were cloned into vector pANU yielding plasmid pIM 3204, while the promoters *gpdA* and *glaA* were used for *ftchyC* and *ftchyD*, respectively. Genes *ftchyH* and *ftchyM* were cloned into vector pANP with the promoter *amyB* yielding plasmid pIM 3205 and pIM 3206, respectively.

To express *ftchyA* in *E. coli* BL21, intron-free *ftchyA* was cloned from the cDNA of *A. nidulans* containing *ftchy* cluster using the primer pairs pYEU-*ftchyA*-F1/R1, pYEU-*ftchyA*-F2/pANR-*ftchyA*-R1, pANR-*ftchyA*-F2/R2, pANR-*ftchyA*-F3/pYEU-*ftchyA*-R2. The corresponding overlapping fragments were ligated to vector pYEU by yeast homologous recombination to obtain plasmid pIM 3207. The plasmid pIM 3207 was further digested with *BamH* I and *EcoR* I to obtain *ftchyA* and subsequently inserted into the *BamH* I and *EcoR* I digested pColdI (an expression vector with His tag on N-terminus) to yield plasmid pIM 3208. The mutated expression plasmids pIM 3209-3211 were constructed through yeast homologous recombination in *S. cerevisiae* BJ5464-NpgA. The mutated fragments were amplified by PCR using primer pairs pYEU-*ftchyA*-F1/pANR-*ftchyA*-R1, pANR-*ftchyA*-F2/pYEU-*ftchyA*-C_1-$H_{987}A$-R, pYEU-*ftchyA*-C_1-$H_{987}A$-F/pANR-*ftchyA*-R2, pANR-*ftchyA*-F3/pYEU-*ftchyA*-R2, pANR-*ftchyA*-F2/pYEU-*ftchyA*-C_1^*-R, pYEU-*ftchyA*-C_1^*-F/pANR-*ftchyA*-R2, pANR-*ftchyA*-F3/pYEU-*ftchyA*-C_T-$H_{2075}A$-R, pYEU-*ftchyA*-C_T-$H_{2075}A$-F/pYEU-*ftchyA*-R2. The plasmids pIM 3209-3211 were digested and ligated in to pColdI by same method to generate plasmids pIM 3212-3214, respectively. To express the stand-alone ftChyA-A_1, ftChyA-A_2 and ftChyA-C_T domain in *E. coli* BL21, the plasmids pIM 3215-3217 were constructed, respectively. The intron-free *ftchyA*-A_1 was amplified by PCR using primer pairs pColdI-*ftchyA*-A_1-F/R. The products were digested with *Kpn* I and *EcoR* I, and subsequently inserted into the *Kpn* I and *EcoR* I digested pColdI to create plasmid pIM 3215. The intron-free *ftchyA*-A_2 was amplified by PCR using primer pairs pQ8-*ftchyA*-A_2-F/R. The products were digested with *EcoR* I and *Not* I, and subsequently inserted into the *EcoR* I and *Not* I digested pQ8 (an expression vector with MBP tag on N-terminus) to yield plasmid pIM 3216. The intron-free *ftchyA*-C_T was amplified by PCR using primer pairs pColdI-*ftchyA*-C_T-F/R. The products were digested with *BamH* I and *EcoR* I, and then cloned into pColdI to yield plasmid pIM 3217. The PCR products were amplified by PCR using primer pairs pColdI-*ftchyA*ΔC_T-F/R and digested with *Spe* I and *EcoR* I. The products were cloned into the *Spe* I and *EcoR* I digested pIM 3208 to generate plasmid pIM 3218. The plasmid pIM 3218 was used to purify the C_T domain-truncated ftChyAΔC_T (A_1-T_1-C_1-A_2-T_2) from *E. coli* BL21. To express the ftChyA-A_1T_1 in *E. coli* BL21, the intron-free *ftchyA*-A_1T_1 was amplified by PCR using primer pairs pColdI-*ftchyA*-A_1-F/pColdI-*ftchyA*-A_1T_1-R. The products were digested with *Kpn* I and *EcoR* I, and then cloned into pColdI to yield plasmid pIM 3219.

To express *NpgA* without N-His tag in *E. coli* BL21, intron-free *NpgA* was cloned from the cDNA of *A. nidulans* by using the primer pairs pET28a-*NpgA*-F/R. The PCR products were digested with *Hind* III and *Nco* I and subsequently inserted into the *Hind*

III and *Nco* I digested pET28a to yield plasmid pIM 3220. To express *NpgA* with N-His tag, intron-free *NpgA* was cloned using the primer pairs pColdI-*NpgA*-F/R. The PCR products were digested with *Sac* I and *Hind* III and subsequently inserted into pColdI to yield plasmid pIM 3221.The plasmid pIM 3222 used for protein expression *ftchyC* in *E. coli* BL21 was constructed by amplifying open reading frames from cDNA reverse transcribed from *A. nidulans* containing *ftchy* cluster using primer pairs pColdI-*ftchyC*-F/R. The PCR products were digested with *Kpn* I and *EcoR* I and subsequently inserted into the *Kpn* I and *EcoR* I digested pColdI to yield plasmid pIM 3222. Intron-free *ftchyD* was cloned from the cDNA of *A. nidulans* containing *ftchy* cluster using the primer pairs pET-Duet-*ftchyD*-F/R. The PCR products were digested with *BamH* I and *Not* I, and subsequently inserted into the *BamH* I and *Not* I digested pET-Duet (an expression vector with His tag on *N*-terminus) to yield plasmid pIM 3223. Intron-free *ftchyE* was amplified by PCR using primer pairs pQ8-*ftchyE*-F/R. The PCR products were digested with *EcoR* I and *Not* I and subsequently inserted into the *EcoR* I and *Not* I digested pQ8 to yield plasmid pIM 3224. To express *ftchyM* in *E. coli* BL21, intron-free *ftchyM* was cloned using the primer pairs pColdI-*ftchyM*-F/R. The PCR products were digested with *Kpn* I and *BamH* I and subsequently inserted into the *Kpn* I and *BamH* I digested pColdI to yield plasmid pIM 3225. The intron-free *ftchyH* was amplified by PCR using primer pairs pColdI-*ftchyH*-F/R. The fragments were digested with *Kpn* I and *BamH* I and subsequently inserted into the *Kpn* I and *BamH* I digested pColdI to yield plasmid pIM 3226. The *ftchyH* was also cloned into the *EcoR* I and *Not* I digested pQ8 and pGEX-4t-1 (an expression vector with Glutathione S-transferase tag on N-terminus) to create plasmids pIM 3227 and pIM 3228, respectively.

The plasmid used for protein expression of *ftchyH* in *S. cerevisiae* BJ5464-NpgA was constructed by amplifying open reading frames from *A. nidulans* containing *ftchy* cluster using primer pairs pYEU-*ftchyH*-F/pYEU-*ftchyH*-R. The fragments were ligated to vector pYEU (an expression vector with Flag tag on N-terminus and His tag on C-terminus) with the promoter ADH_2P and the terminator ADH_2T by yeast homologous recombination to obtain plasmid pIM 3229.

All the plasmids were confirmed by DNA sequencing by Sangon Biotech (Shanghai) Co., Ltd.

1.4 The protein expression and purification in *E. coli*

Recombinant plasmids pIM 3208, pIM 3212-3218 and pIM 3221-3228 were transformed into *E. coli* BL21 (DE3) strain by heat shock transformation, respectively. The mono colony was cultivated in liquid LB medium (25 g/L LB broth) with 100 μg/mL ampicillin (for ftChyA and its mutants, NpgA, ftChyC, ftChyH-N-His, ftChyH-N-GST, ftChyA-A_1, ftChyA-C_T, ftChyM, ftChyD) or 50 μg/mL of kanamycin (for ftChyH-N-MBP, ftChyE, ftChyA-A_2) at 37 °C overnight. The bacterial solution was then transferred in liquid LB medium (1 L) containing 100 μg/mL ampicillin or 50 μg/mL of kanamycin at 37 °C and 220 rpm to OD_{600} of 0.4-0.6. And then, the cultures were maintained at 16 °C for 30 min. Protein expression was induced at 16 °C and 220

rpm for 20 h after adding 0.2 mM isopropylthio-β-D-galactoside (IPTG). Cells were collected by centrifugation at 4 °C, 3000 g for 8 min.

The cells of ftChyA were resuspended in 20 mL buffer A (50 mM Tris-HCl, 500 mM NaCl, 10% glycerol, pH 7.5) and were lysed by sonication on ice. Cellular debris were removed by centrifugation at 4 °C, 23000 g for 40 min. The protein was purified by nickel nitrilotriacetic acid (Ni-NTA) agarose resin. And then, the protein was eluted by buffer A containing 250 mM imidazole. The purified protein was passed through a PD-10 desalting column (GE Healthcare) and eluted with buffer C (50 mM Tris-HCl, 50 mM NaCl, 5% glycerol, pH 7.5). The protein was concentrated using ultrafiltration centrifugal tube (Millipore Amicon ® Ultra-15 mL). Finally, the protein was flash frozen in liquid nitrogen and saved at -80 °C. ftChyA mutants, NpgA, ftChyC, ftChyA-A_1, ftChyA-A_1T_1, ftChyA-C_T, ftChyAΔC_T and ftChyD were treated in the same way. ftChyM was also treated in the same way, but it was resuspended in buffer A containing 1 mM DTT (DL-Dithiothreitol) and eluted buffer containing 250 mM imidazole and 1mM DTT.

ftChyA-A_2 and ftChyE were expressed with N-MBP tag, respectively. The cells of ftChyA-A_2 or ftChyE were resuspended in 20 mL binding buffer (20 mM Tris-HCl, 200 mM NaCl, 1 mM EDTA, 1 mM DTT, pH 7.5) and were lysed by sonication on ice. Cellular debris were removed by centrifugation at 4 °C, 23000 g for 40 min. The protein was purified using dextrin sepharose resin. The protein-bound resin was washed with elution buffer (10 mM maltose in binding buffer). The pooled fraction was concentrated and was exchanged into buffer C.

The purified enzyme was analyzed by SDS-PAGE, and the concentration was measured by BCA protein quantification kit (Beijing Dingguo Changsheng Biotechnology Co., Ltd).

1.5 The preparation and transformation of *A. nidulans* protoplasts

A. nidulans was cultured in solid CD medium containing 10 mM uridine, 5 mM uracil, 1 μg/mL pyridoxine HCl and 0.25 μg/mL riboflavin at 37 °C for 4 days, and then spores were collected in 20% glycerol. The spores were inoculated in 40 mL liquid CD medium and cultured at 37 °C and 220 rpm for 9 h. After the germination of spores, cultures were centrifuged at 4 °C, 2500 g for 8 min to harvest the mycelia. The precipitation was washed two times with 15 mL osmotic buffer (1.2 M $MgSO_4 \cdot 7H_2O$, 10 mM sodium phosphate, pH 5.8) at 4 °C, 2500 g for 8 min. Then the precipitation was resuspended with 10 mL osmotic buffer containing 30 mg Lysing Enzymes (Sigma) and 20 mg Yatalase (Takara). The suspension was transferred into 50 mL Erlenmeyer flask, and cultured at 28 °C, 80 rpm for 14 h. The culture fluid was poured directly in a sterile 50 mL centrifugal tube and overlaid gently with 10 mL of trapping buffer (0.6 M sorbitol, 0.1 M Tris-HCl, pH 7.0), then centrifuged at 4 °C, 3000 g for 20 min. The protoplasm layer was transferred and fully scattered into 2 × STC buffer (1.2 M sorbitol, 10 mM $CaCl_2$, 10 mM Tris-HCl, pH 7.5), and centrifuged at 4 °C, 3000 g for 8 min. The supernatant was removed and STC buffer was added to resuspend the protoplasts

for transformation.

1.6 MALDI-TOF mass data acquisition

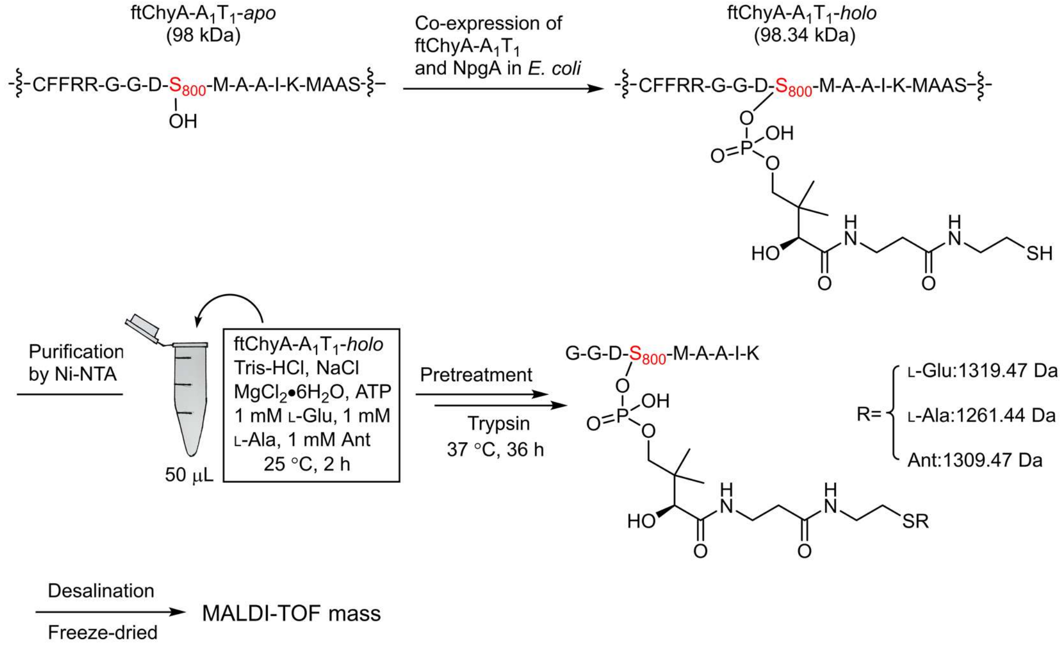

To gain *holo* form of ftChyA-A_1T_1, the recombinant plasmids pIM 3219 and pIM 3220 were co-transformed into *E. coli* BL21 (DE3) strain. The mono colony with *ftchyA-A_1T_1* and *NpgA* was selected in solid LB medium with 100 μg/mL ampicillin and 50 μg/mL of kanamycin at 37 ℃ overnight. Protein induction and co-expression of ftChyA-A_1T_1 and NpgA as described above. The protein of ftChyA-A_1T_1-*holo* was purified by Ni-NTA agarose resin. The 50 μL reaction mixture containing 40 μM ftChyA-A_1T_1-*holo*, 20 mM Tris-HCl (pH 8.5), 100 mM NaCl, 10 mM $MgCl_2 \cdot 6H_2O$, 4 mM ATP, 1 mM L-Glu, 1 mM L-Ala and 1 mM Ant for 2 hours at 25 °C.

To analysis of ftChyA-A_1 recognized and loaded amino acid to the Ser binding site (S_{800}) of ftChyA-T_1 by MALDI-TOF mass, the following steps will be performed. The 50 μL reaction mixture and 150 μL urea solution (8 M) were added to a 10 kDa ultrafiltration centrifugal tube, and then centrifuged at room temperature (RT), 12,000 g for 20 min. 200 μL urea solution (8 M) was added and centrifuged, which process will be repeated twice. 4 μL DTT (1 M) and 150 μL urea solution (8 M) were added and the nozzle of tube was sealed with parafilm, which incubated at 37 °C for 2 h. Next, 15 μL iodoacetamide solution (1 M) was added and incubated at RT for 1 h in the dark, subsequently centrifuged at RT, 12,000 g for 20 min. 200 μL urea solution (8 M) was added and centrifuged, which process will be repeated twice. 200 μL NH_4HCO_3 solution (50 mM) was added and centrifuged, which process will be repeated three times. The outer tube of the ultrafiltration centrifugal tube was replaced with a new outer tube. 200 μL trypsin solution (10 μg/mL) was added and the nozzle of tube was sealed with parafilm, which incubated at 37 °C for 36 h to decompose the protease into peptide fragments. The parafilm was removed and centrifuged. 40 μL NH_4HCO_3 solution (50 mM) was added and centrifuged. The lyophilized peptide sample was

freeze-dried at 4°C. The sample was reconstituted with 200 μL 0.1% TFA (trifluoroacetic acid) and centrifuged with a transient. A Zip Tip C18 micro desalting column was used for desalting peptide fragments. Elution buffer (90% acetonitrile (ACN) with 0.1% TFA) was used to elute the peptide, which was freeze-dried at 4 °C. MALDI-TOF mass data was recorded by a Bruker MALDITOF mass spectrometer Autoflex Speed and α-cyano-4-hydroxycinnamic acid (CHCA) (Sigma-Aldrich) was used as the matrix for intact precursor peptide. In general, the sample dissolved in 50 μL 0.1% MeOH, and then 2 μL sample was spotted and dried on the target. Subsequently, 2 μL matrix solution (5 mg/mL CHCA dissolved in 50% ACN and 0.1% TFA) was spotted on top of the sample. MALDI-TOF mass data was acquired using the following parameters. Tuning: reflectron; Mass range: 800-2000; Laser diameter: 100 μm; Laser power: 80-100 mV. Cytochrome C and TOFMixTM (SHIMADZU) was used for calibration.

1.7 Bioinformatic analysis

To obtain *ftchy* gene cluster from *F. tricinctum* CGMCC 3.4731, we used *chyA* protein sequence from *P. chrysogenum* to retrieve *chyA* orthologues by using local-blast in public fungal genome from NCBI (National Center for Biotechnology Information) database and the private database of our lab. For gene cluster annotation of *ftchy*, 2ndFind program was used to predict the open reading frame and intron. The gene function was assigned based on NCBI Blast search. The domains of ftChyA were analyzed by *interpro* website. Active sites analyses of the ftChyA-C_1, ftChyA-C_T, ftChyE, ftChyD and ftChyM were performed using DNAMAN8.0 software, respectively. The conserved domain analysis of ftChyM and ftChyH were performed using NCBI Blast.

To analyze the phylogenetic relationships of ftChyA-C_1 and ftChyA-C_T domain, multiple sequence alignment of the ftChyA-C_1 and ftChyA-C_T domain sequences and other C domain sequences (TqaA, AmpA, AmpB, BenZ, PsyA, RoqA, IvoA, SimA, Aba1, EasA, FGSG_08209, FPSE_09183, Glip, Sirp, HasD, NFIA_064400, PENFLA_c013G03821) obtained from NCBI database were performed using ClustalW. Evolutionary analyses were conducted in MEGA7 software, and the phylogenetic tree was inferred by using neighbor-joining method.

TqaA: ADY16697.1, https://www.ncbi.nlm.nih.gov/protein/ADY16697.1.
AmpA: A0A1W6BT53.1, https://www.ncbi.nlm.nih.gov/protein/A0A1W6BT53.1.
AmpB: A0A1W6BT46.1, https://www.ncbi.nlm.nih.gov/protein/A0A1W6BT46.1.
BenZ: P9WEU9.1, https://www.ncbi.nlm.nih.gov/protein/P9WEU9.1.
PsyA: AMQ36132.1, https://www.ncbi.nlm.nih.gov/protein/AMQ36132.1.
RoqA: B6HJU6.1, https://www.ncbi.nlm.nih.gov/protein/B6HJU6.1.
IvoA: C8V7P4.1, https://www.ncbi.nlm.nih.gov/protein/C8V7P4.1.
SimA: CAA82227.1, https://www.ncbi.nlm.nih.gov/protein/CAA82227.1.
Aba1: ACJ04424.1, https://www.ncbi.nlm.nih.gov/protein/ACJ04424.1.
EasA: C8VPS9.1, https://www.ncbi.nlm.nih.gov/protein/C8VPS9.1.

FGSG_08209: I1RVD9.1, https://www.ncbi.nlm.nih.gov/protein/I1RVD9.1.
FPSE_09183: K3VDP2.1, https://www.ncbi.nlm.nih.gov/protein/K3VDP2.1.
Glip: Q4WMJ7.1, https://www.ncbi.nlm.nih.gov/protein/Q4WMJ7.1.
Sirp: Q6Q883.1, https://www.ncbi.nlm.nih.gov/protein/Q6Q883.1.
HasD: XP_754329.2, https://www.ncbi.nlm.nih.gov/protein/XP_754329.2.
NFIA_064400: XP_001263173.1, https://www.ncbi.nlm.nih.gov/protein/XP_001263173.1.
PENFLA_c013G03821: OQE22222.1, https://www.ncbi.nlm.nih.gov/protein/OQE22222.1.

The enzyme function initiative-enzyme similarity tool (EFI-EST) was used to generate protein sequence similarity networks (SSNs) of ftChyM with fungal α-ketoglutarate dependent dioxygenases or the ftChyA-C_1 and ftChyA-C_T with other fungal C domains. Cytoscape (v3.8.2) software was used for visualizing complex networks of ftChyM or ftChyA-C_1 and ftChyA-C_T.

2nd Find: http://biosyn.nih.go.jp/2ndFind/.
NCBI BLAST: https://blast.ncbi.nlm.nih.gov/Blast.cgi.
interpro website: http://www.ebi.ac.uk/interpro/search/sequence/.
initiative-enzyme similarity tool (EFI-EST): https://efi.igb.illinois.edu/efi-est/.

1.8 Purification and structural characterization of compounds

To isolate **1**, **4**, **5**, **6**, **7**, **8** and **9**, the *AN-ftchyACDEHM* transformants were cultured in 16 L solid CD-ST medium at 25 °C for 4 days. And then, the culture was extracted with ethyl acetate/acetone (v/v, 3/1) for three times. The organic extracts were evaporated to dryness. The extracts were separated by Medium pressure liquid chromatography (MPLC) Reveleris® X2 (BUCHI, Switzerland) with a linear gradient of 10% MeOH-H_2O for 10 min, 10%-50% MeOH-H_2O in 40 min, 50%-100% MeOH-H_2O in 15 min, followed by 100% MeOH-H_2O for 15 min with flow rate of 30 mL/min on C18 column. The fractions containing **1** further separated by semi-preparative HPLC (column: YMC-Pack ODS-A, 5 µm, 10 × 250 mm; solvent: MeOH-H_2O, 35:65; flow: 2.5 mL/min; detector: 210 nm; t_R = 21.0 min) to yield **1** (10 mg). The compound **4** was purified by HPLC (column: YMC-Pack ODS-A, 5 µm, 10 × 250 mm; solvent: MeCN-H_2O (water with 0.1% v/v formic acid), 26:74; flow: 2.5 mL/min; detector: 210 nm; t_R=18.0 min) to give **4** (2 mg). The compound **5** was purified by semi-preparative HPLC (column: YMC-Pack ODS-A, 5 µm, 10 × 250 mm; solvent: MeCN-H_2O, 10:90; flow: 2.5 mL/min; detector: 210 nm) to give **5** (4 mg, t_R = 35.0 min). The compound **6** was further purified by semi-preparative HPLC (column: YMC-Pack ODS-A, 5 µm, 10 × 250 mm; solvent: MeOH-H_2O (water with 0.1% v/v formic acid), 42:58; flow: 2.5 mL/min; detector: 210 nm; t_R = 30.0 min) to yield **6** (3.7 mg). The compound **7** further purified by semi-preparative HPLC (column: YMC-Pack ODS-A, 5 µm, 10 × 250 mm; solvent: MeOH-H_2O, 18:82; detector: 265 nm) to give **7** (12 mg, t_R = 33.0 min). The compound **8** (8.8 mg) was isolated using 11 % (v/v) MeCN-H_2O as the mobile phase by semi-preparative HPLC (column: YMC-Pack ODS-A, 5 µm, 10 × 250 mm; flow: 2.5 mL/min; detector: 210 nm; t_R=45.0 min). The compound **9** (10 mg) was separated

by semi-preparative HPLC (column: YMC-Pack ODS-A, 5 μm, 10 × 250 mm; MeCN-H_2O, 10:90; flow: 2.5 mL/min; detector: 210 nm; t_R=24.0 min).

To isolate **10**, the *AN-ftchyA* transformants were cultured in 4.8 L solid CD-ST medium containing 10 mM uridine, 5 mM uracil and 0.25 μg/mL riboflavin at 25 °C for 4 days. And then, the culture was extracted with MeOH for three times. The extracts were evaporated to dryness. The extracts were separated by Medium pressure liquid chromatography (MPLC) Reveleris® X2 (BUCHI, Switzerland) with a linear gradient of 10% MeOH-H_2O for 10 min, 10%-50% MeOH-H_2O in 30 min, 50%-100% MeOH-H_2O in 20 min, followed by 100% MeOH-H_2O for 20 min with flow rate of 25 mL/min on C18 column. The fractions containing **10** further separated by semi-preparative HPLC (column: YMC-Pack ODS-A, 5 μm, 10 × 250 mm; solvent: MeOH-H_2O (water with 0.1% v/v formic acid), 40:60; flow: 2.5 mL/min; detector: 210 nm; t_R = 18.0 min) to yield **10** (12 mg). ^{1}H NMR (400 MHz, DMSO-d_6): δ 12.25 (s, 1H), 8.64 (d, J = 6.5 Hz, 1H), 8.53 (dd, J = 8.6, 1.0 Hz, 1H), 7.94 (dd, J = 7.9, 1.7 Hz, 1H), 7.42 (td, J = 7.9, 1.7 Hz, 1H), 7.06 (td, J = 7.6, 1.2 Hz, 1H), 4.20 (m, 1H), 3.64 (dd, J = 9.4, 4.7 Hz, 1H), 2.54 (m, 1H), 2.45 (m, 1H), 2.18 (m, 1H), 1.82 (m, 1H), 1.35 (d, J = 7.4 Hz, 3H); ^{13}C NMR (100 MHz, DMSO-d_6): δ 171.7, 171.5, 171.4, 169.4, 139.3, 131.7, 131.3, 122.3, 122.0, 118.9, 52.0, 50.4, 29.9, 26.0, 17.3; UV/Vis: 221, 252, 302 nm; HRMS (m/z): $[M]^+$ calcd. for $C_{15}H_{20}N_3O_6$, 338.1347; found, 338.1346.

To isolate **3**, the large-scale *in vitro* biochemical assays of ftChyA with L-Ala and Ant were performed. The purified 10 μM ftChyA was converted to its *holo* form by incubation in 20 mM Tris-HCl (pH 7.5), 100 mM NaCl, 20 μM NpgA, 0.1 mM CoA and 10 mM $MgCl_2 \cdot 6H_2O$ in buffer C (pH 7.5) (total volume 52 mL) at 25 °C for 1 h. Reactions were initiated by the addition of 5 mM ATP, 1 mM Ant and 1 mM L-Ala. The reaction mixture was incubated at 25 °C overnight and then freeze-dried. The products were dissolved in MeOH and purified by semipreparative HPLC with a C18 column (YMC-Pack ODS-A, 5 μm, 10 × 250 mm; solvent: MeOH-H_2O (water with 0.02% v/v formic acid), 25:75; flow: 2.5 mL/min; detector: 210 nm; t_R = 12.5 min; 2 mg).

To isolate **11** and **2**, the large-scale *in vitro* biochemical assays of ftChyD with **10** and **3** were performed, respectively. The reaction mixture containing 20 μM ftChyD, 200 μM **10**, 10 mM $MgCl_6 \cdot H_2O$, 4 mM ATP, 2 mM L-Gln and 20 mM Tris-HCl (pH 7.5) in buffer C (pH 7.5) (total volume 75 mL) at 25 °C overnight. The reaction mixture containing 20 μM ftChyD and 200 μM **3** in buffer C (pH 7.5) (total volume 120 mL) under the same conditions. The reaction mixture was freeze-dried, and the products were dissolved in MeOH. Compound **11** was purified by semipreparative HPLC (column: YMC-Pack ODS-A, 5 μm, 10 × 250 mm; solvent: MeOH-H_2O (water with 0.1% v/v formic acid), 20:80; flow: 2.5 mL/min; detector: 210 nm; t_R = 22.5 min) to yield **11** (4.1 mg). ^{1}H NMR (400 MHz, DMSO-d_6): δ 11.94 (s, 1H), 8.71 (d, J = 6.6 Hz, 1H), 8.48 (dd, J = 8.4, 1.2 Hz, 1H), 8.27 (s, 1H), 7.91 (s, 1H), 7.77 (dd, J = 8.0, 1.6 Hz, 1H), 7.48 (td, J = 8.0, 1.5 Hz, 1H), 7.11 (td, J = 7.6, 1.2 Hz, 1H), 4.20 (m, 1H), 3.30 (t, J = 6.2 Hz, 1H), 2.44 (t, J = 7.7 Hz, 2H), 1.98 (m, 1H), 1.90 (m, 1H), 1.32 (d, J = 7.3

Hz, 3H); ^{13}C NMR (100 MHz, DMSO-d_6): δ 172.6, 171.6, 170.7, 170.0, 139.1, 132.0, 128.5, 122.4, 120.2, 119.9, 53.8, 50.2, 31.7, 26.6, 17.2; UV/Vis: 212, 250, 293 nm; HRMS (m/z): $[M]^+$ calcd. for $C_{15}H_{21}N_4O_5$, 337.1506; found, 337.1506.

Compound **2** was separated by semipreparative HPLC (column: YMC-Pack ODS-A, 5 μm, 10 × 250 mm; MeOH-H_2O (water with 0.02% v/v formic acid), 5:95; flow: 2.5 mL/min; detector: 210 nm) to yield **2** (3.5 mg, t_R=12.5 min).

To isolate **14** from large-scale spontaneous conversion of **2** in Tris-HCl buffer (pH 7.5). Compound **2** (3 mg) was added to 500 mL buffer C (pH 7.5). The reaction mixture was kept at 25 °C for 36 h and extracted with ethyl acetate 4 times. The ethyl acetate layer was evaporated until dry and separated by semipreparative HPLC (column: YMC-Pack ODS-A, 5 μm, 10 × 250 mm; solvent: MeCN-H_2O (water with 0.1% v/v formic acid), 12:88; flow: 2.5 mL/min; detector: 210 nm; t_R = 10 min) to yield **14** (1.6 mg).

2. Supplementary Tables

Supplementary Table 1. Plasmids used in this study.

Name	Description	Enzyme site	Aim
pIM 3201	*ftchyA* gDNA with downstream 387 bp, in pANR	*BamH* I	*A. nidulans* overexpression
pIM 3202	*ftchyC* gDNA with downstream 425 bp, *ftchyD* gDNA with downstream 264 bp, *ftchyE* gDNA with downstream 395 bp, in pANU	*Not* I	*A. nidulans* overexpression
pIM 3203	*ftchyM* gDNA with downstream 267 bp, *ftchyH* gDNA with downstream 426 bp, in pANP	*BamH* I	*A. nidulans* overexpression
pIM 3204	*ftchyC* gDNA with downstream 425 bp, *ftchyD* gDNA with downstream 264 bp, in pANU	*Not* I	*A. nidulans* overexpression
pIM 3205	*ftchyH* gDNA with downstream 426 bp, in pANP	*BamH* I	*A. nidulans* overexpression
pIM 3206	*ftchyM* gDNA with downstream 267 bp, in pANP	*BamH* I	*A. nidulans* overexpression
pIM 3207	*ftchyA* cDNA in pYEU with Flag and His tag	*Spe* I-*Pml* I	To provide *ftchyA* gene
pIM 3208	*ftchyA* cDNA in pColdI with His tag	*BamH* I-*EcoR* I	*E. coli* overexpression
pIM 3209	$ftchyA\text{-}C_{1}\text{-}H_{987}A$ cDNA in pYEU with Flag and His tag	*Spe* I-*Pml* I	To provide $ftchyA\text{-}C_{1}\text{-}H_{987}A$ gene
pIM 3210	$ftchyA\text{-}C_{T}\text{-}H_{2074}A$ cDNA in pYEU with Flag and His tag	*Spe* I-*Pml* I	To provide $ftchyA\text{-}C_{T}\text{-}H_{2075}A$ gene
pIM 3211	$ftchyA\text{-}C_{1}^{*}$ ($A_{986}A_{987}xxxA_{991}$) cDNA in pYEU with Flag and His tag	*Spe* I-*Pml* I	To provide $ftchyA\text{-}C_{1}^{*}$ gene
pIM 3212	$ftchyA\text{-}C_{1}\text{-}H_{987}A$ cDNA in pColdI with His tag	*BamH* I-*EcoR* I	To prove the active residues of ftChyA-C_1 domain
pIM 3213	$ftchyA\text{-}C_{T}\text{-}H_{2074}A$ cDNA in pColdI with His tag	*BamH* I-*EcoR* I	To prove the active residues of ftChyA-C_T domain
pIM 3214	$ftchyA\text{-}C_{1}^{*}$ ($A_{986}A_{987}xxxA_{991}$) cDNA in pColdI with His tag	*BamH* I-*EcoR* I	To prove the active residues of ftChyA-C_1 domain
pIM 3215	$ftchyA\text{-}A_{1}$ cDNA in pColdI with His tag	*Kpn* I-*EcoR* I	*E. coli* overexpression ftChyA-A_1 domain alone
pIM 3216	$ftchyA\text{-}A_{2}$ cDNA in pQ8 with MBP tag	*EcoR* I-*Not* I	*E. coli* overexpression ftChyA-A_2 domain alone
pIM 3217	$ftchyA\text{-}C_{T}$ cDNA in pColdI with His tag	*BamH* I-*EcoR* I	*E. coli* overexpression ftChyA-C_T domain alone
pIM 3218	$ftchyA\Delta C_{T}$ cDNA in pColdI with His tag	*BamH* I-*EcoR* I	To prove the ftChyA-C_T domain
pIM 3219	$ftchyA\text{-}A_{1}T_{1}$ cDNA in pColdI with His tag	*Kpn* I-*EcoR* I	*E. coli* overexpression ftChyA-A_1T_1 domain
pIM 3220	*NpgA* cDNA in pET28a without His tag	*Hind* III-*Nco* I	*E. coli* overexpression

pIM 3221	*NpgA* cDNA in pColdI with His tag	*Sac* I-*Hind* III	*E. coli* overexpression
pIM 3222	*ftchyC* cDNA in pColdI with His tag	*Kpn* I-*EcoR* I	*E. coli* overexpression
pIM 3223	*ftchyD* cDNA in pET-Duet with His tag	*BamH* I-*Not* I	*E. coli* overexpression
pIM 3224	*ftchyE* cDNA in pQ8 with MBP tag	*EcoR* I-*Not* I	*E. coli* overexpression
pIM 3225	*ftchyM* cDNA in pColdI with His tag	*Kpn* I-*BamH* I	*E. coli* overexpression
pIM 3226	*ftchyH* cDNA in pColdI with His tag	*Kpn* I-*Hind* III	*E. coli* overexpression
pIM 3227	*ftchyH* cDNA in pQ8 with MBP tag	*EcoR* I-*Not* I	*E. coli* overexpression
pIM 3228	*ftchyH* cDNA in pGEX-4t-1 with GST tag	*EcoR* I-*Not* I	*E. coli* overexpression
pIM 3229	*ftchyH* cDNA in pYEU with Flag and His tag	*Spe* I-*Pml* I	*S. cerevisiae* overexpression

Supplementary Table 2. Products from gene (*chyC*, *chyE* and *chyH*) individual KO mutant of *P. chrysogenum*[1].

Products	Chemical Formula	Molecular Weight	*KO chyC*	*KO chyE*	*KO chyH*
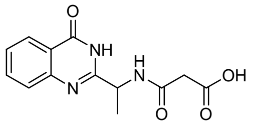	$C_{13}H_{13}N_3O_4$	275	√		√
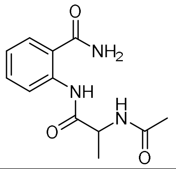	$C_{12}H_{15}N_3O_3$	249	√	√	√
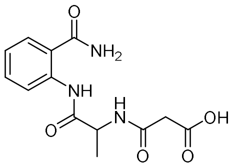	$C_{13}H_{15}N_3O_5$	293	√	√	√
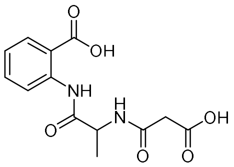	$C_{13}H_{14}N_2O_6$	294	√	√	√
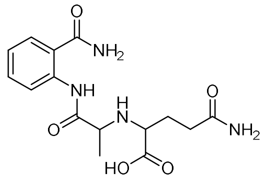	$C_{15}H_{20}N_4O_5$	336		√	√
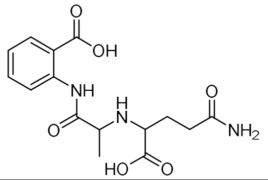	$C_{15}H_{19}N_3O_6$	337	√	√	√
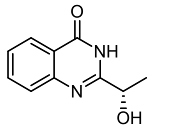	$C_{10}H_{10}N_2O_2$	190	√	√	√

Supplementary Table 3. Analysis of *ftchy* gene cluster from *F. tricinctum*.

Gene	Size(bp)	Gene function	*P. chrysogenum* (identity/similarity)	*F. graminearum* (identity/similarity)
ftchyA	7274	NRPS (A_1-T_1-C_1-A_2-T_2-C_T)	64/76	64/78
ftchyC	786	short-chain dehydrogenase (SDR)	79/87	82/91
ftchyD	2287	class II amidotransferase	80/89	87/95
ftchyE	824	glutaminase	31/47	56/74
ftchyH	1743	flavin-dependent oxidase	69/82	74/84
ftchyM	1179	α-KG dioxygenase	71/82	73/83

Supplementary Table 4. HRMS data of compounds in this study.

Com.	Structure	Ion Formula	Meas. *m/z* $[M+H]^+$	Meas. *m/z* $[M+Na]^+$	Calc. *m/z* $[M+H]^+$	Calc. *m/z* $[M+Na]^+$	Err (ppm)
**1**	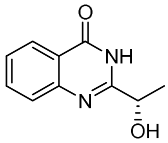	$C_{10}H_{11}N_2O_2$	191.0815		191.0815		0.1
**2**	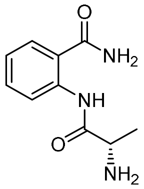	$C_{10}H_{14}N_3O_2$	208.1081		208.1081		-0.3
**3**	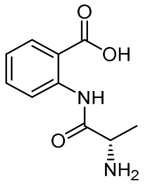	$C_{10}H_{13}N_2O_3$	209.0921		209.0921		-0.4
**4**	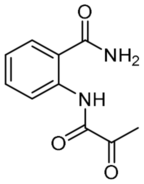	$C_{10}H_{10}N_2NaO_3$		229.0584		229.0584	0.0
**5**	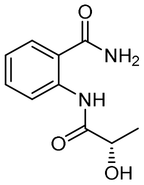	$C_{10}H_{12}N_2NaO_3$		231.0741		231.0740	-0.3
**6**	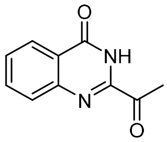	$C_{10}H_9N_2O_2$	189.0655		189.0659		2.0
**7**	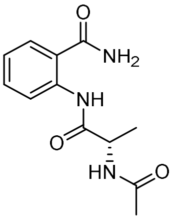	$C_{12}H_{15}N_3NaO_3$		272.1007		272.1006	-0.3
**8**	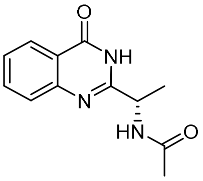	$C_{12}H_{14}N_3O_2$	232.1082		232.1081		-0.8
**9**	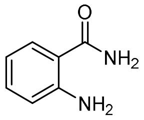	$C_7H_9N_2O$	137.0706		137.0709		2.8

**10**	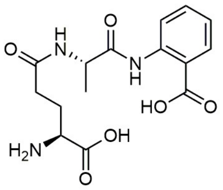	$C_{15}H_{20}N_3O_6$	338.1346		338.1347		0.2
**11**	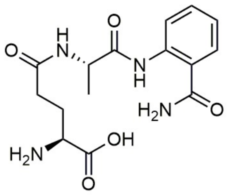	$C_{15}H_{21}N_4O_5$	337.1506		337.1506		0.2
**14**	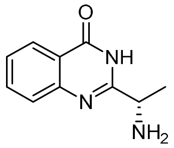	$C_{10}H_{12}N_3O$	190.0977		190.0975		-0.9
Ant-Me-**3**	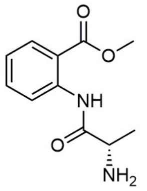	$C_{11}H_{15}N_2O_3$	223.1077		223.1077		0.1
Ant-Me-**10**	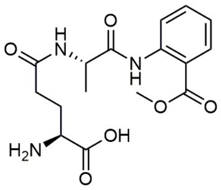	$C_{16}H_{21}N_3NaO_6$		374.1323		374.1323	-0.1

Supplementary Table 5. NMR data of compound **1** in DMSO-d_6.

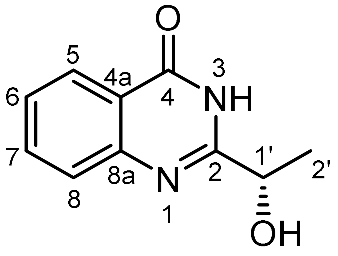

(400 MHz for ^{1}H NMR, 100 MHz for ^{13}C NMR)

NO.	*δ*c, type[2]	*δ*c, type	*δ*H (mult, *J* in Hz)[2]	*δ*H (mult, *J* in Hz)
1				
2	159.6, C	159.9, C		
3			11.79 (1H, br s, NH)	11.83 (1H, br s, NH)
4	161.4, C	161.7, C		
4a	121.2, C	121.2, C		
5	125.7, CH	125.8, CH	8.10 (1H, d, 7.6)	8.10 (1H, dd, 8.0,1.5)
6	126.2, CH	126.8, CH	7.48 (1H, t, 7.5)	7.48 (1H, t, 8.0)
7	134.3, CH	134.4, CH	7.79 (1H, t, 7.6)	7.79 (1H, td, 8.0,1.5)
8	126.9, CH	126.3, CH	7.63 (1H, d, 8.1)	7.64 (1H, d, 8.0)
8a	148.4, C	148.3, C		
1′	67.1, CH	67.2, CH	4.59 (1H, m)	4.59 (1H, q, 6.6)
2′	21.5, CH_3	21.6, CH_3	1.43 (3H, d, 6.6)	1.43 (3H, d, 6.6)
1′-OH			5.65 (1H, d, 7.6)	5.67 (1H, br s)

Supplementary Table 6. NMR data of compound **2** in DMSO-d_6.

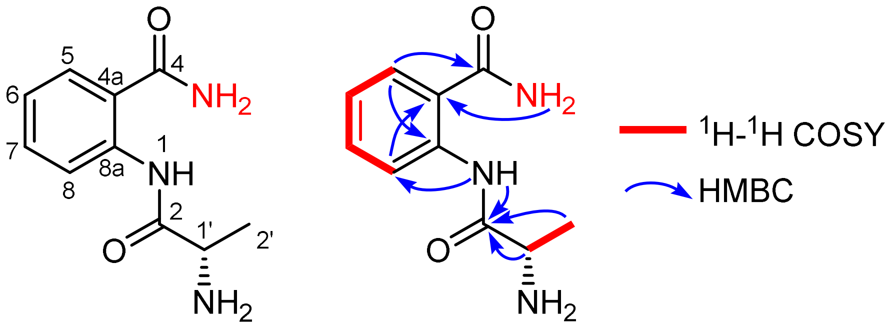

(400 MHz for ^1H NMR, 100 MHz for ^{13}C NMR)

NO.	δ_H (mult, *J* in Hz)	δ_C, type	COSY	HMBC
1	12.01 (1H, s)			C2, C8
2		167.9, C		
4		170.4, C		
4a		120.9, C		
5	7.84 (1H, dd, 7.9, 1.5)	128.7, CH	H6	C4, C7, C8a
6	7.20 (1H, td, 8.0, 1.2)	123.5, CH	H5, H7	C4a, C5, C8
7	7.55 (1H, td, 8.0, 1.5)	132.2, CH	H6, H8	C5, C8, C8a
8	8.32 (1H, dd, 8.3, 1.2)	120.8, CH	H7	C4a, C6
8a		138.3, C		
1′	4.18 (1H, q, 7.0)	49.3, CH	H2′	C2′, C2
2′	1.49 (3H, d, 7.0)	16.4, CH_3	H1′	C1′, C2
1′-NH_2	8.31 (2H, overlapped)			
4-NH_a	7.79 (1H, s)			C4a
4-NH_b	8.31 (1H, overlapped)			

Supplementary Table 7. NMR data of compound **3** in DMSO-d_6.

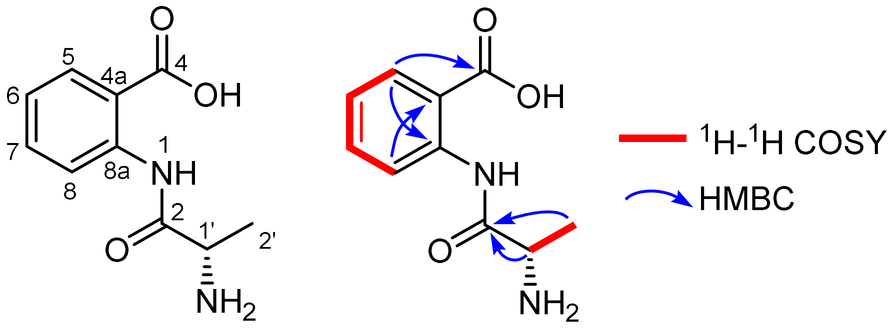

(400 MHz for ^{1}H NMR, 100 MHz for ^{13}C NMR)

NO.	δ_H (mult, J in Hz)	δ_C, type	COSY	HMBC
1	11.33 (1H, s)			
2		168.2, C		
4		169.0, C		
4a		118.9, C		
5	8.00 (1H, dd, 7.8, 1.2)	131.1, CH	H6	C4, C7, C8a
6	7.24 (1H, m)	123.8, CH	H5, H7	C4a
7	7.63 (1H, m)	133.7, CH	H6, H8	C5, C8a
8	8.26 (1H, dd, 8.4, 1.2)	121.1, CH	H7	C4a, C6
8a		139.0, C		
1′	4.22 (1H, q, 7.0)	49.3, CH	H2′	C2′, C2
2′	1.51 (3H, d, 6.8)	16.5, CH_3	H1′	C1′, C2

Supplementary Table 8. NMR data of compound **4** in DMSO-d_6.

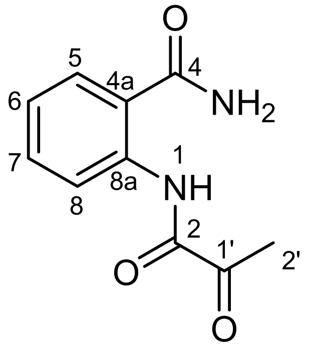

(400 MHz for ^{1}H NMR, 100 MHz for ^{13}C NMR)

NO.	δc, type[3]	δc, type	δ_H (mult, J in Hz)[3]	δ_H (mult, J in Hz)
1			12.65 (1H, s)	12.66 (1H, s)
2	158.6, C	158.7, C		
4	170.2, C	170.3, C		
4a	120.6, C	120.7, C		
5	128.7, CH	128.8, CH	7.84 (1H, dd, 8.2, 1.2)	7.85 (1H, d, 8.0)
6	123.3, CH	123.4, CH	7.20 (1H, td, 8.2, 1.2)	7.21 (1H, t, 8.0)
7	132.2, CH	132.3, CH	7.56 (1H, td, 8.2, 1.2)	7.57 (1H, t, 8.0)
8	119.8, CH	119.8, CH	8.58 (1H, dd, 8.2, 1.2)	8.59 (1H, d, 7.8)
8a	138.0, C	138.1, C		
1′	196.3, C	196.4, C		
2′	24.1, CH_3	24.1, CH_3	2.43 (3H, s)	2.42 (3H, s)
4-NH_a			8.28 (1H, br s)	8.31 (1H, br s)
4-NH_b			7.73 (1H, br s)	7.77 (1H, br s)

Supplementary Table 9. NMR data of compound **5** in DMSO-d_6.

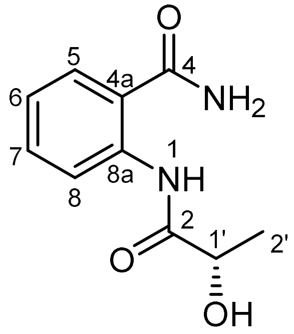

(400 MHz for ^{1}H NMR, 100 MHz for ^{13}C NMR)

NO.	δ_C, type[4]	δ_C, type	δ_H (mult, J in Hz)[4]	δ_H (mult, J in Hz)
1			11.88 (1H, s)	11.99 (1H, s)
2	174.7, C	174.1, C		
4	170.8, C	170.2, C		
4a	121.4, C	120.9, C		
5	128.8, CH	128.5, CH	7.70 (1H, dd, 7.8, 1.5)	7.74 (1H, dd, 7.8, 1.0)
6	123.2, CH	122.4, CH	7.11 (1H, t, 7.8)	7.11 (1H, td, 8.0, 1.0)
7	132.4, CH	131.8, CH	7.46 (1H, dt, 7.8, 1.5)	7.47 (1H, td, 8.0, 1.1)
8	120.4, CH	119.8, CH	8.45 (1H, d, 7.8)	8.57 (1H, d, 8.4)
8a	138.7, C	138.7, C		
1′	68.3, CH	67.9, CH	4.09 (1H, q, 7.0)	4.10 (1H, m)
2′	21.2, CH_3	20.8, CH_3	1.28 (3H, d, 7.0)	1.30 (3H, d, 6.8)
1′-OH			6.05 (1H, br s)	5.97 (1H, d, 5.0)
4-NH_a			8.13 (1H, br s)	8.16 (1H, br s)
4-NH_b			7.05 (1H, br s)	7.60 (1H, br s)

.

Supplementary Table 10. NMR data of compound **6** in DMSO-d_6.

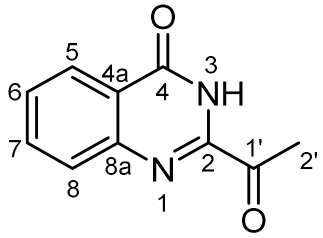

(400 MHz for ^{1}H NMR, 100 MHz for ^{13}C NMR)

NO.	δc, type[5]	δc, type	δ_H (mult, J in Hz)[5]	δ_H (mult, J in Hz)
1				
2	147.1, C	147.2, C		
3			12.20 (1H, br s)	12.26 (1H, br s)
4	159.9, C	160.8, C		
4a	123.6, C	123.2, C		
5	126.1, CH	126.1, CH	8.20 (1H, m, H-Ar)	8.19 (1H, dd, 7.9, 1.5)
6	128.8, CH	128.9, CH		7.66 (1H, td, 8.2, 1.5)
7	134.7, CH	134.8, CH	7.40-7.90 (3H, m)	7.91 (1H, td, 8.2, 1.5)
8	128.5, CH	128.5, CH		7.85 (1H, dd, 7.9, 1.5)
8a	147.3, C	147.4, C		
1′	194.0, C	193.9, C		
2′	24.7, CH_3	24.7, CH_3	2.70 (3H, s)	2.64 (3H, s)

Supplementary Table 11. NMR data of compound **7** in DMSO-d_6.

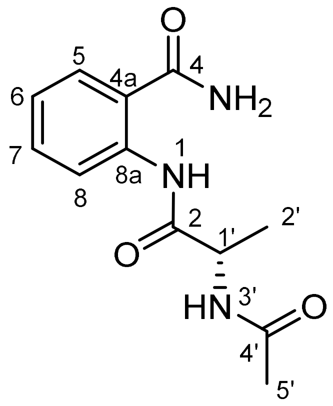

(400 MHz for ^{1}H NMR, 100 MHz for ^{13}C NMR)

NO.	*δ*c, type[6]	*δ*c, type	*δ*H (mult, *J* in Hz)[6]	*δ*H (mult, *J* in Hz)
1			12.05 (1H, s)	12.09 (1H, s)
2	171.8, C	171.7, C		
4	170.7, C	170.6, C		
4a	119.7, C	119.6, C		
5	128.7, CH	128.6, CH	7.78 (1H, dd, 7.8, 1.3)	7.80 (1H, dd, 7.8, 0.8)
6	122.4, CH	122.4, CH	7.10 (1H, dd, 7.8, 0.8)	7.11 (1H, td, 7.8, 0.9)
7	132.3, CH	132.2, CH	7.48 (1H, dd, 7.9, 1.3)	7.49 (1H, td, 8.4, 1.2)
8	119.7, CH	119.7, CH	8.53 (1H, dd, 7.9, 0.8)	8.55 (1H, d, 8.4)
8a	139.5, C	139.5, C		
1′	50.1, CH	50.0, CH	4.20 (1H, dq, 7.3, 6.8)	4.21 (1H, m)
2′	17.3, CH_3	17.3, CH_3	1.31 (3H, d, 7.3)	1.31 (1H, d, 7.3)
3′			8.47 (1H, d, 6.8)	8.51 (1H, d, 6.8)
4′	169.8, C	169.7, C		
5′	22.7, CH_3	22.6, CH_3	1.94 (3H, s)	1.96 (3H, s)
4-NH_a			8.23 (1H, br s)	8.26 (1H, br s)
4-NH_b			7.69 (1H, br s)	7.73 (1H, br s)

Supplementary Table 12. NMR data of compound **8** in DMSO-d_6.

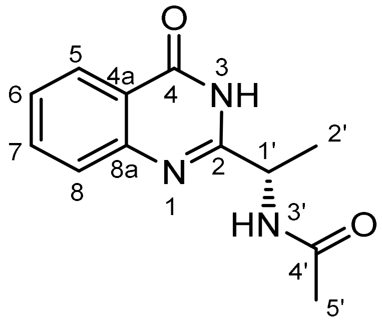

(400 MHz for ^{1}H NMR, 100 MHz for ^{13}C NMR)

NO.	***δ*c, type**[7]	***δ*c, type**	***δ*H (mult, *J* in Hz)**[7]	***δ*H (mult, *J* in Hz)**
1				
2	159.0, C	158.5, C		
3			12.16 (1H, s)	12.18 (1H, s)
4	162.1, C	161.6, C		
4a	121.6, C	121.1, C		
5	127.4, CH	126.9, CH	8.08 (1H, d, 8.0)	8.09 (1H, dd, 7.8, 1.0)
6	126.8, CH	126.3, CH	7.48 (1H, t, 7.5)	7.48 (1H, t, 7.3)
7	134.9, CH	134.4, CH	7.78 (1H, t, 7.5)	7.79 (1H, td, 7.8, 1.2)
8	126.3, CH	125.8, CH	7.61 (1H, d, 8.0)	7.62 (1H, d, 8.1)
8a	149.1, C	148.6, C		
1′	48.2, CH	47.7, CH	4.72 (1H, m)	4.72 (1H, m)
2′	19.7, CH_3	19.2, CH_3	1.38 (3H, d, 7.2)	1.39 (3H, d, 7.0)
3′			8.32 (1H, d, 7.05)	8.34 (1H, d, 7.0)
4′	169.8, C	169.3, C		
5′	23.0, CH_3	22.5, CH_3	1.87 (3H, s)	1.88 (3H, s)

Supplementary Table 13. NMR data of compound **9** in DMSO-d_6.

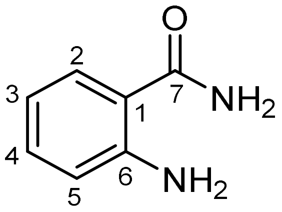

(400 MHz for ^{1}H NMR, 100 MHz for ^{13}C NMR)

NO.	δ_C, type[8]	δ_C, type	δ_H (mult, *J* in Hz)[8]	δ_H (mult, *J* in Hz)
1	113.7, C	113.7, C		
2	150.1, CH	150.2, CH	6.67 (1H, dd, 7.8, 1.3)	6.67 (1H, dd, 8.2, 1.0)
3	114.3, CH	114.4, CH	7.12 (1H, td, 7.8, 1.3)	7.12 (1H, td, 8.0, 1.4)
4	131.8, CH	131.9, CH	6.47 (1H, td, 7.8, 1.3)	6.47 (1H, td, 8.2, 1.0)
5	116.3, CH	116.4, CH	7.52 (1H, dd, 7.8, 1.3)	7.52 (1H, dd, 8.0, 1.4)
6	128.7, C	128.7, C		
7	171.3, C	171.4, C		
-CONH$_a$			7.68 (1H, br s)	7.67 (1H, br s)
-CONH$_b$			7.01 (1H, br s)	7.02 (1H, br s)
ArNH$_2$			6.53 (2H, br s)	6.53 (2H, br s)

Supplementary Table 14. NMR data of compound **10** in DMSO-d_6.

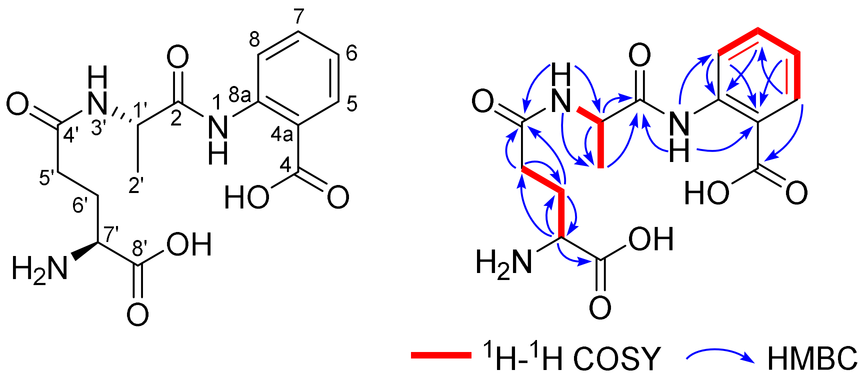

(400 MHz for ^{1}H NMR, 100 MHz for ^{13}C NMR)

NO.	δ_H (mult, J in Hz)	δ_C, type	COSY	HMBC
1	12.25 (s)			C8, C4a, C2
2		171.5, C		
4		169.4, C		
4a		122.0, C		
5	7.94 (dd, 7.9, 1.7)	131.3, CH	H6	C7, C8, C4
6	7.06 (td, 7.6, 1.2)	122.3, CH	H7, H5	C8, C4a, C7, C8a
7	7.42 (td, 7.9, 1.7)	131.7, CH	H6, H8	C8, C6, C5, C8a
8	8.53 (dd, 8.6, 1.0)	118.9, CH	H7	C8a, C6, C5, C4a
8a		139.3, C		
1′	4.20 (m)	50.4, CH	H2′	C2′, C2
2′	1.35 (d, 7.4)	17.3, CH_3		C1′, C2
3′	8.64 (d, 6.5)		H1′	C2′, C1′, C4′
4′		171.4, C		
5′a	2.54 (m)	29.9, CH_2		C6′, C7′, C4′
5′b	2.45 (m)			C6′, C7′, C4′
6′a	2.18 (m)	26.0, CH_2	H6′b, H5′a, H5′b	C7′, C4′, C5
6′b	1.82 (m)			C7′, C4′
7′	3.64 (dd, 9.4, 4.7)	52.0, CH	H6′a, H6′b	C6′, C5′, C8′
8′		171.7, C		

Supplementary Table 15. NMR data of compound **11** in DMSO-d_6

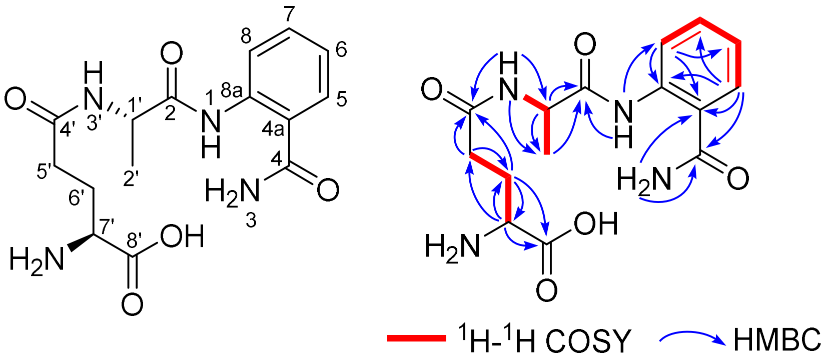

(400 MHz for ^{1}H NMR, 100 MHz for ^{13}C NMR)

NO.	δ_H (mult, J in Hz)	δ_C, type	COSY	HMBC
1	11.94 (s)			C8, C2
2		171.6, C		
3a	8.27 (s)			C4
3b	7.91 (s)			C4, C4a
4		170.7, C		
4a		120.2, C		
5	7.77 (dd, 8.0, 1.6)	128.5, CH	H6	C4a, C7, C8a, C4
6	7.11 (td, 7.6, 1.2)	122.4, CH	H7, H5	C4a, C5, C7
7	7.48 (td, 8.0, 1.5)	132.0, CH	H6, H8	C8, C5, C8a
8	8.48 (dd, 8.4, 1.2)	119.9, CH	H7	C4a, C6, C8a
8a		139.1, C		
1′	4.20 (m)	50.2, CH		C2′, C2
2′	1.32 (d, 7.3)	17.2, CH_3	H1′	C2, C1′
3′	8.71 (d, 6.6)		H1′	C2′, C1′, C4′
4′		172.6, C		
5′	2.44 (t, 7.7)	31.7, CH_2	H6′a, H6′b	C6′, C7′, C4′
6′a	1.98 (m)	26.6, CH_2	H7′	C5′, C7′, C8′, C4′
6′b	1.90 (m)		H7′	C5′, C7′, C8′, C4′
7′	3.30 (t, 6.2)	53.8, CH		C6′, C5′, C8′
8′		170.0, C		

Supplementary Table 16. NMR data of compound **14** in DMSO-d_6.

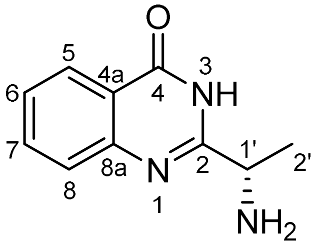

(400 MHz for ^{1}H NMR, 100 MHz for ^{13}C NMR)

NO.	δ_C, type	δ_H (mult, J in Hz)
2	160.7, C	
4	161.8, C	
4a	121.1, C	
5	125.8, CH	8.10 (1H, m)
6	126.8, CH	7.50 (1H, m)
7	134.4, CH	7.80 (1H, m)
8	126.2, CH	7.63 (1H, m)
8a	148.5, C	
1′	49.8, C	3.90 (1H, m)
2′	21.5, CH_3	1.36 (3H, s)
1′-NH_a		8.26 (1H, br s)

3. Supplementary Figures

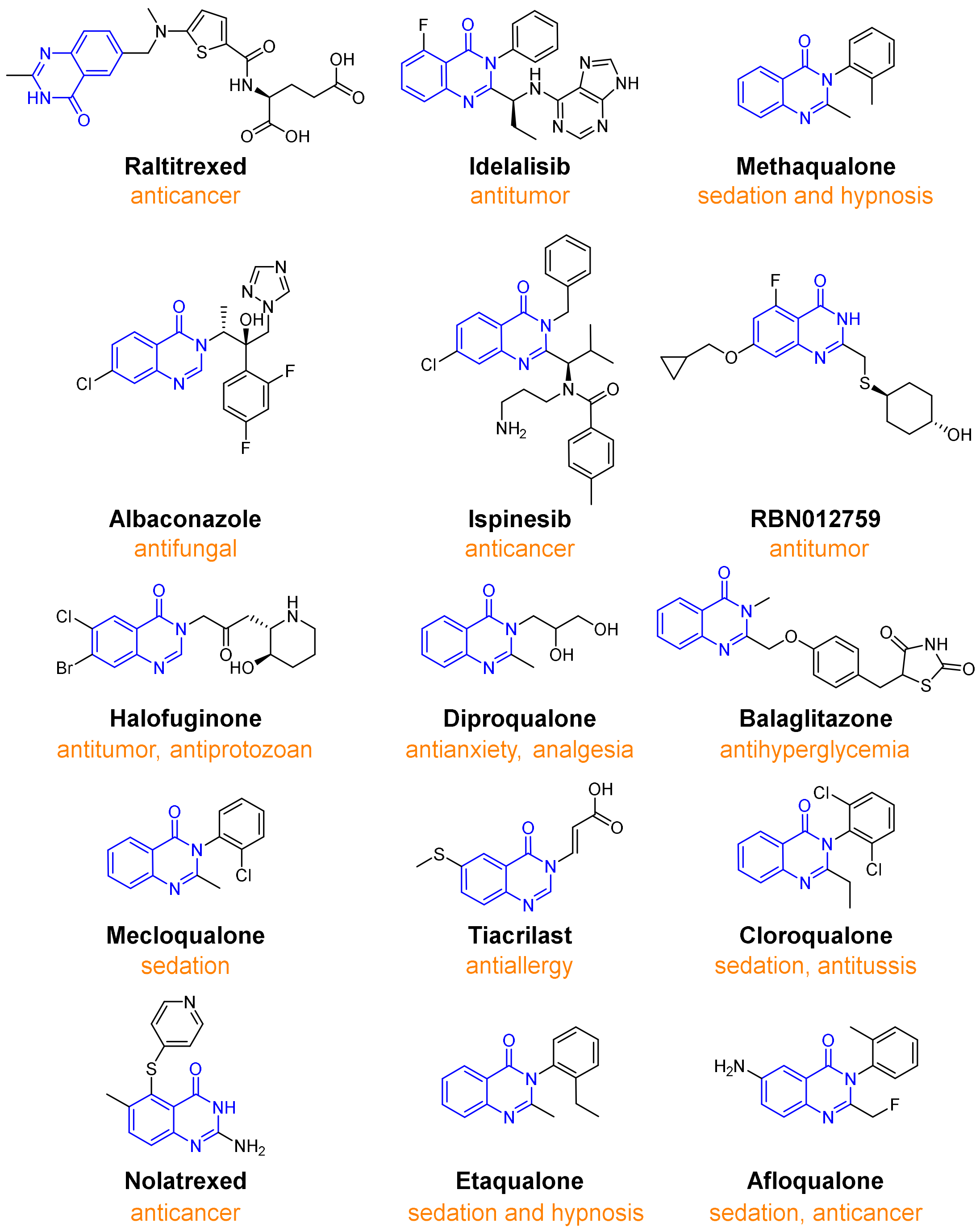

Supplementary Fig. 1 Marketed drugs with 4(3*H*)-quinazolinone scaffold.

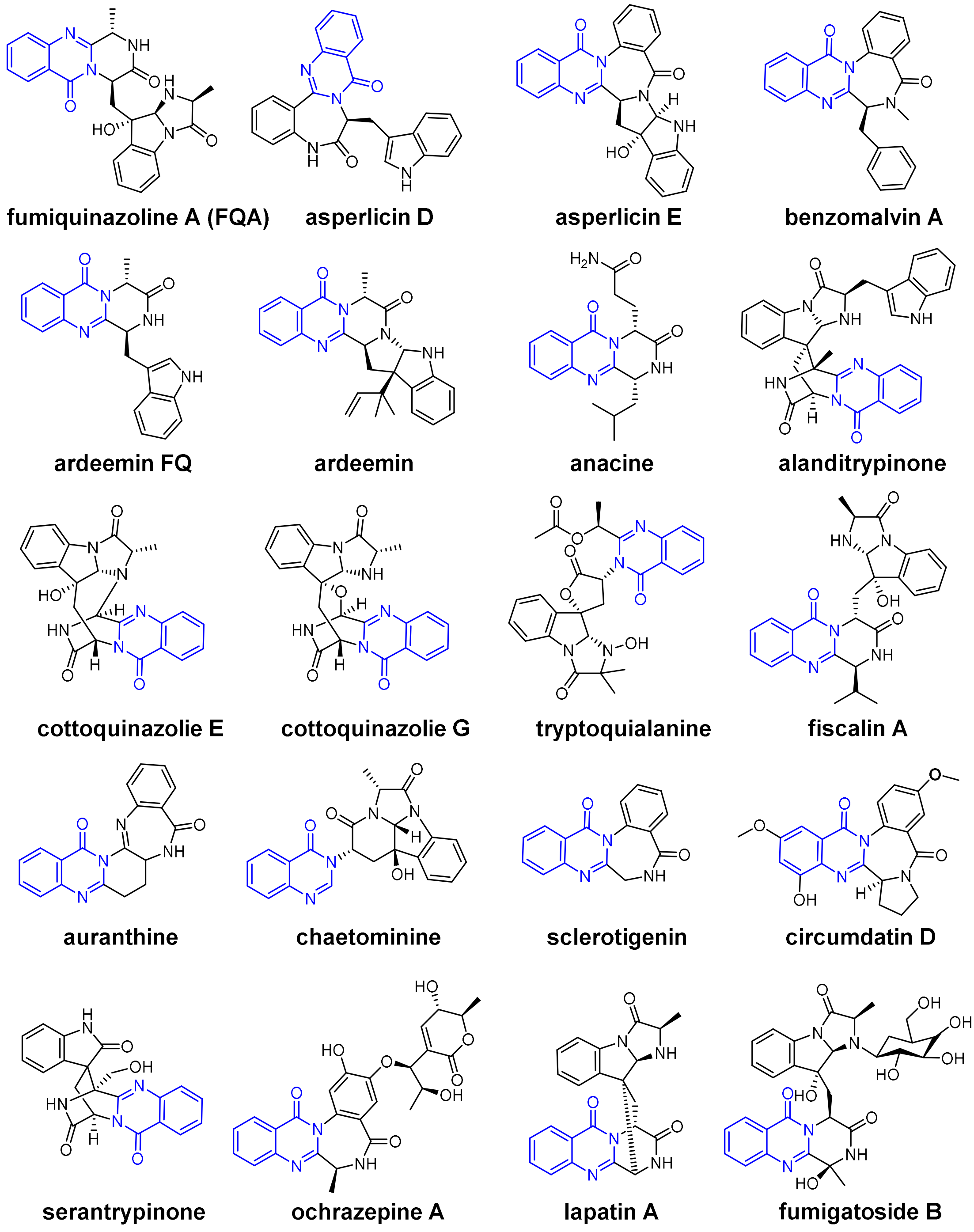

Supplementary Fig. 2 Representative natural 4(3*H*)-quinazolinone peptidyl alkaloids from fungi.

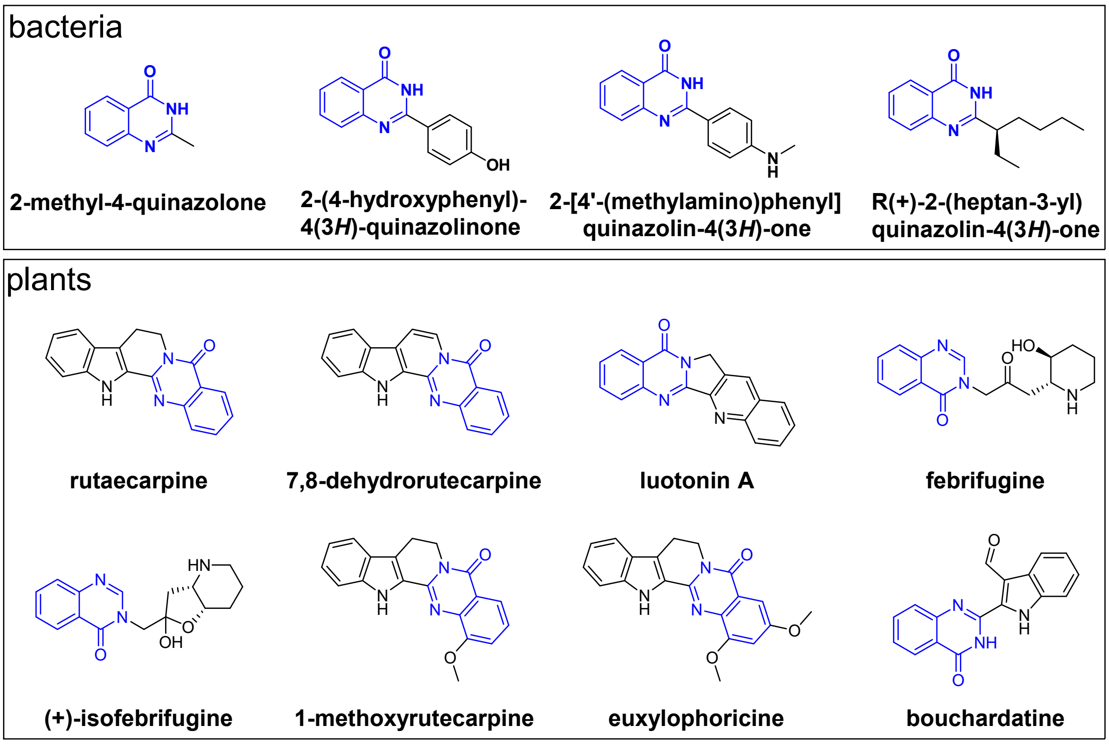

Supplementary Fig. 3 Representative natural 4(3*H*)-quinazolinone alkaloids from plants and bacteria.

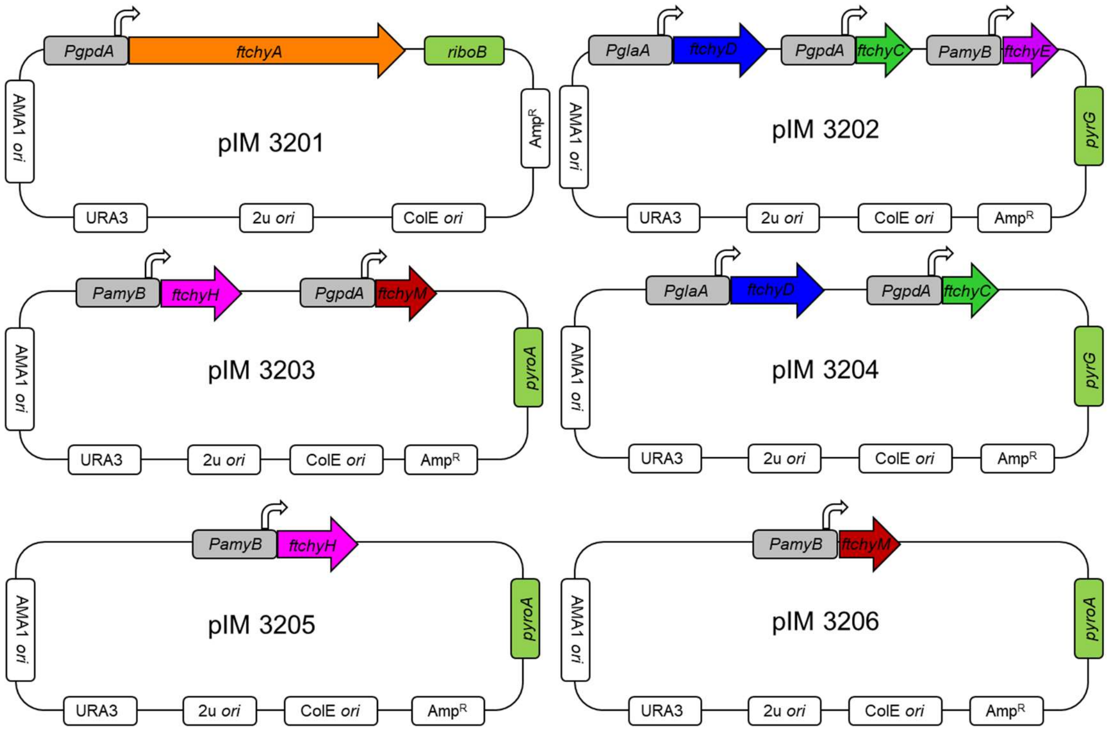

Supplementary Fig. 4 Construction of plasmids for overexpression of *ftchy* cluster in *A. nidulans*.

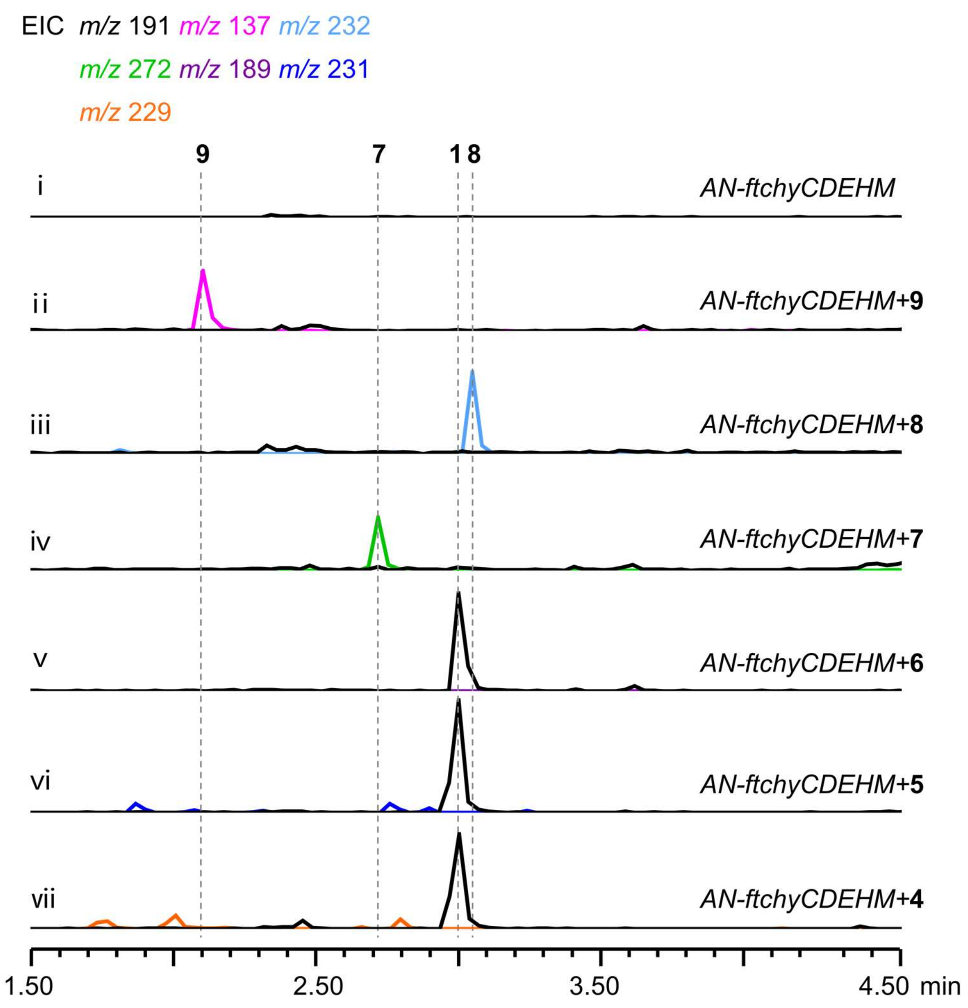

Supplementary Fig. 5 Confirmation of the on-pathway intermediates of **1** by chemical feeding experiments. The extracted ion chromatograms (EICs) were extracted at *m/z* 191 $[M + H]^+$ for **1**, *m/z* 229 $[M + Na]^+$ for **4**, *m/z* 231 $[M + Na]^+$ for **5**, *m/z* 189 $[M + H]^+$ for **6**, *m/z* 272 $[M + Na]^+$ for **7**, *m/z* 232 $[M + H]^+$ for **8** and *m/z* 137 $[M + H]^+$ for **9**.

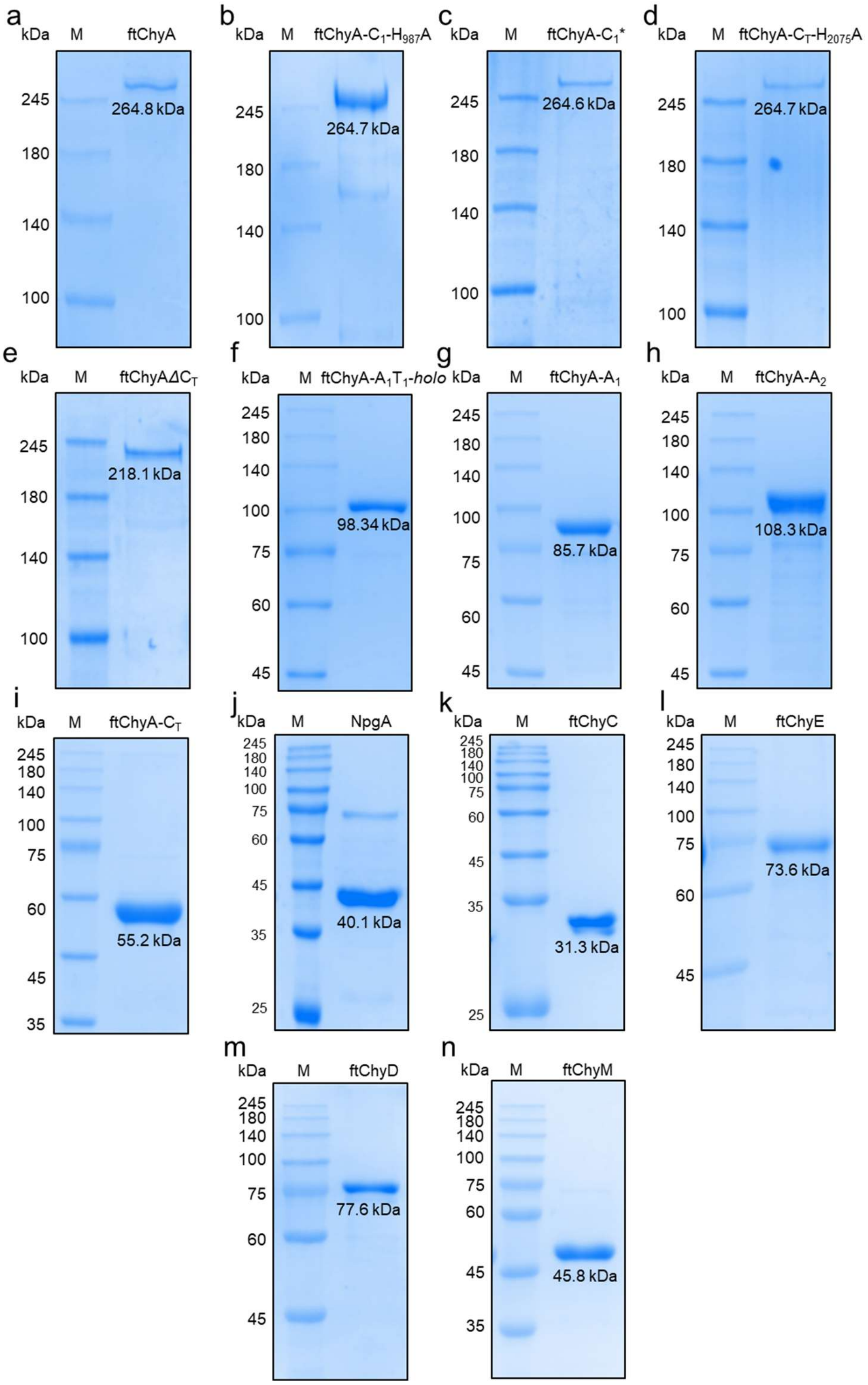

Supplementary Fig. 6 SDS-PAGE of proteins purified from *E. coli* BL21 in this study. (**a**) ftChyA (264.8 kDa). (**b**) ftChyA-C_1-H_{987}A (264.7 kDa). (**c**) ftChyA-C_1* (264.6 kDa). (**d**) ftChyA-C_T-H_{2075}A (264.7 kDa). (**e**) ftChyAΔC_T (218.1 kDa). (**f**) ftChyA-A_1T_1-*holo* (98.34 kDa). (**g**) ftChyA-A_1 (85.7 kDa). (**h**) ftChyA-A_2 (108.3 kDa) with N-MBP tag. (**i**) ftChyA-C_T (55.2 kDa). (**j**) NpgA (40.1 kDa). (**k**) ftChyC (31.3 kDa). (**l**) ftChyE (73.6 kDa) with N-MBP tag. (**m**) ftChyD (77.6 kDa). (**n**) ftChyM (45.8 kDa). M: marker. All experiments were repeated independently more than three times with similar results.

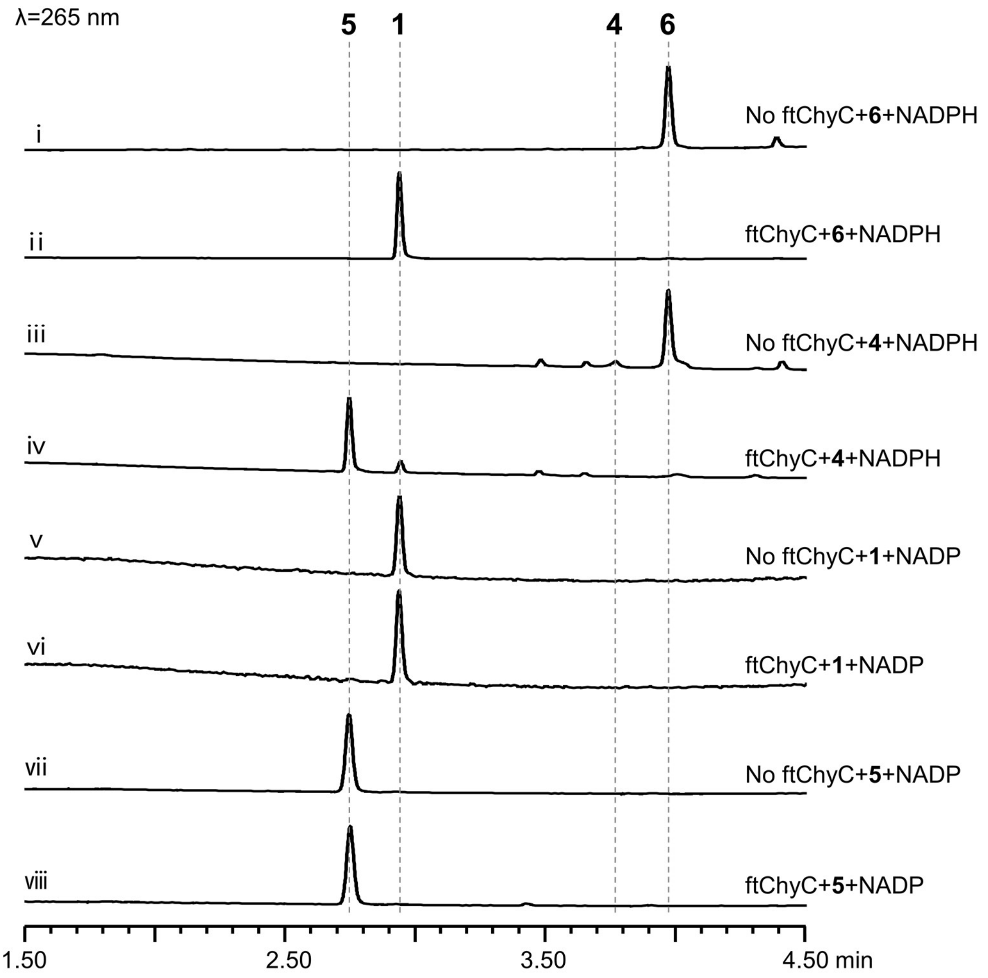

Supplementary Fig. 7 ftChyC-catalysed reduction or dehydrogenation reactions with different substrates. The spontaneous cyclization of **4** to form **6** is observed in control experiment.

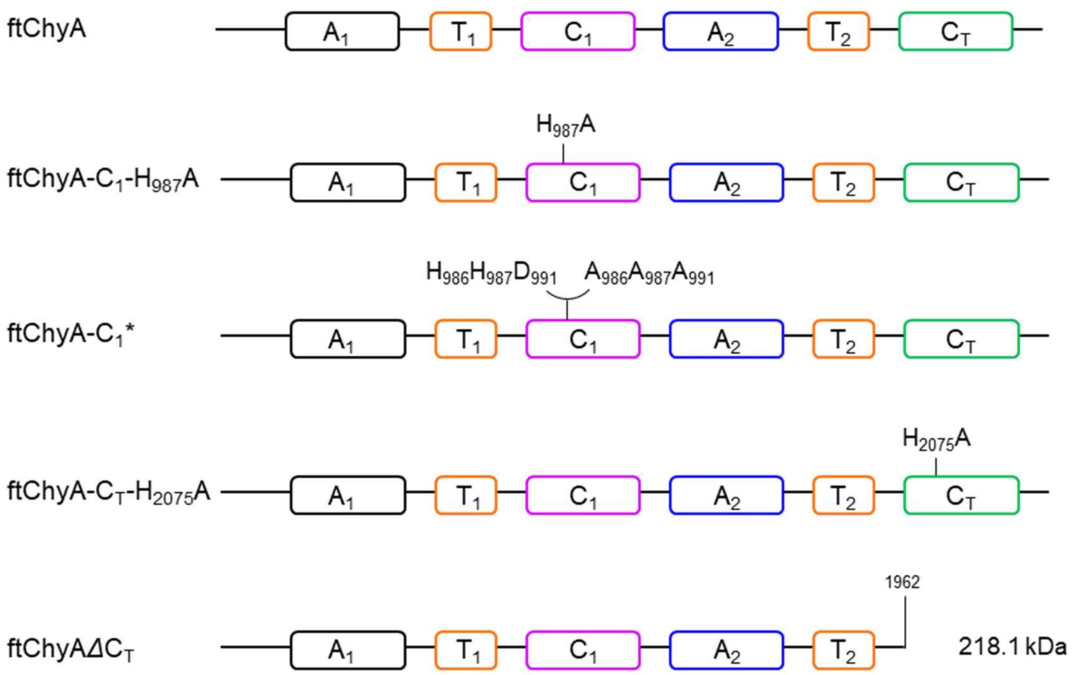

Supplementary Fig. 8 Schematic diagrams of ftChyA and its mutants.

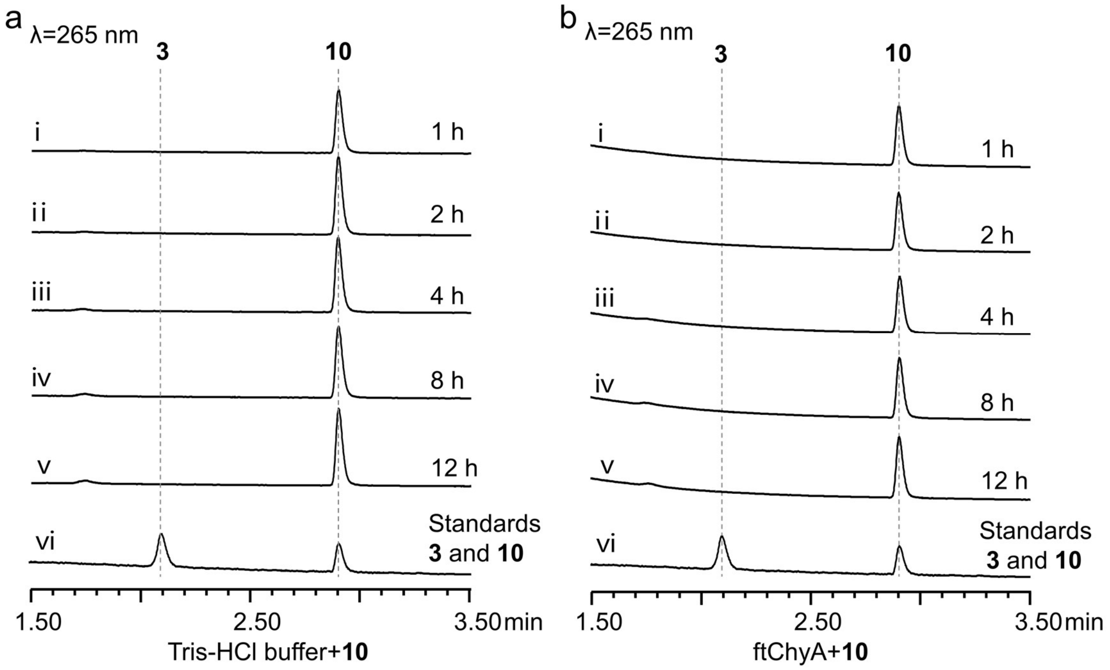

Supplementary Fig. 9 Time-course assays of the stability of **10**. (**a**) in Tris-HCl buffer (pH 7.5), (**b**) with ftChyA addition.

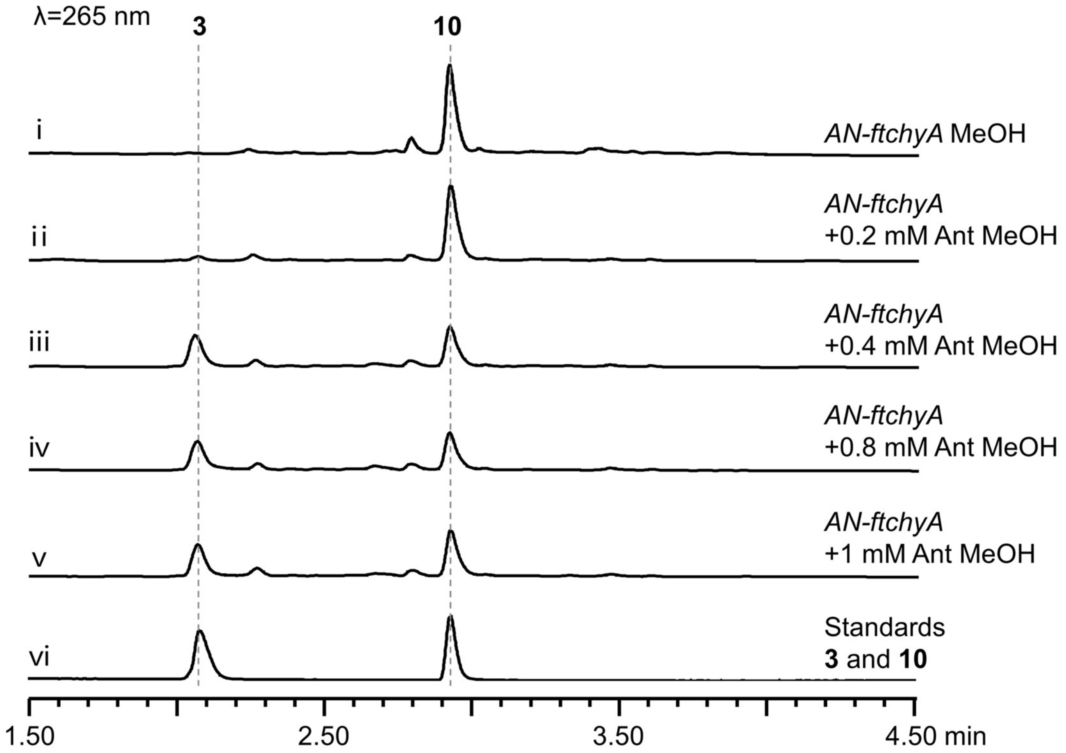

Supplementary Fig.10 LC-MS analyses of the *AN-ftchyA* with feeding different concentrations of Ant.

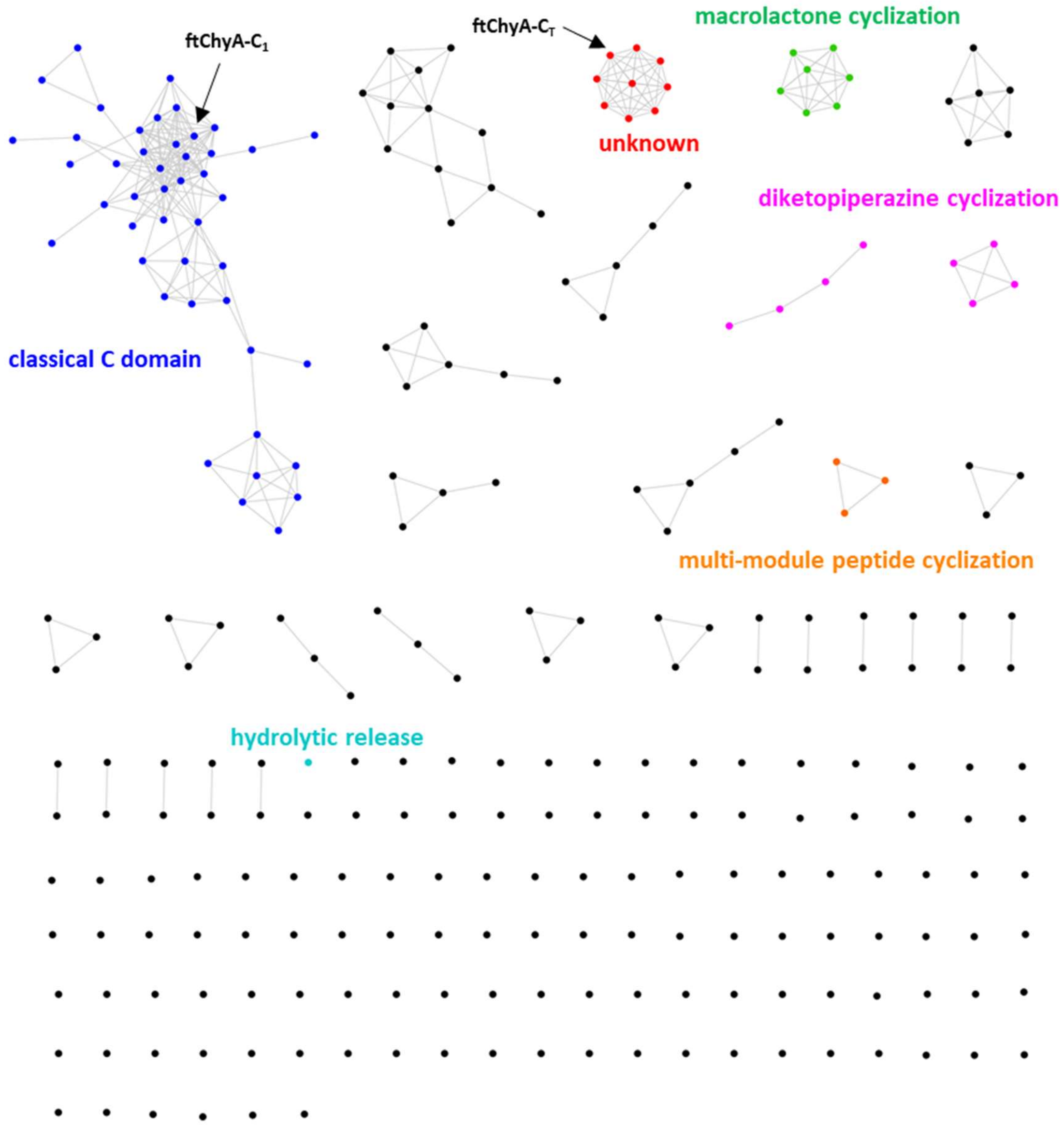

Supplementary Fig. 11 Sequence clustering analysis of the ftChyA-C$_1$ and ftChyA-C$_T$ with other fungal C domains. SSN of 273 fungal C domains homologs generated by Cytoscape (v3.8.2). Each node in the network represents a protein sequence and the alignment score is 45.

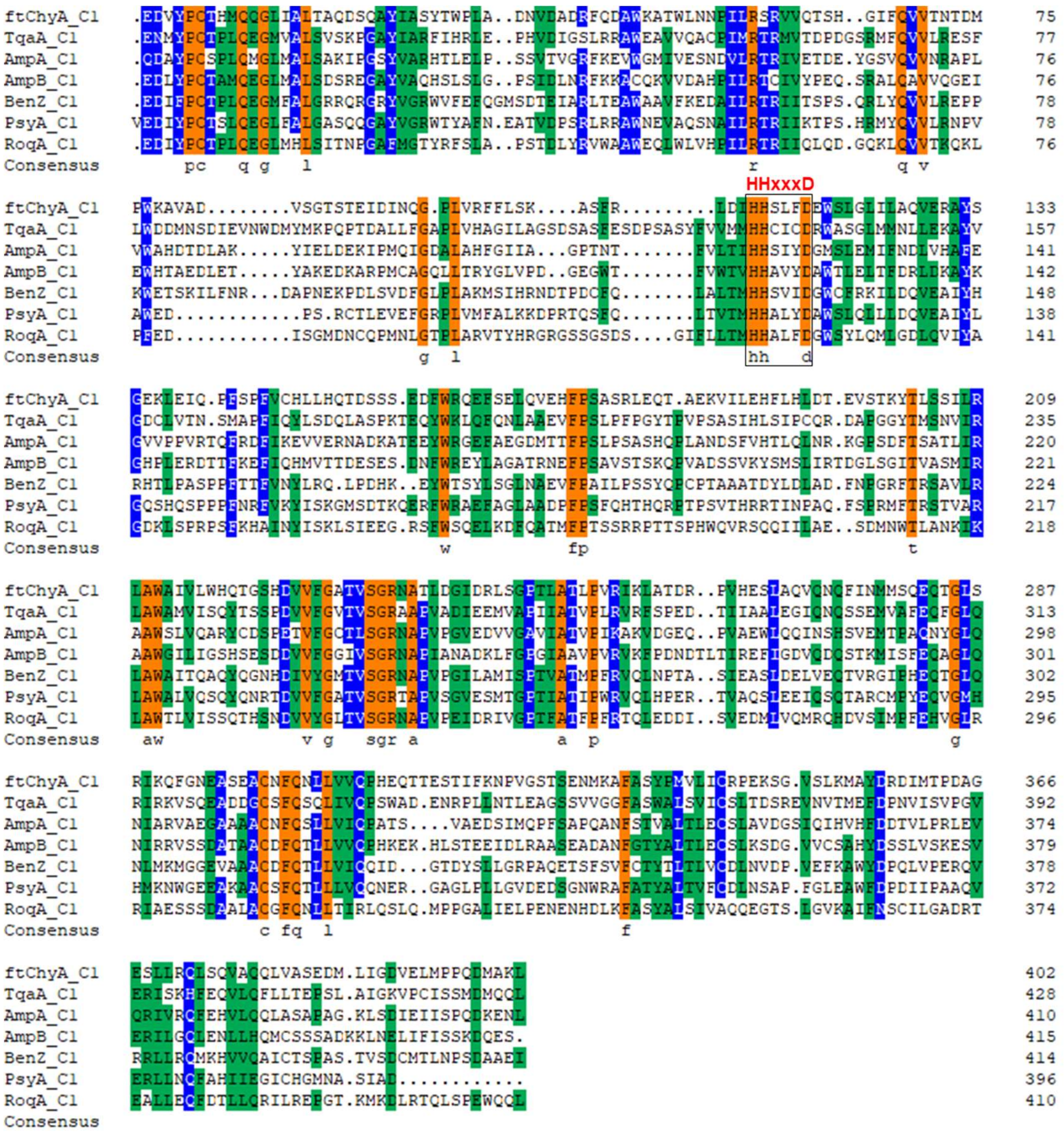

Supplementary Fig. 12 Sequences alignments of ftChyA-C_1 with other identified fungal NRPS C domains. The black box shows the conserved HHxxxD motif in ftChyA-C_1.

TqaA: ADY16697.1 (https://www.ncbi.nlm.nih.gov/protein/ADY16697.1),

AmpA: A0A1W6BT53.1 (https://www.ncbi.nlm.nih.gov/protein/A0A1W6BT53.1),

AmpB: A0A1W6BT46.1 (https://www.ncbi.nlm.nih.gov/protein/A0A1W6BT46.1),

BenZ: P9WEU9.1 (https://www.ncbi.nlm.nih.gov/protein/P9WEU9.1),

PsyA: AMQ36132.1 (https://www.ncbi.nlm.nih.gov/protein/AMQ36132.1),

RoqA: B6HJU6.1 (https://www.ncbi.nlm.nih.gov/protein/B6HJU6.1).

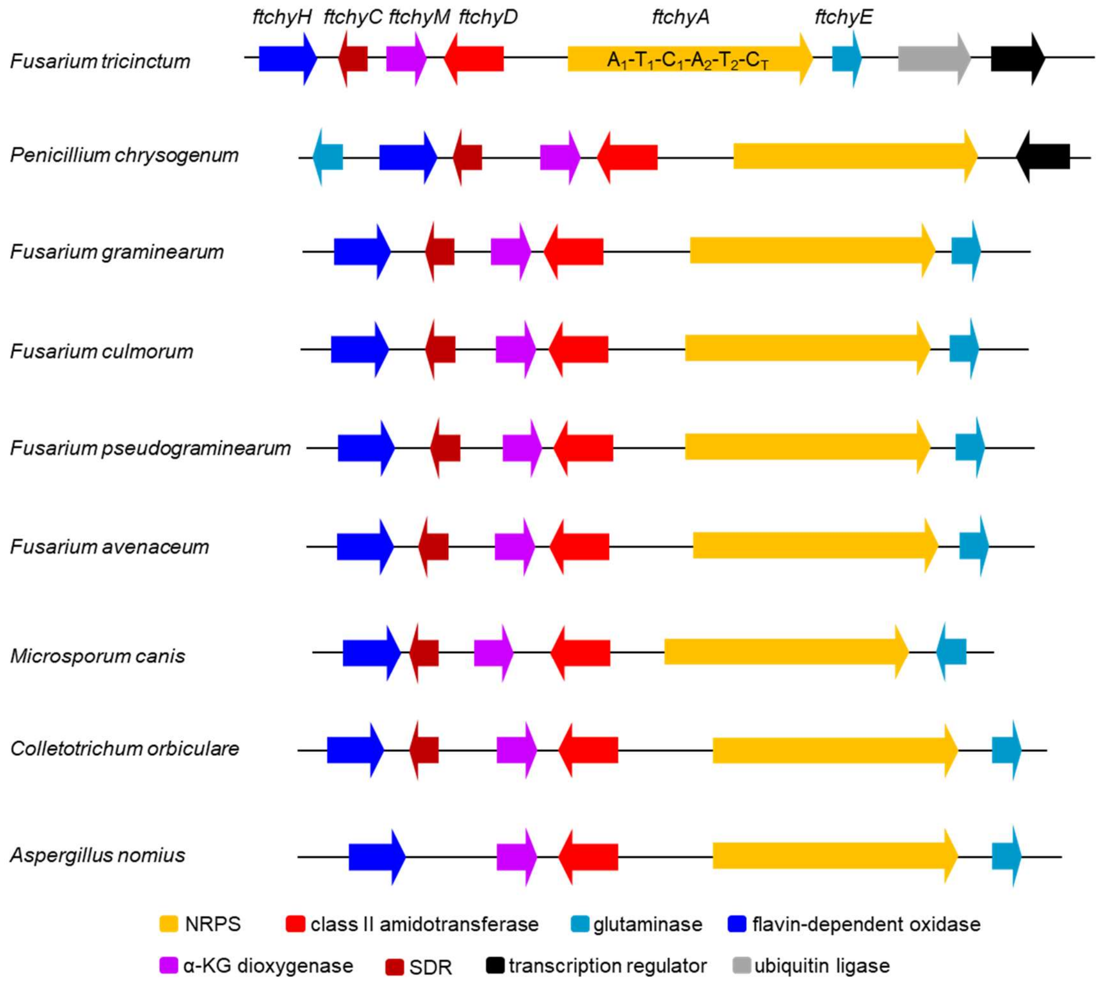

Supplementary Fig. 13 The genome-mined homologous *chy* clusters from different fungi.

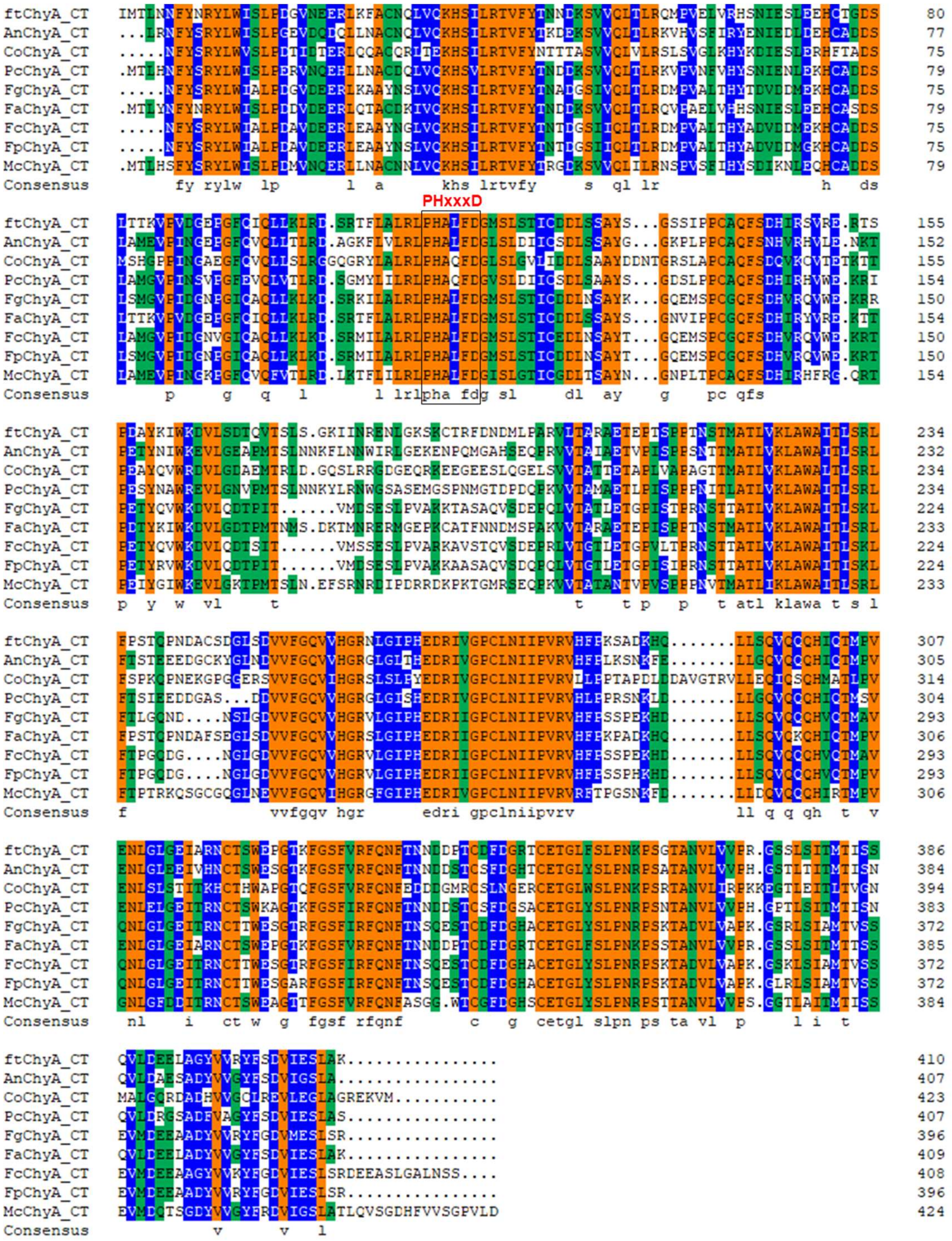

Supplementary Fig. 14 Sequence alignments of ftChyA-C_T with homologous domains from other *chy* clusters. The black box shows the conserved PHxxxD motif in ftChyA-C_T.

AnChyA: XP_015403503.1 (https://www.ncbi.nlm.nih.gov/protein/XP_015403503.1),
CoChyA: TDZ15643.1 (https://www.ncbi.nlm.nih.gov/protein/TDZ15643.1),
PcChyA: B6HLP9.1 (https://www.ncbi.nlm.nih.gov/protein/B6HLP9.1),
FgChyA: I1S3K7.1 (https://www.ncbi.nlm.nih.gov/protein/I1S3K7.1),
FaChyA: KIL85360.1 (https://www.ncbi.nlm.nih.gov/protein/KIL85360.1),
FcChyA: QPC63060.1 (https://www.ncbi.nlm.nih.gov/protein/QPC63060.1),

FpChyA: XP_009262411.1 (https://www.ncbi.nlm.nih.gov/protein/XP_009262411.1),
McChyA: XP_002842771.1 (https://www.ncbi.nlm.nih.gov/protein/XP_002842771.1).

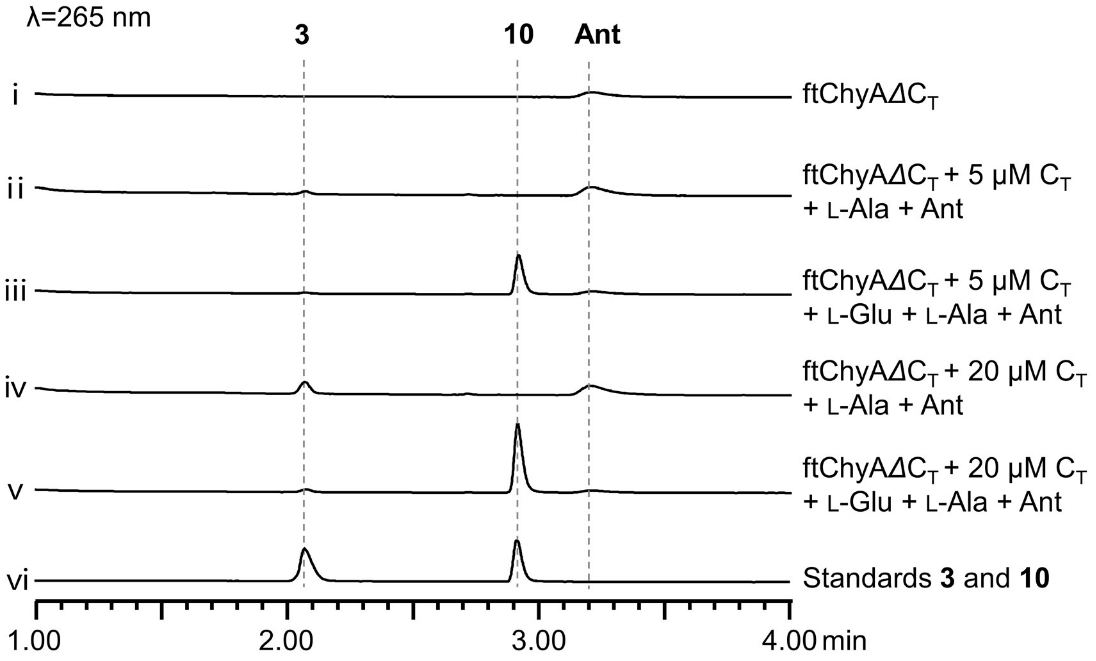

Supplementary Fig. 15 The ftChyA-C$_T$ domain is essential for catalysing the formation of **10** and **3**.

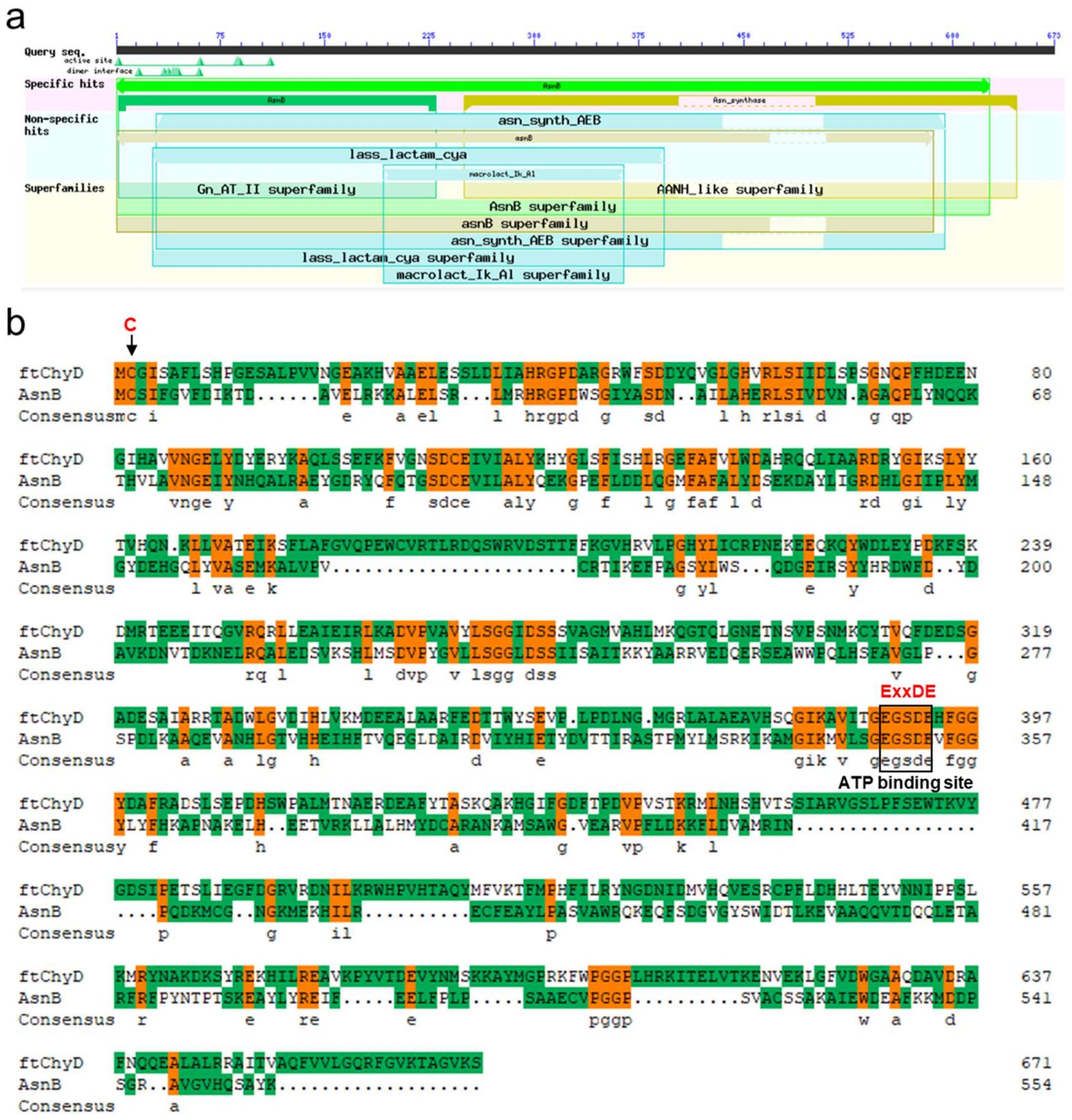

Supplementary Fig. 16 Bioinformatic analysis of ftChyD. (**a**) Conserved domain analysis of ftChyD. (**b**) Sequence alignments of ftChyD with asparagine synthetase B (AsnB). The black arrow shows the Cys for hydrolysis. The black box shows the conserved ExxDE motif for ATP binding site. AsnB: AJE54988.1 (https://www.ncbi.nlm.nih.gov/protein/AJE54988.1).

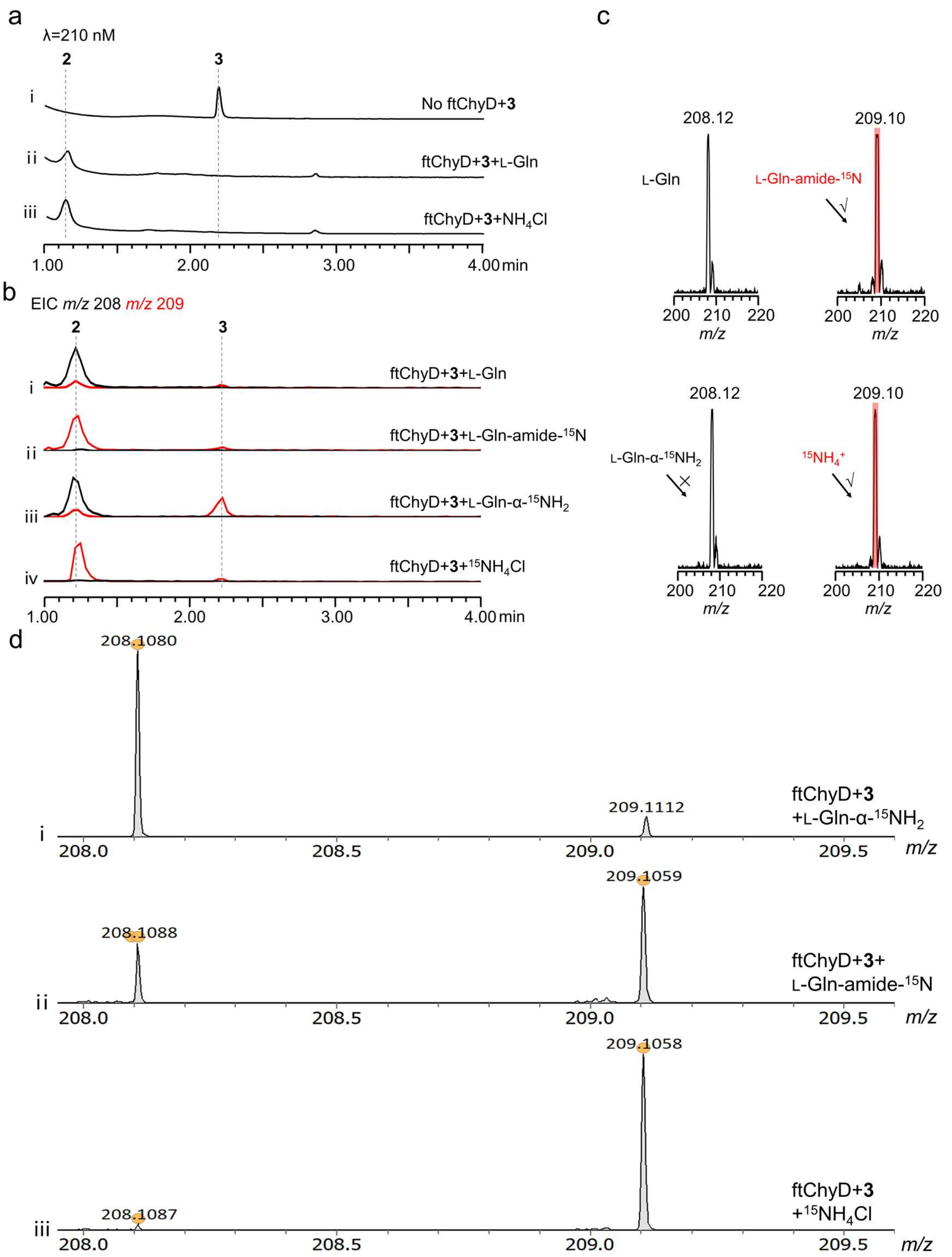

Supplementary Fig. 17 Biochemical confirmation of the amidation of ftChyD towards **3**. (**a**) ftChyD uses NH4Cl or L-Gln-amide to catalyse the amidation of **3** to form **2**. (**b**) LC-MS analysis of the incorporation of ^{15}N into **2**. The EICs were extracted at *m/z* 209 $[M + H]^+$ for **3** and ^{15}N-labelled **2**, and *m/z* 208 $[M + H]^+$ for **2**. (**c**) Mass of **2** and ^{15}N-labelled **2**. (**d**) HRMS analysis of the incorporation of ^{15}N into **2**.

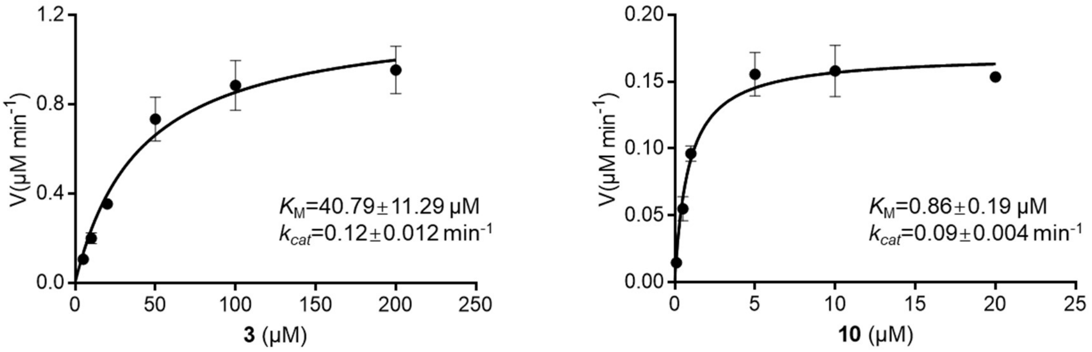

Supplementary Fig. 18 The kinetic parameters of ftChyD toward **3** and **10**. These values were calculated by analyzing production of **2** and **11** by LC-MS analysis. This measurement was conducted in four times. K_M and k_{cat} values represent the mean ± SEM.

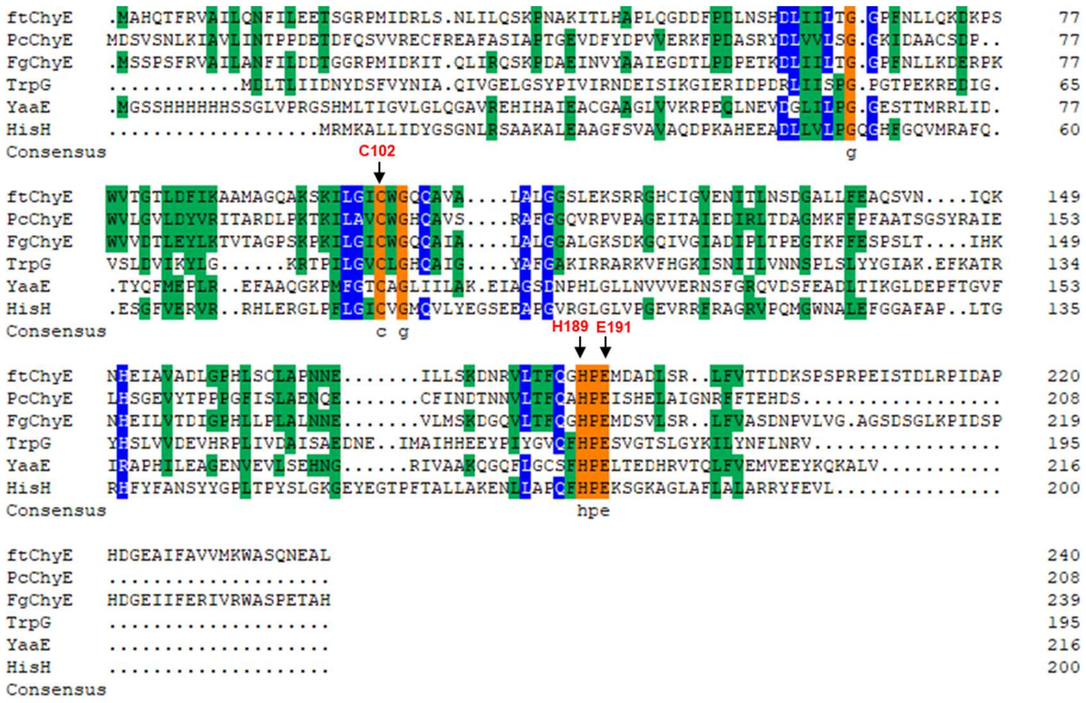

Supplementary Fig. 19 Sequence alignments of ftChyE with homologous enzymes and other identified glutaminase subunits of class I glutamine amidotransferases. The black arrow shows the catalytic triad C-H-E in ftChyE. PcChyE from *Penicillium chrysogenum*.

FgChyE: XP_011325839.1 (https://www.ncbi.nlm.nih.gov/protein/XP_011325839.1),
TrpG: 1QDL_B (https://www.ncbi.nlm.nih.gov/protein/1QDL_B),
YaaE: 1R9G_A (https://www.ncbi.nlm.nih.gov/protein/1R9G_A),
HisH: 1KA9_H (https://www.ncbi.nlm.nih.gov/protein/1KA9_H).

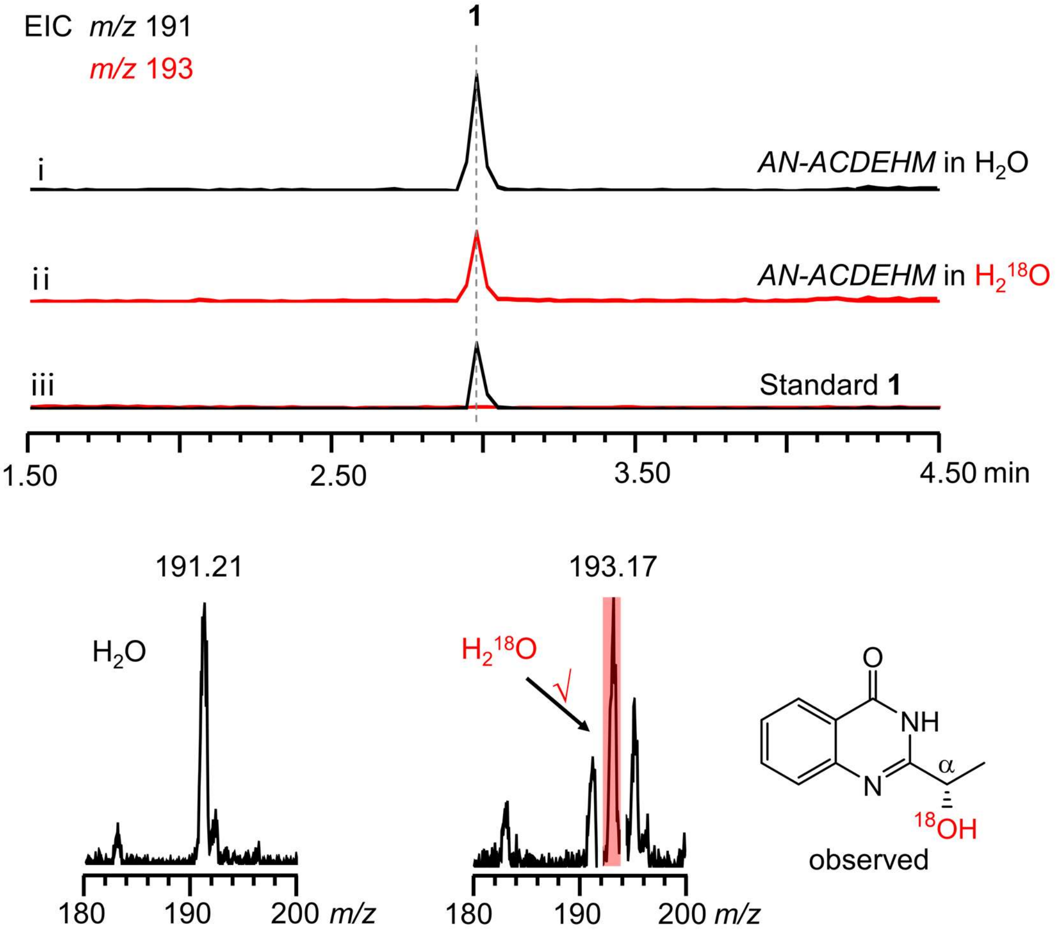

Supplementary Fig. 20 LC-MS analysis of the *AN-ftchyACDEHM* in $H_2{}^{18}O$-medium showing the oxygen atom of the α-OH group of **1** could be from water. The extracted ion chromatograms (EICs) were extracted at *m/z* 191[M + H]$^+$ for **1** and *m/z* 193 [M + H]$^+$ for ^{18}O-labelled **1**.

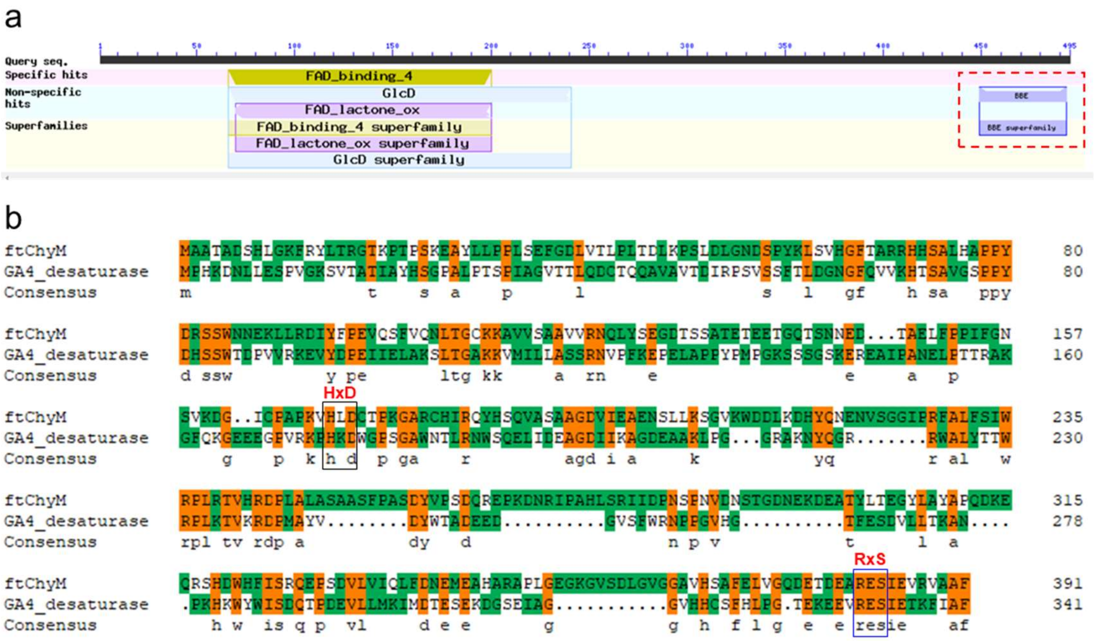

Supplementary Fig. 21 Conserved domain analysis of ftChyH and ftChyM. (**a**) ftChyH is a flavin-dependent oxidase that contains a berberine bridge enzyme (BBE) conserved domain. The red box shows that the berberine bridge enzyme (BBE) conserved domain is existed in ftChyH. (**b**) ftChyM is an α-KGD contains HxD motif and RxS motif. GA4 desaturase: CAD10289.1 (https://www.ncbi.nlm.nih.gov/protein/CAD10289.1). The black box shows the HxD motif for binding Fe^{2+}. The blue box shows the RxS motif for binding α-ketoglutarate.

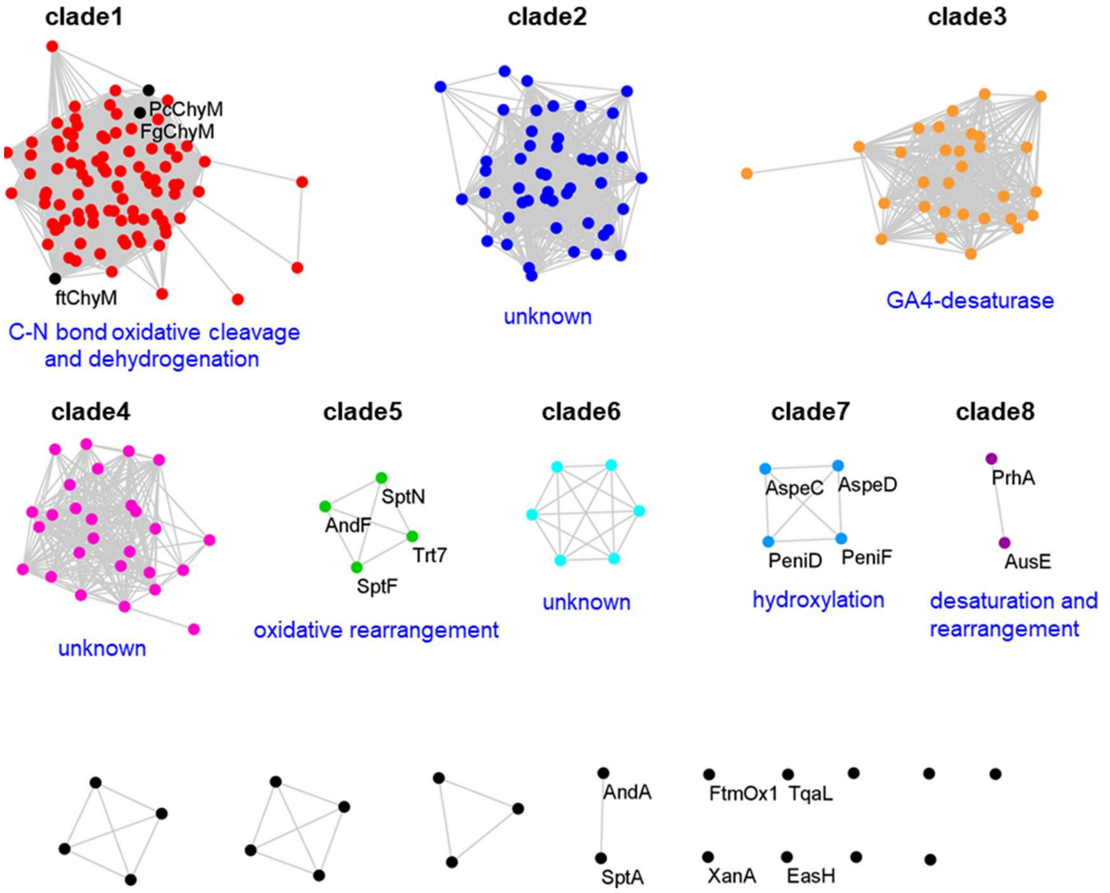

Supplementary Fig. 22 Sequence clustering analysis of ftChyM with other fungal α-ketoglutarate dependent dioxygenases. SSN of 239 fungal α-ketoglutarate dependent dioxygenases homologs generated by Cytoscape (v3.8.2). Each node in the network represents a protein sequence and the alignment score is 60.

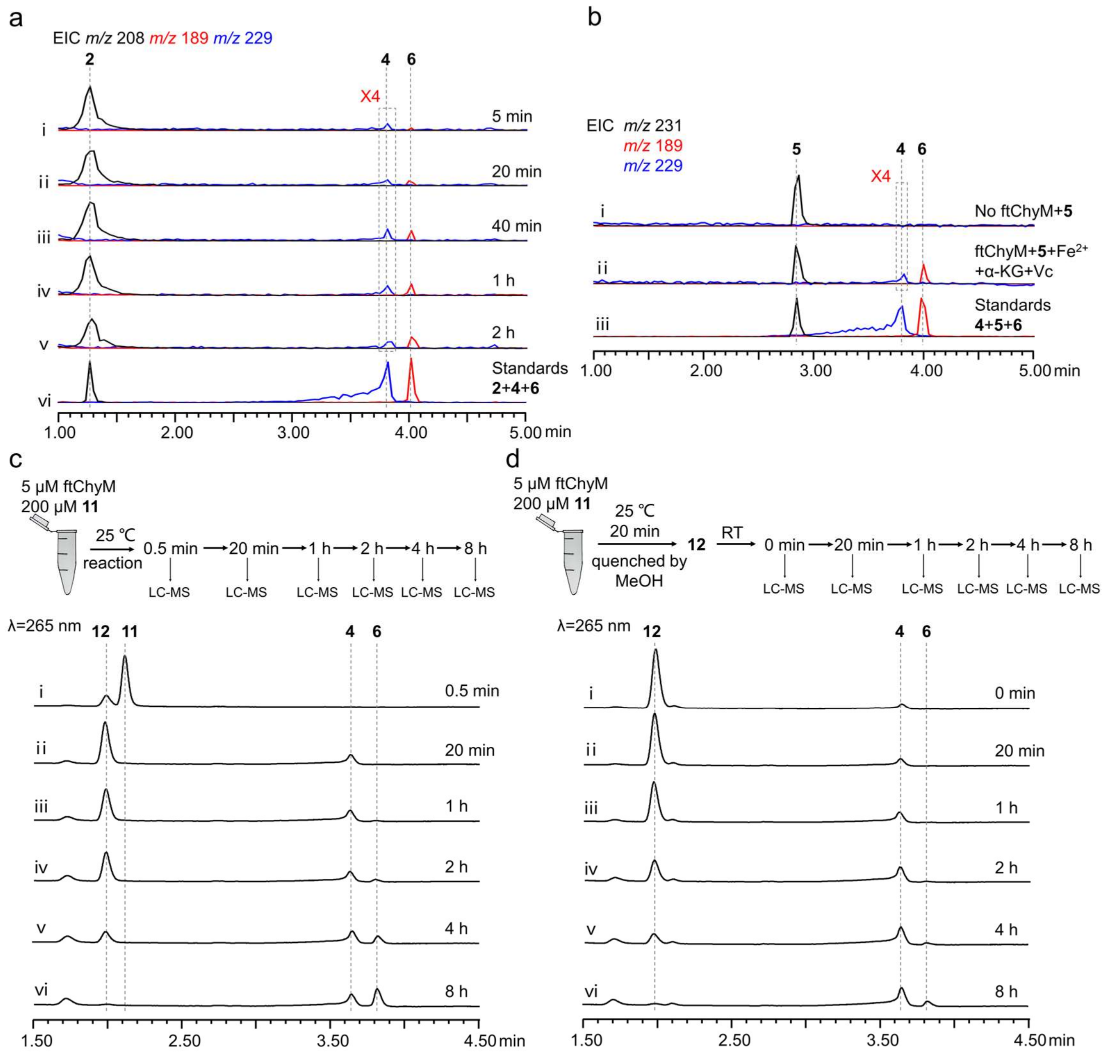

Supplementary Fig. 23 The biochemical assays of ftChyM with different substrates. (**a**) Time course assays of ftChyM with **2**. (**b**) ftChyM catalyse dehydrogenation of **5** to form **4**. (**c**) Time course assays of ftChyM with **11**. (**d**) Time course assays of *in situ* synthesized **12** spontaneously converted to **4** and **6** in room temperature.

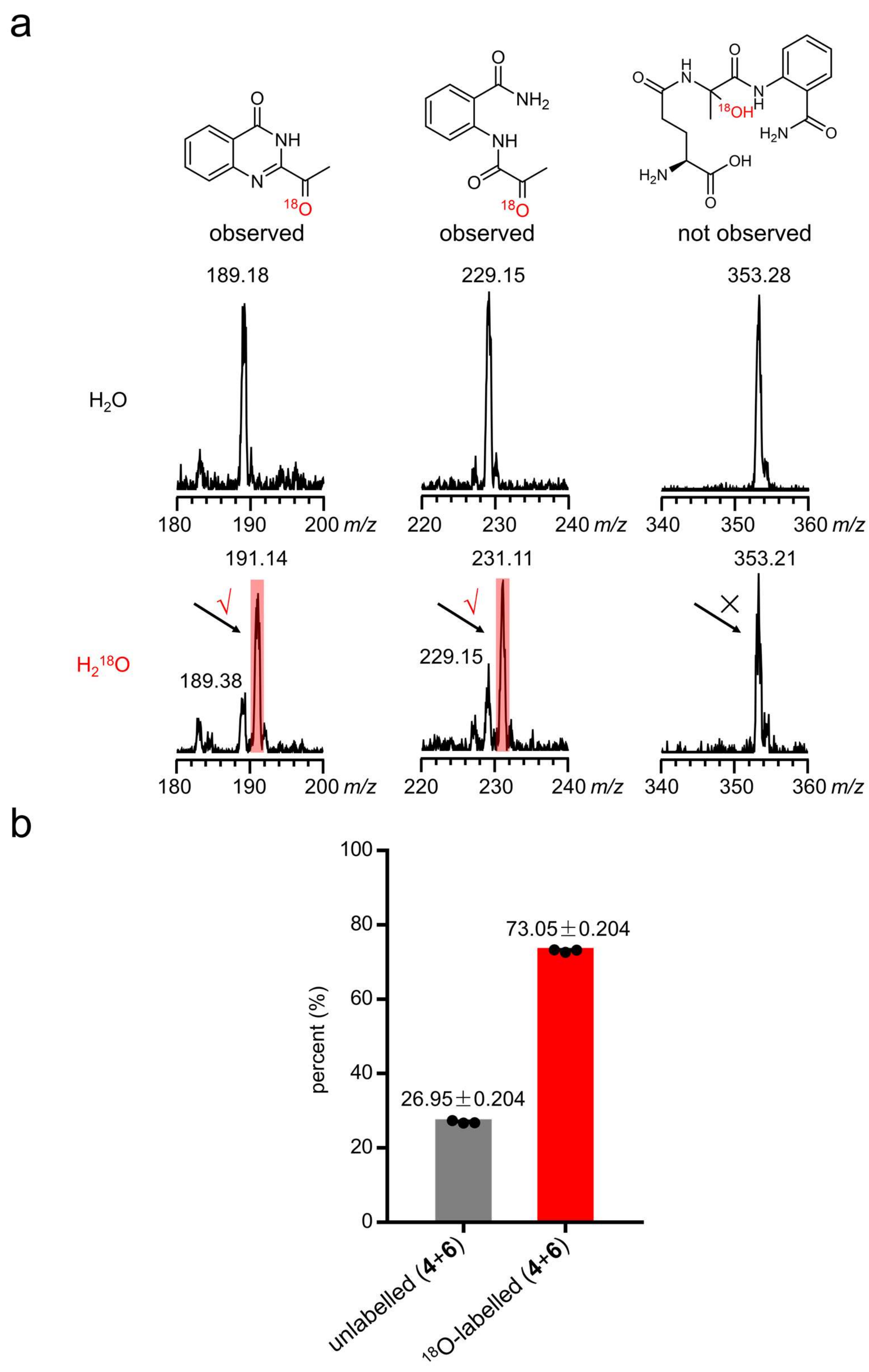

Supplementary Fig. 24 The biochemical assays of ftChyM with **11** in ~80% $H_2{}^{18}O$-Tris-HCl buffer. (**a**) LC-MS analysis of the ftChyM with **11** in $H_2{}^{18}O$ show that ^{18}O was incorporated into **4** and **6**. (**b**) Analysis of unlabeled and ^{18}O-labelled **4** and **6** (in sum). Data was shown as mean ± SEM for 3 independent experiments.

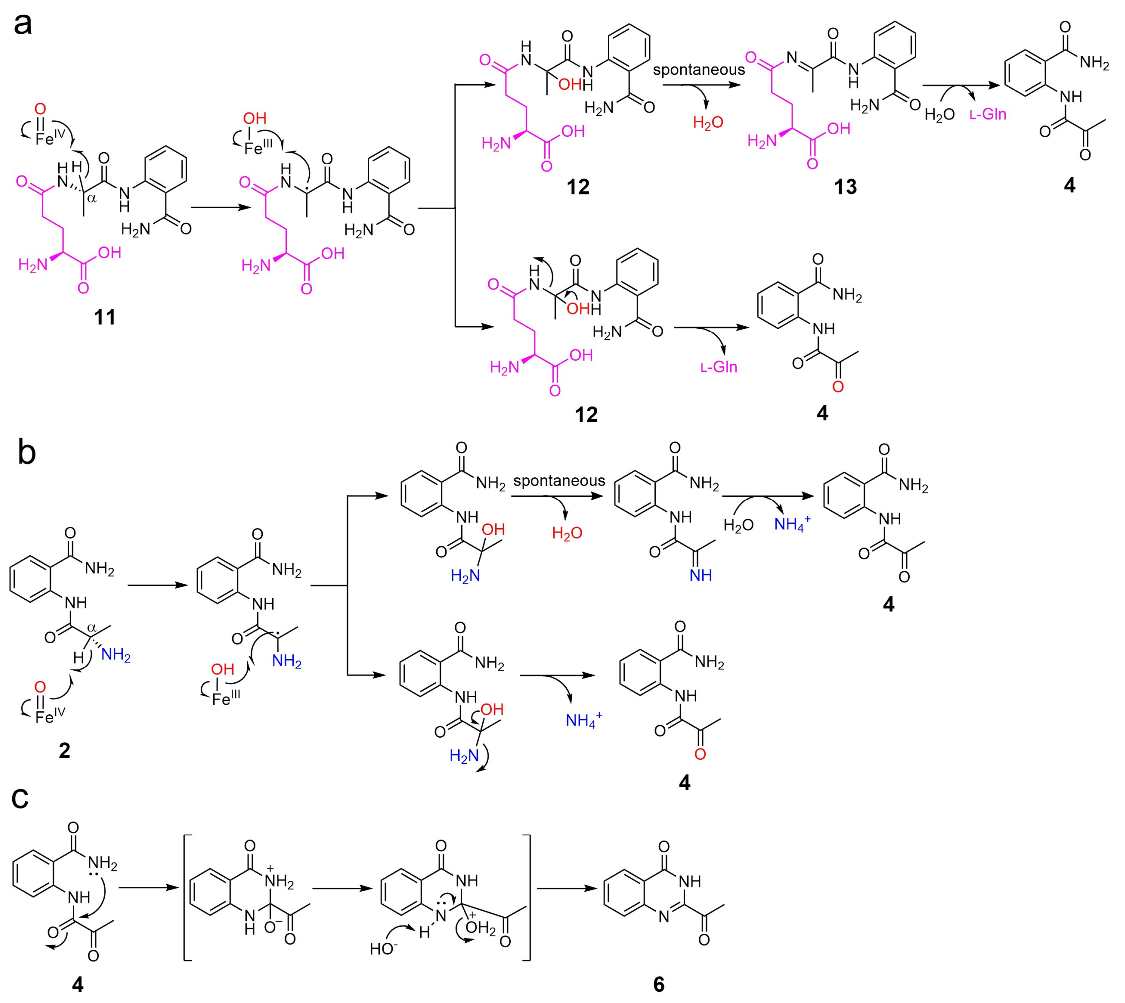

Supplementary Fig. 25 The proposed mechanism of the (**a**) ftChyM-catalyzed C-N bond oxidative cleavage **11** to form **4**; (**b**) the ftChyM-catalyzed oxidative deamination of **2** to form **4**; (**c**) the alkaline-induced spontaneous C-2-N-3 bond closure of **4** to form **6**.

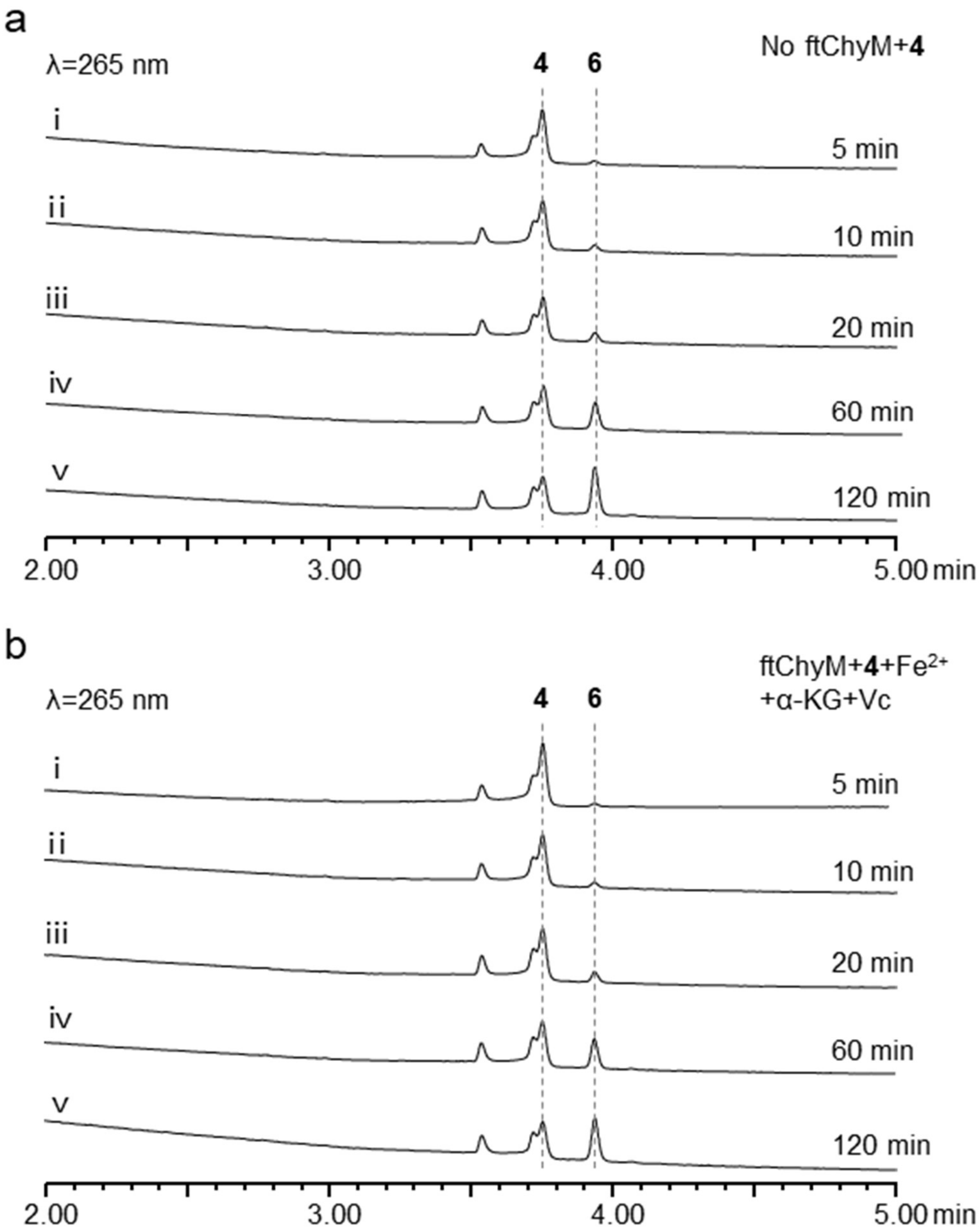

Supplementary Fig. 26 Time-course assays of the spontaneous conversion of **4** to **6**. (**a**) Tris-HCl buffer (pH 7.5); (**b**) with ftChyM addition.

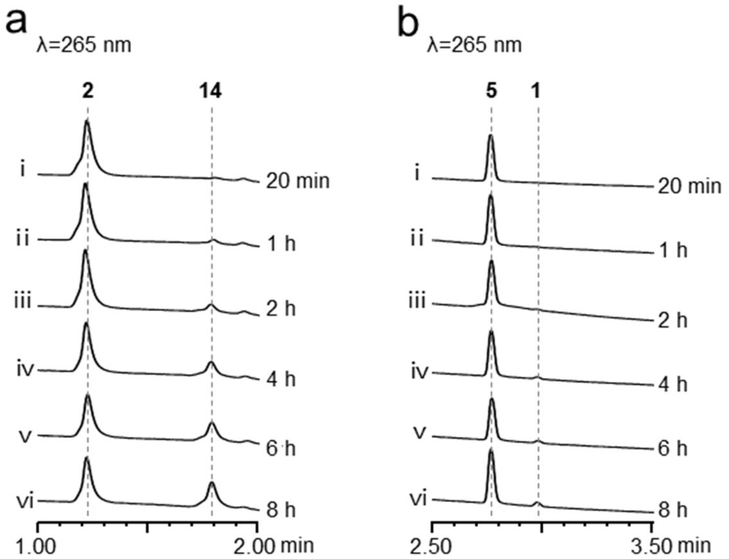

Supplementary Fig. 27 Spontaneous cyclization of compound **2**, **5** and **4** in Tris-HCl buffer (pH 7.5). (**a**) cyclization of **2** to form **14**; (**b**) cyclization of **5** to form **1**; (**c**) cyclization of **4** to form **6**.

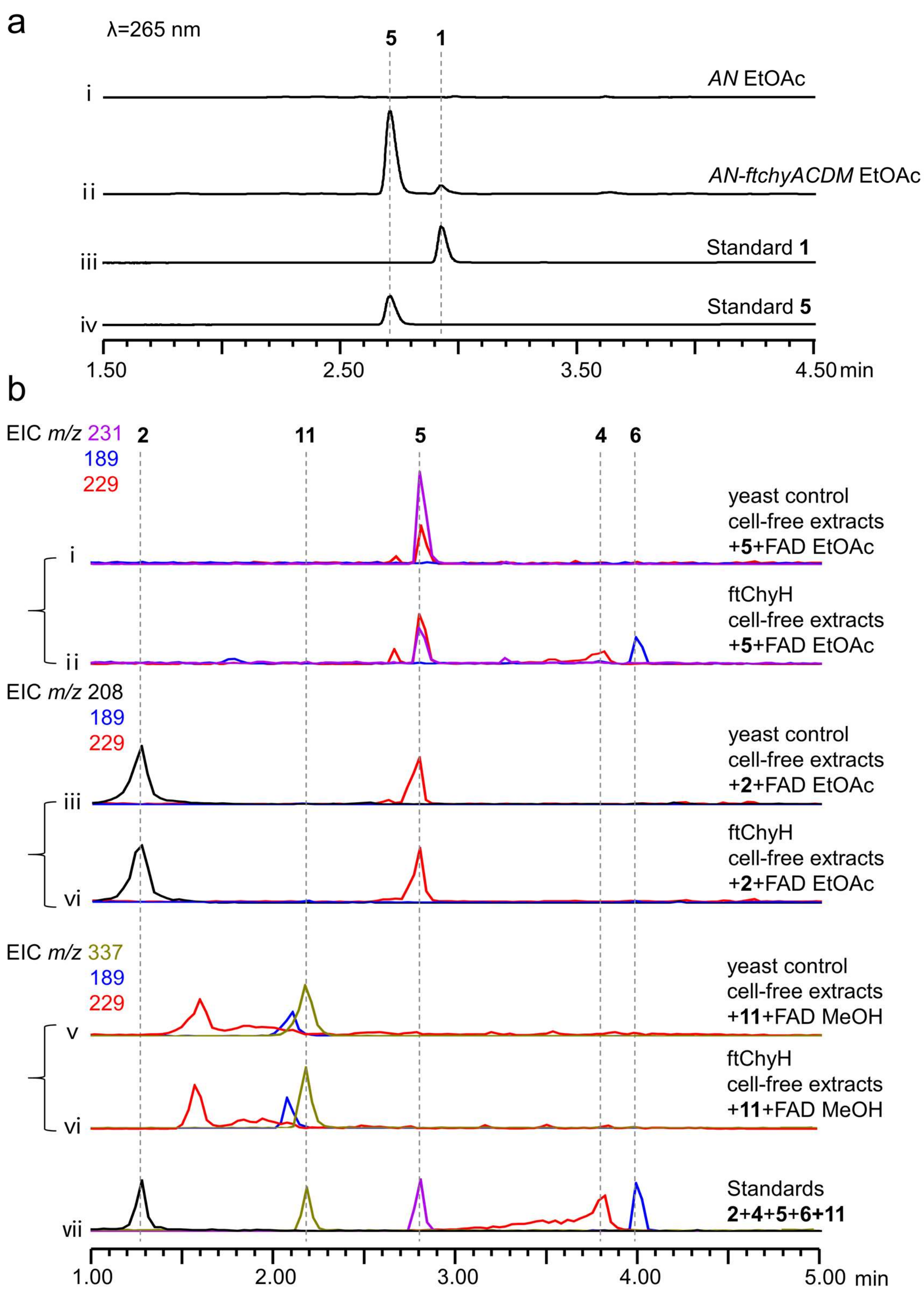

Supplementary Fig. 28 Biochemical confirmation of the function of ftChyH. (**a**) LC-MS analyses of the *AN-ftchyACDM* showing elimination of *ftchyH* accumulates **5**. (**b**) Biochemical assay of ftChyH cell-free extracts with **5**, **2** and **11**, respectively. The extracted ion chromatograms (EICs) were extracted at *m/z* 208 $[M + H]^+$ for **2**, *m/z* 229 $[M + Na]^+$ for **4**, *m/z* 231 $[M + Na]^+$ for **5**, *m/z* 189 $[M + H]^+$ for **6**, *m/z* 337 $[M + H]^+$ for **11**.

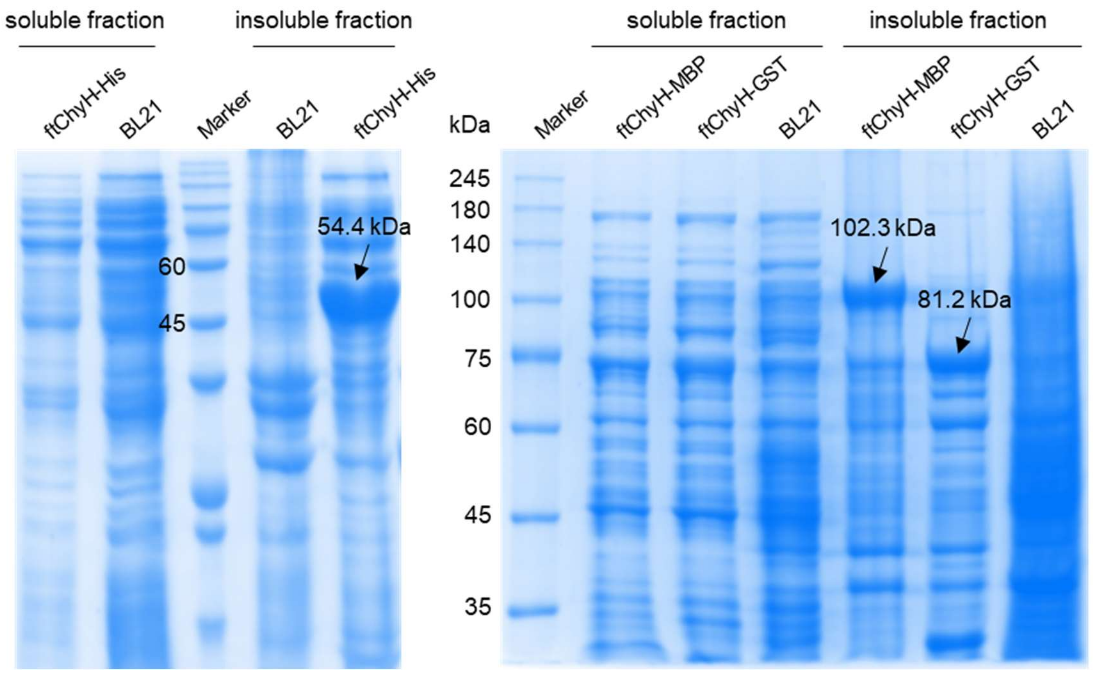

Supplementary Fig. 29 SDS-PAGE analyses of the expression of ftChyH-His, ftChyH-MBP and ftChyH-GST in *E. coli* BL21, respectively. All experiments were repeated independently more than three times with similar results.

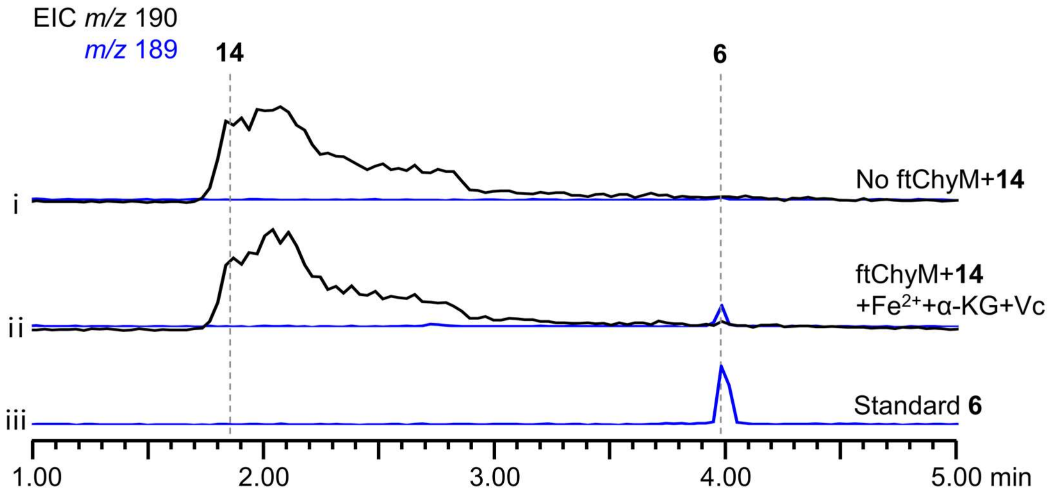

Supplementary Fig. 30 Biochemical assay of ftChyM with **14** *in vitro*. The extracted ion chromatograms (EICs) were extracted at *m/z* 190 $[M + H]^+$ for **14** and *m/z* 189 $[M + H]^+$ for **6**.

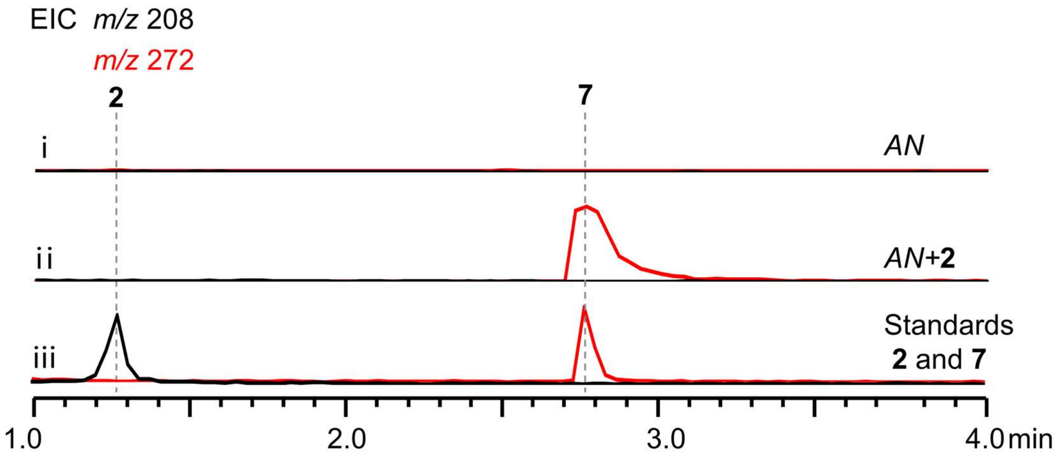

Supplementary Fig. 31 LC-MS analyses of the *A. nidulans* with feeding **2** showing that **2** was converted to **7** by an unknown acetyltransferase of *A. nidulans.* The extracted ion chromatograms (EICs) were extracted at *m/z* 208 $[M + H]^+$ for **2** and *m/z* 272 $[M + Na]^+$ for **7**.

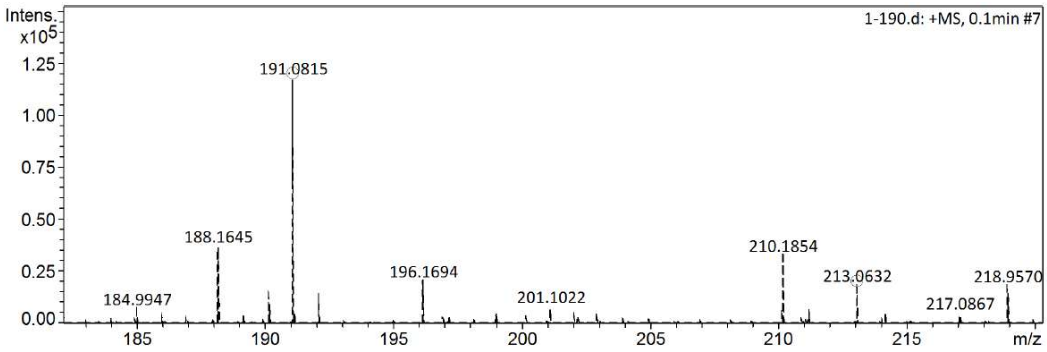

Meas. m/z	#	Ion Formula	Sum Formula	m/z	Adduct	err [ppm]	z	mSigma	Score	rdb	N-Rule	e^- Conf
191.0815	1	C10H11N2O2	C10H10N2O2	191.0815	M+H	0.1	1+	7.2	100.00	7.0	ok	even
213.0632	1	C10H10N2NaO2		213.0634	M+Na	1.2	1+	n.a.	100.00	7.0	ok	even

Supplementary Fig. 32 HRMS spectrum (positive ionization) of compound **1**.

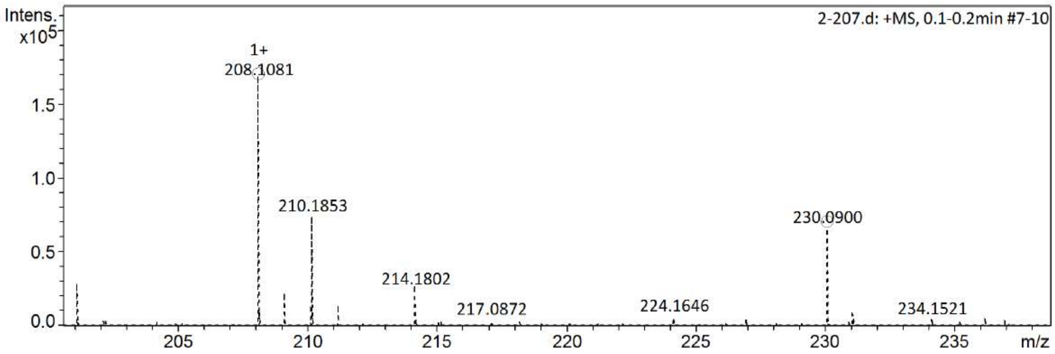

Meas. m/z	#	Ion Formula	Sum Formula	m/z	Adduct	err [ppm]	z	mSigma	Score	rdb	N-Rule	e^- Conf
208.1081	1	C10H14N3O2	C10H13N3O2	208.1081	M+H	-0.3	1+	3.9	100.00	6.0	ok	even
230.0900	1	C10H13N3NaO2		230.0900	M+Na	0.0	1+	3.1	100.00	6.0	ok	even

Supplementary Fig. 33 HRMS spectrum (positive ionization) of compound **2**.

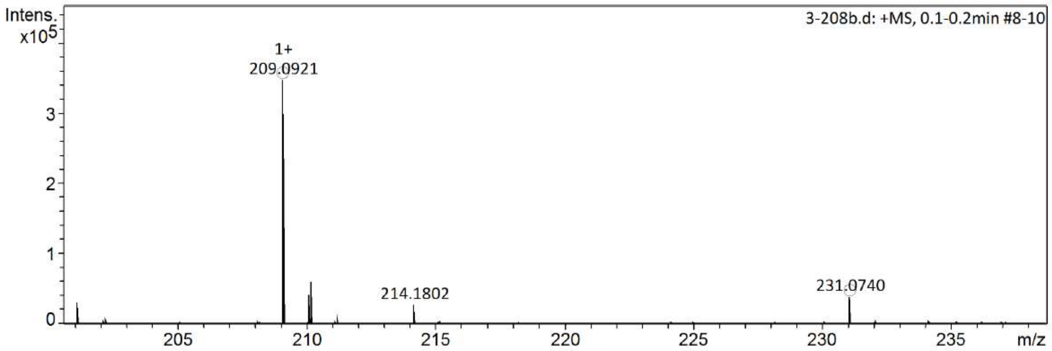

Meas. m/z	#	Ion Formula	Sum Formula	m/z	Adduct	err [ppm]	z	mSigma	Score	rdb	N-Rule	e⁻ Conf
209.0921	1	C10H13N2O3	C10H12N2O3	209.0921	M+H	-0.4	1+	0.9	100.00	6.0	ok	even
231.0740	1	C10H12N2NaO3		231.0740	M+Na	-0.2	1+	7.3	100.00	6.0	ok	even

Supplementary Fig. 34 HRMS spectrum (positive ionization) of compound **3**.

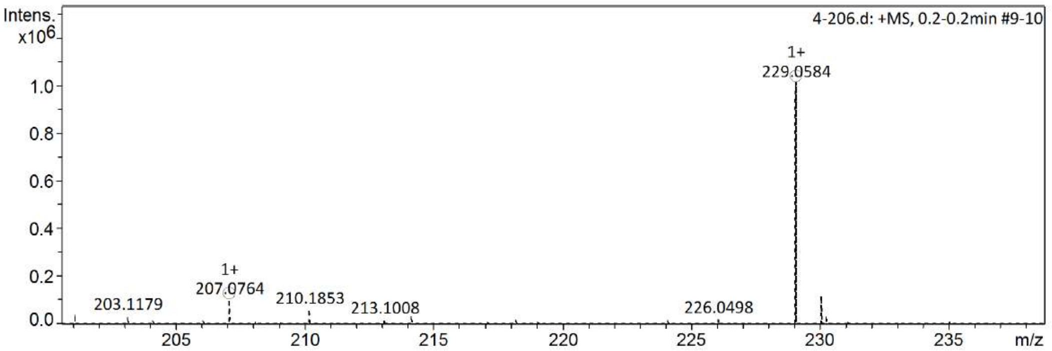

Meas. m/z	#	Ion Formula	Sum Formula	m/z	Adduct	err [ppm]	z	mSigma	Score	rdb	N-Rule	e⁻ Conf
207.0764	1	C10H11N2O3	C10H10N2O3	207.0764	M+H	0.2	1+	21.5	100.00	7.0	ok	even
229.0584	1	C10H10N2NaO3		229.0584	M+Na	0.0	1+	2.3	100.00	7.0	ok	even

Supplementary Fig. 35 HRMS spectrum (positive ionization) of compound **4**.

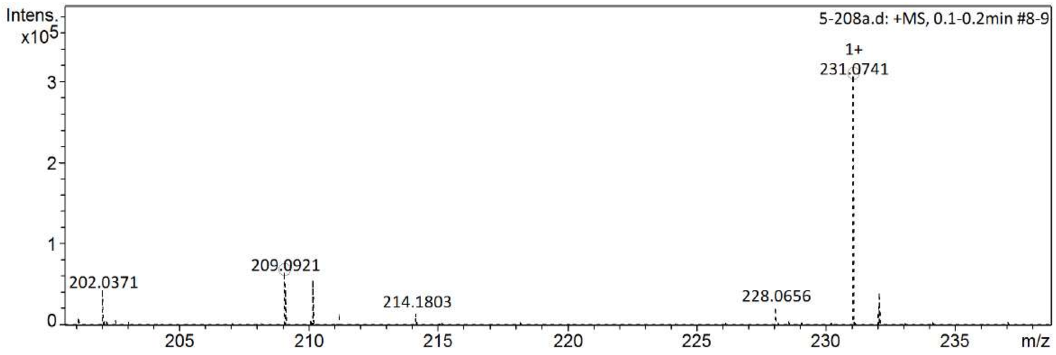

Meas. m/z	#	Ion Formula	Sum Formula	m/z	Adduct	err [ppm]	z	mSigma	Score	rdb	N-Rule	e⁻ Conf
209.0921	1	C10H13N2O3	C10H12N2O3	209.0921	M+H	-0.3	1+	1.3	100.00	6.0	ok	even
231.0741	1	C10H12N2NaO3		231.0740	M+Na	-0.3	1+	2.2	100.00	6.0	ok	even

Supplementary Fig. 36 HRMS spectrum (positive ionization) of compound **5**.

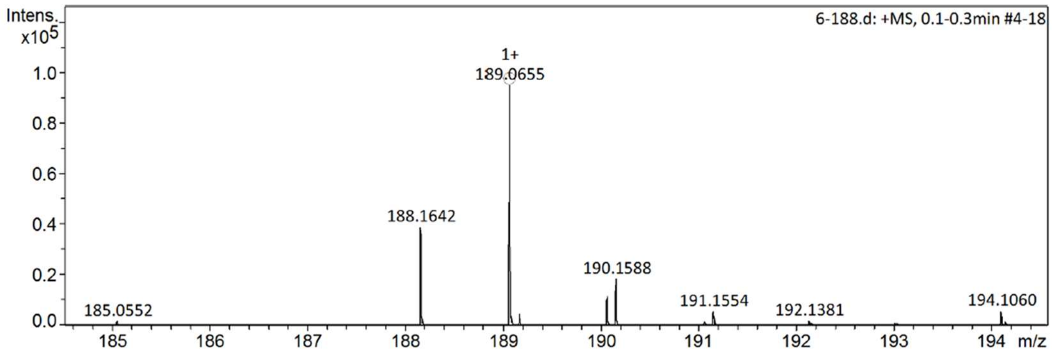

Meas. m/z	#	Ion Formula	Sum Formula	m/z	Adduct	err [ppm]	z	mSigma	Score	rdb	N-Rule	e⁻ Conf
189.0655	1	C10H9N2O2	C10H8N2O2	189.0659	M+H	2.0	1+	0.6	100.00	8.0	ok	even

Supplementary Fig. 37 HRMS spectrum (positive ionization) of compound **6**.

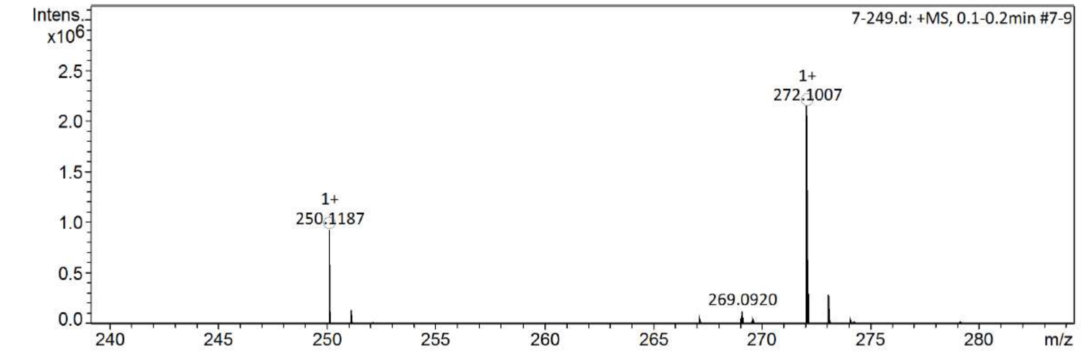

Meas. m/z	#	Ion Formula	Sum Formula	m/z	Adduct	err [ppm]	z	mSigma	Score	rdb	N-Rule	e⁻ Conf
250.1187	1	C12H16N3O3	C12H15N3O3	250.1186	M+H	-0.5	1+	0.4	100.00	7.0	ok	even
272.1007	1	C12H15N3NaO3		272.1006	M+Na	-0.3	1+	4.9	100.00	7.0	ok	even

Supplementary Fig. 38 HRMS spectrum (positive ionization) of compound **7**.

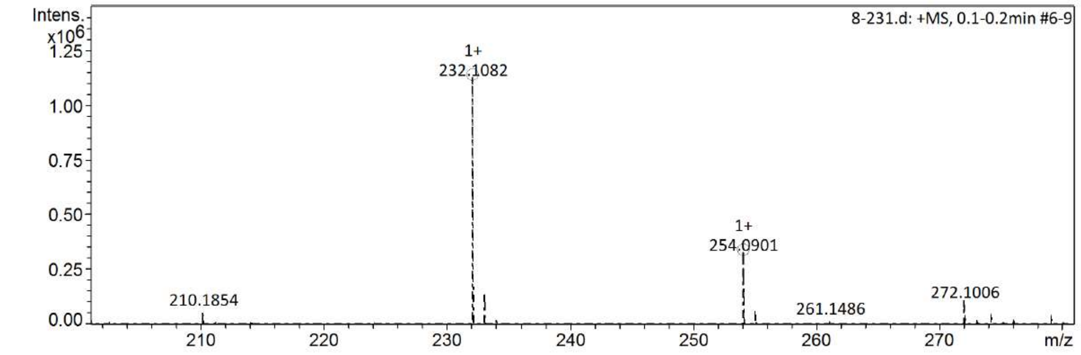

Meas. m/z	#	Ion Formula	Sum Formula	m/z	Adduct	err [ppm]	z	mSigma	Score	rdb	N-Rule	e⁻ Conf
232.1082	1	C12H14N3O2	C12H13N3O2	232.1081	M+H	-0.8	1+	5.1	100.00	8.0	ok	even
254.0901	1	C12H13N3NaO2		254.0900	M+Na	-0.5	1+	1.7	100.00	8.0	ok	even

Supplementary Fig. 39 HRMS spectrum (positive ionization) of compound **8**.

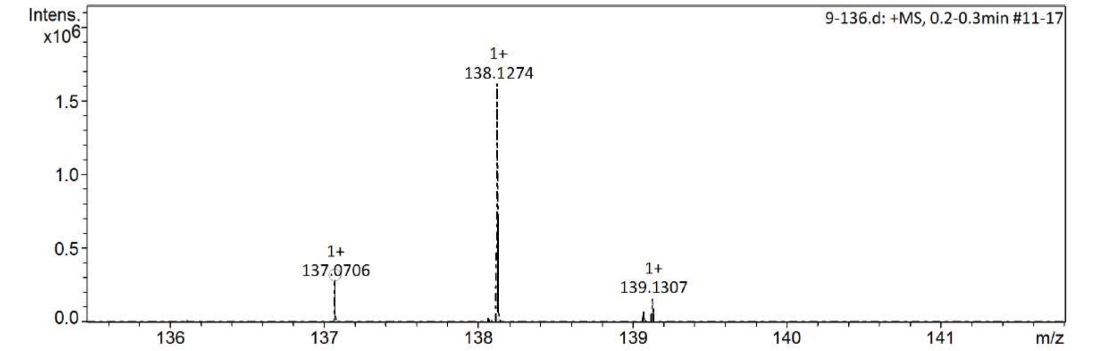

Meas. m/z	#	Ion Formula	Sum Formula	m/z	Adduct	err [ppm]	z	mSigma	Score	rdb	N-Rule	e^- Conf
137.0706	1	C7H9N2O	C7H8N2O	137.0709	M+H	2.8	1+	153.6	100.00	5.0	ok	even

Supplementary Fig. 40 HRMS spectrum (positive ionization) of compound **9**.

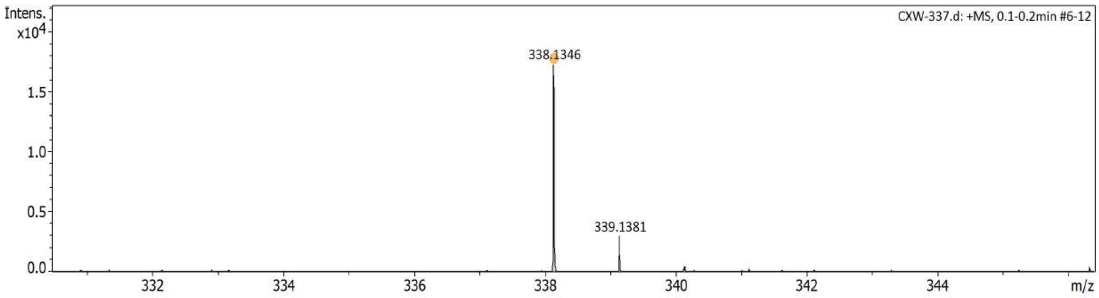

Meas. m/z	#	Ion Formula	Sum Formula	m/z	Adduct	err [ppm]	z	mSigma	Score	rdb	N-Rule	e^- Conf
338.1346	1	C15H20N3O6	C15H19N3O6	338.1347	M+H	0.2	1+	16.0	100.00	8.0	ok	even

Supplementary Fig. 41 HRMS spectrum (positive ionization) of compound **10**.

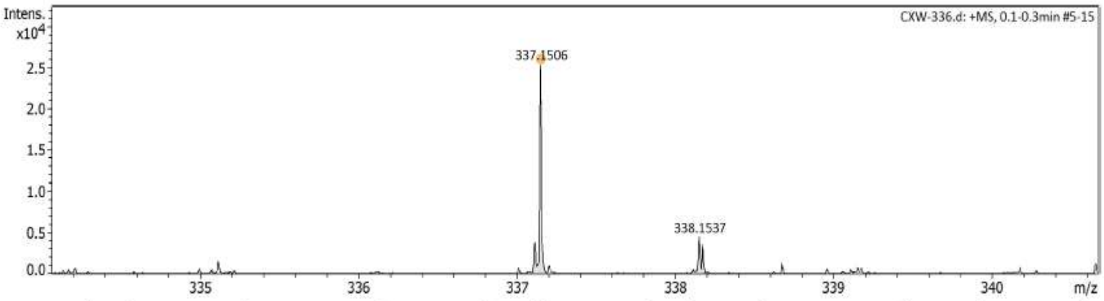

Meas. m/z	#	Ion Formula	Sum Formula	m/z	Adduct	err [ppm]	z	mSigma	Score	rdb	N-Rule	e⁻ Conf
337.1506	1	C15H21N4O5	C15H20N4O5	337.1506	M+H	0.2	1+	15.1	100.00	8.0	ok	even

Supplementary Fig. 42 HRMS spectrum (positive ionization) of compound **11**.

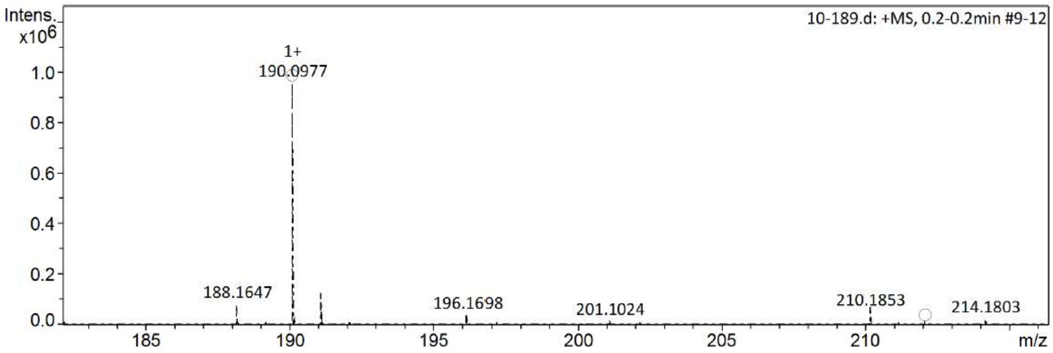

Meas. m/z	#	Ion Formula	Sum Formula	m/z	Adduct	err [ppm]	z	mSigma	Score	rdb	N-Rule	e⁻ Conf
190.0977	1	C10H12N3O	C10H11N3O	190.0975	M+H	-0.9	1+	3.9	100.00	7.0	ok	even
212.0795	1	C10H11N3NaO		212.0794	M+Na	-0.2	1+	5.1	100.00	7.0	ok	even

Supplementary Fig. 43 HRMS spectrum (positive ionization) of compound **14**.

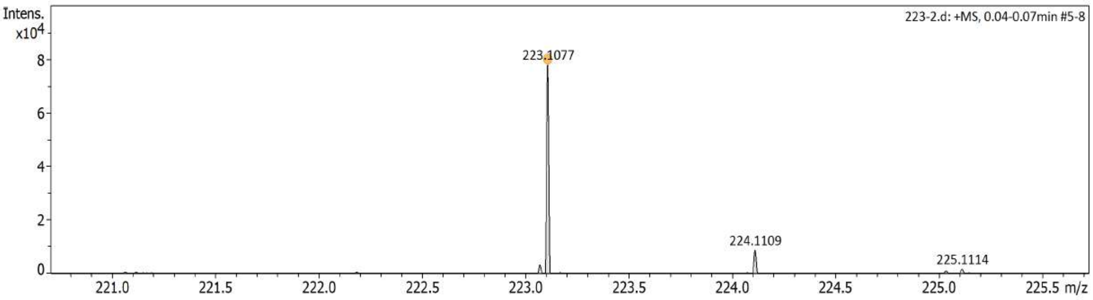

Meas. m/z	#	Ion Formula	Sum Formula	m/z	Adduct	err [ppm]	z	mSigma	Score	rdb	N-Rule	e⁻ Conf
223.1077	1	C11H15N2O3	C11H14N2O3	223.1077	M+H	0.1	1+	11.5	100.00	6.0	ok	even

Supplementary Fig. 44 HRMS spectrum (positive ionization) of compound Ant-Me-**3**.

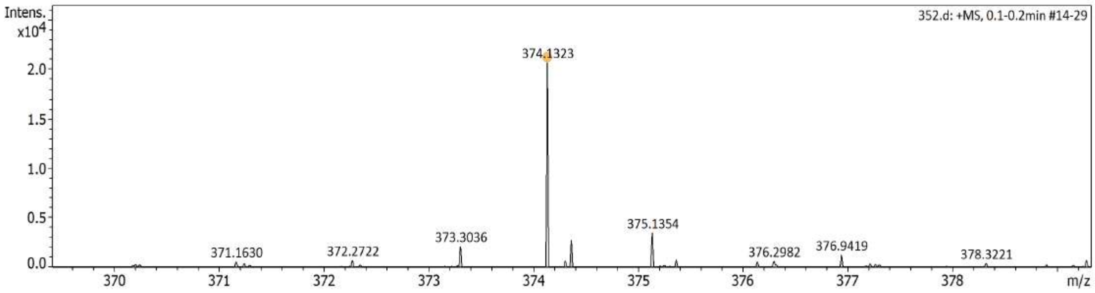

Meas. m/z	#	Ion Formula	Sum Formula	m/z	Adduct	err [ppm]	z	mSigma	Score	rdb	N-Rule	e⁻ Conf
374.1323	1	C16H21N3NaO6	C16H20N3O6	374.1323	M+Na	-0.1	1+	11.6	100.00	8.0	ok	even

Supplementary Fig. 45 HRMS spectrum (positive ionization) of compound Ant-Me-**10**.

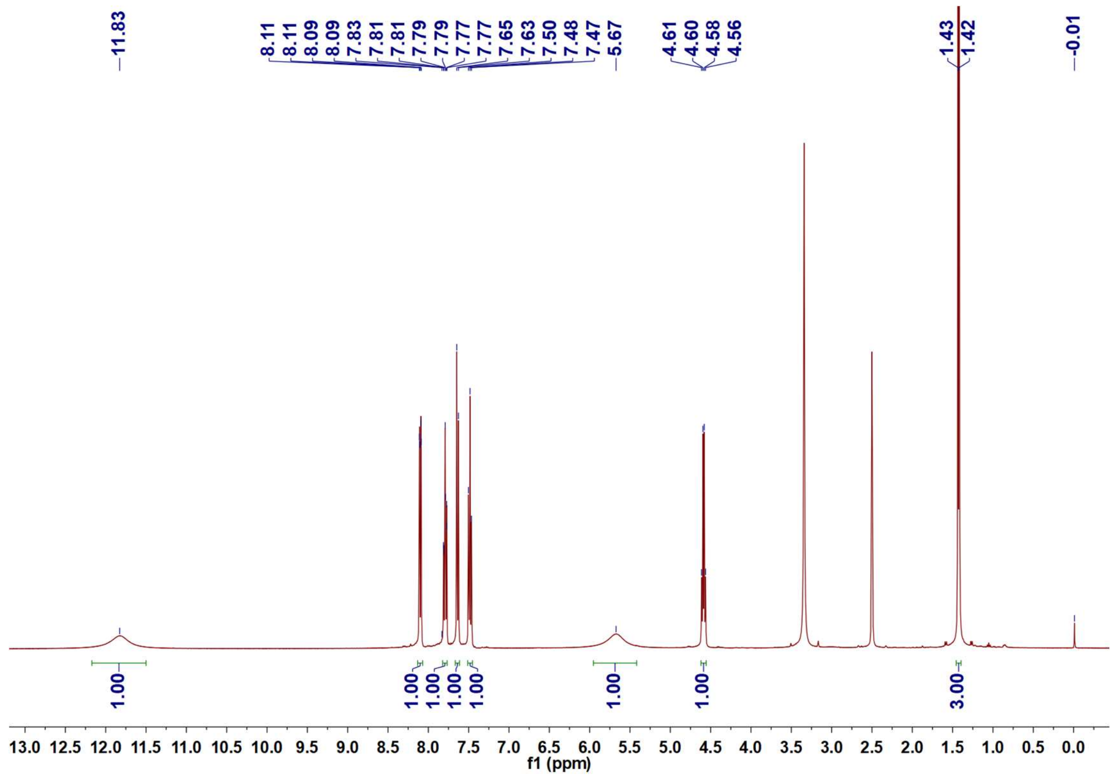

Supplementary Fig. 46 ^{1}H NMR spectrum of compound **1** in DMSO-d_6 (400 MHz).

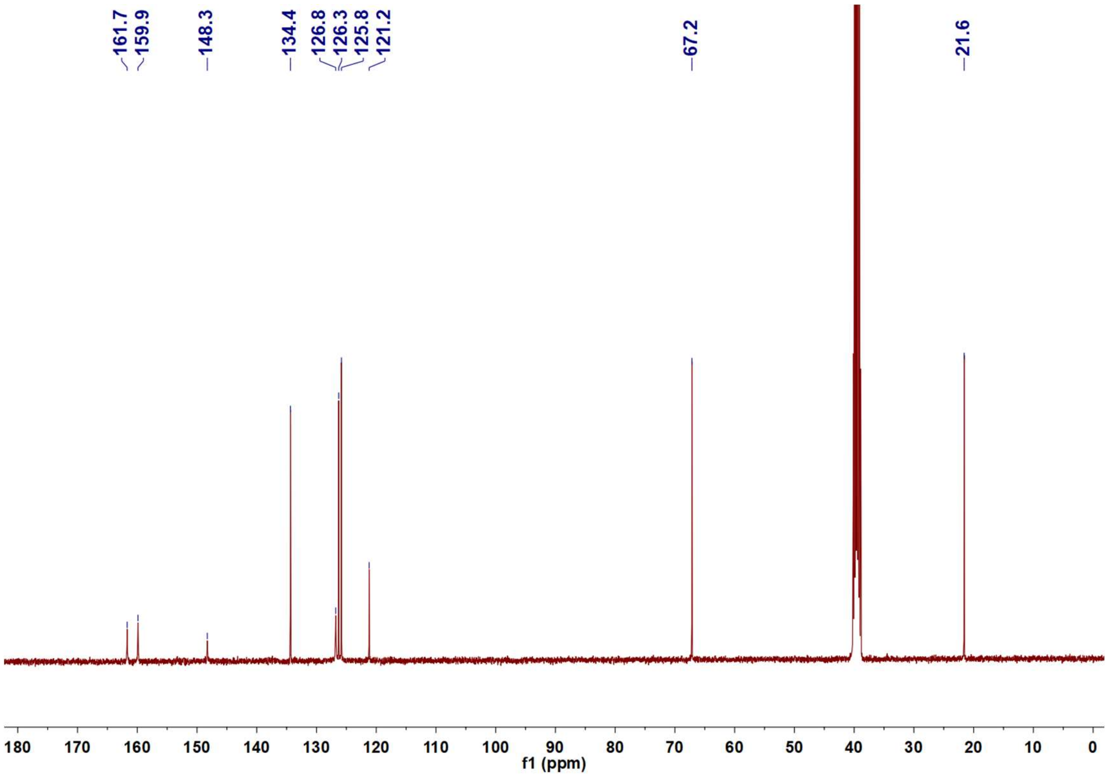

Supplementary Fig. 47 ^{13}C NMR spectrum of compound **1** in DMSO-d_6 (100 MHz).

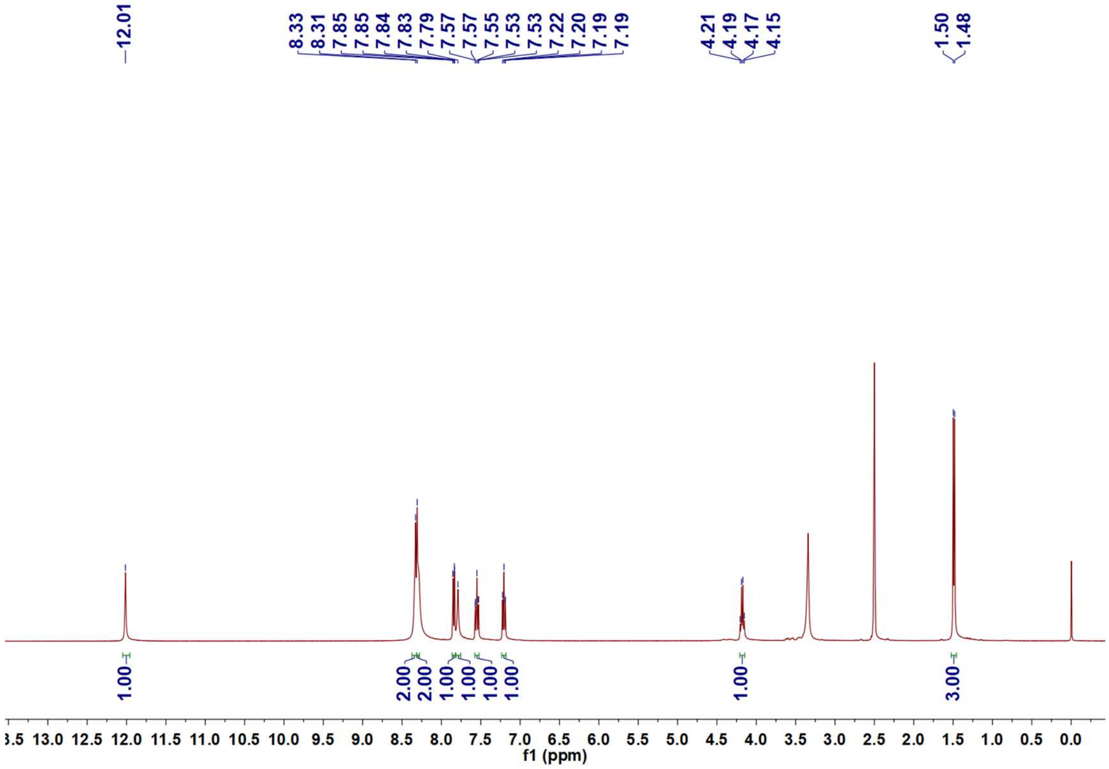

Supplementary Fig. 48 ^{1}H NMR spectrum of compound **2** in DMSO-d_6 (400 MHz).

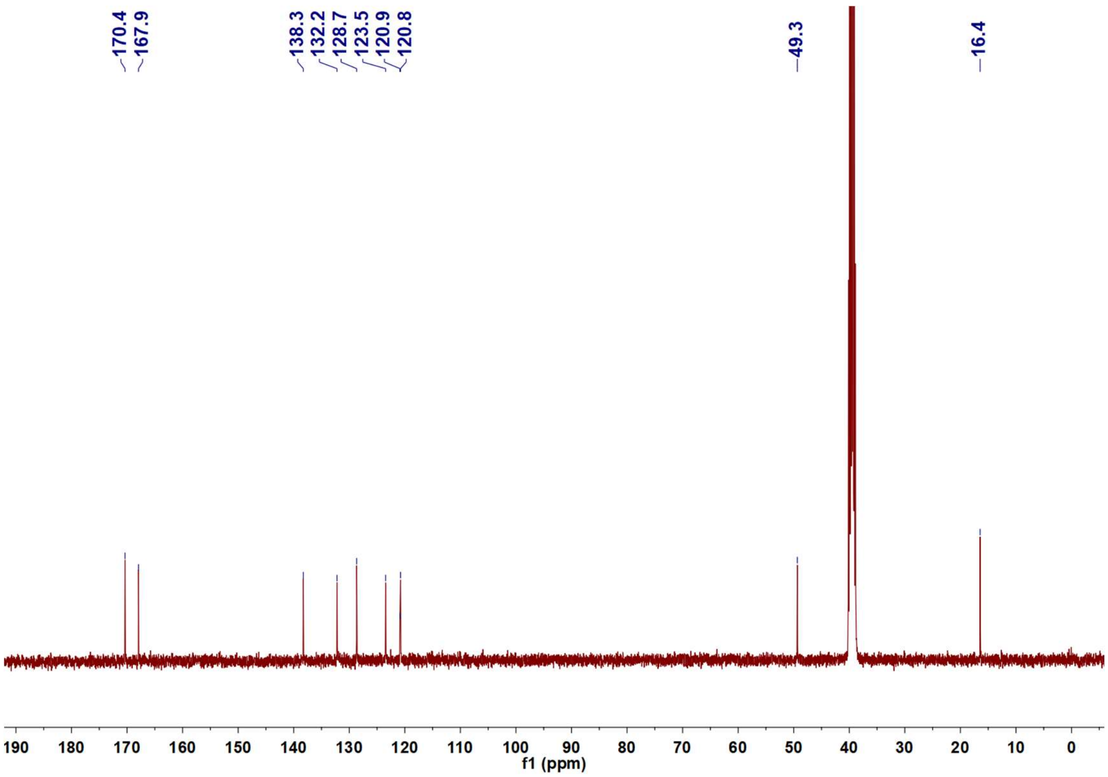

Supplementary Fig. 49 ^{13}C NMR spectrum of compound **2** in DMSO-d_6 (100 MHz).

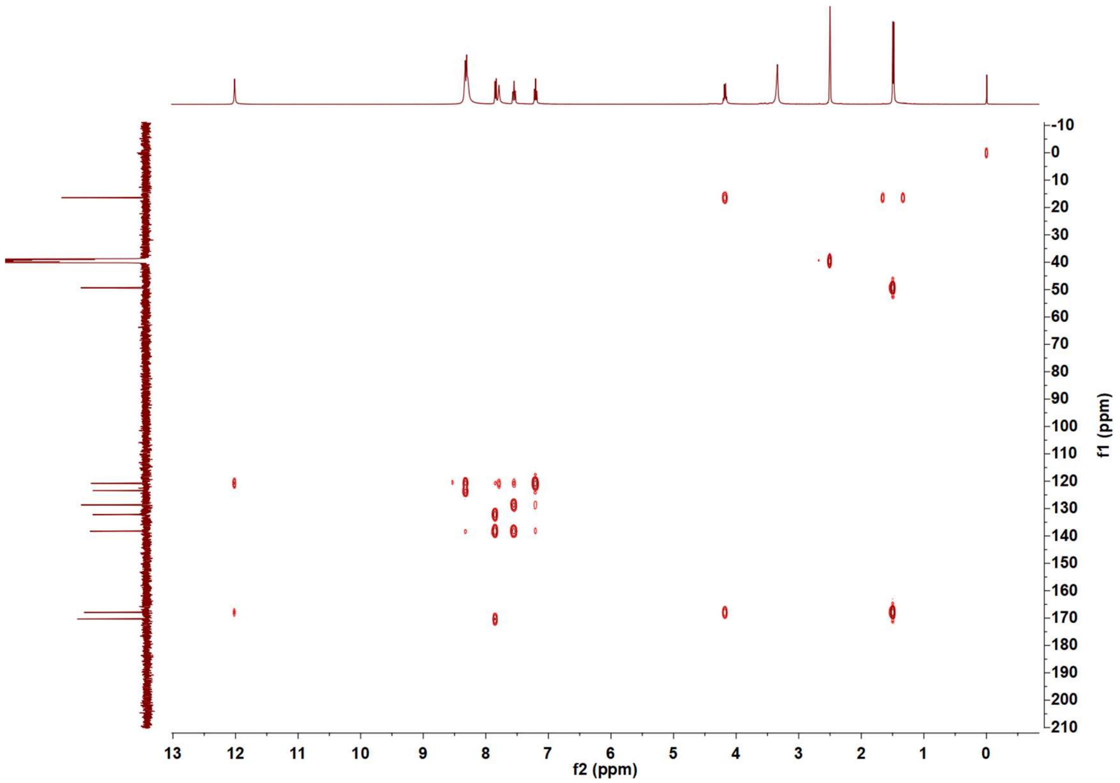

Supplementary Fig. 50 HMBC spectrum of compound **2** in DMSO-d_6.

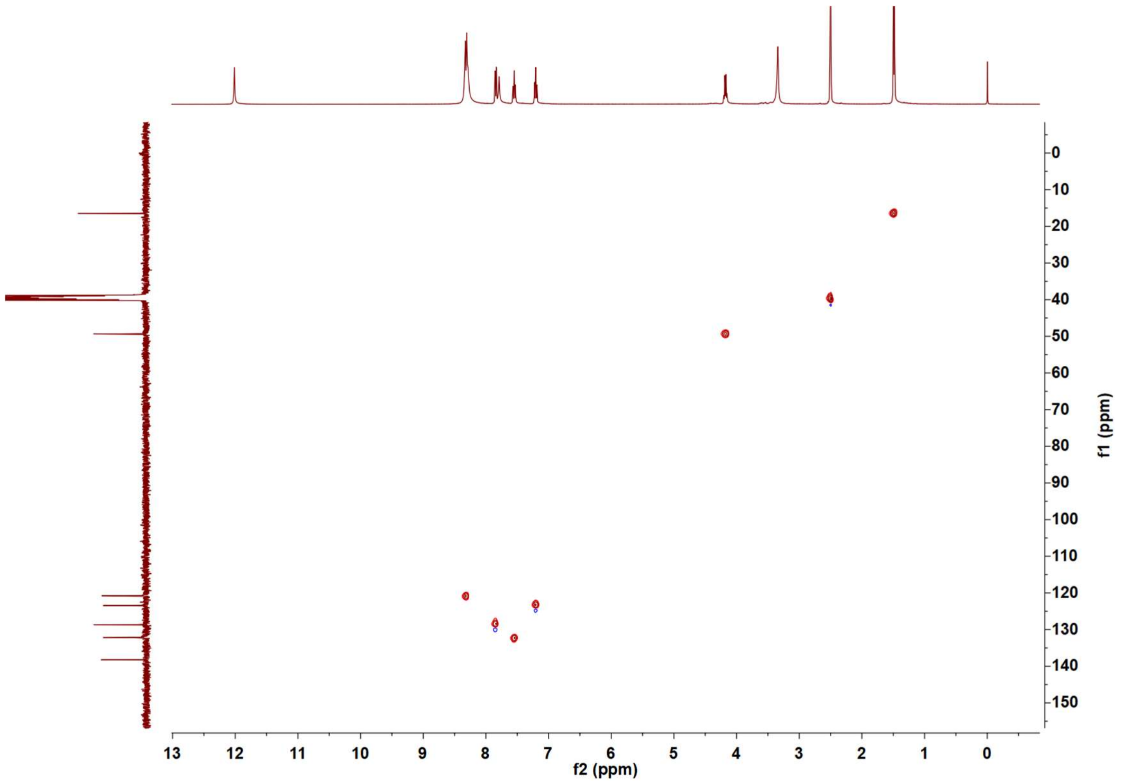

Supplementary Fig. 51 HSQC spectrum of compound **2** in DMSO-d_6.

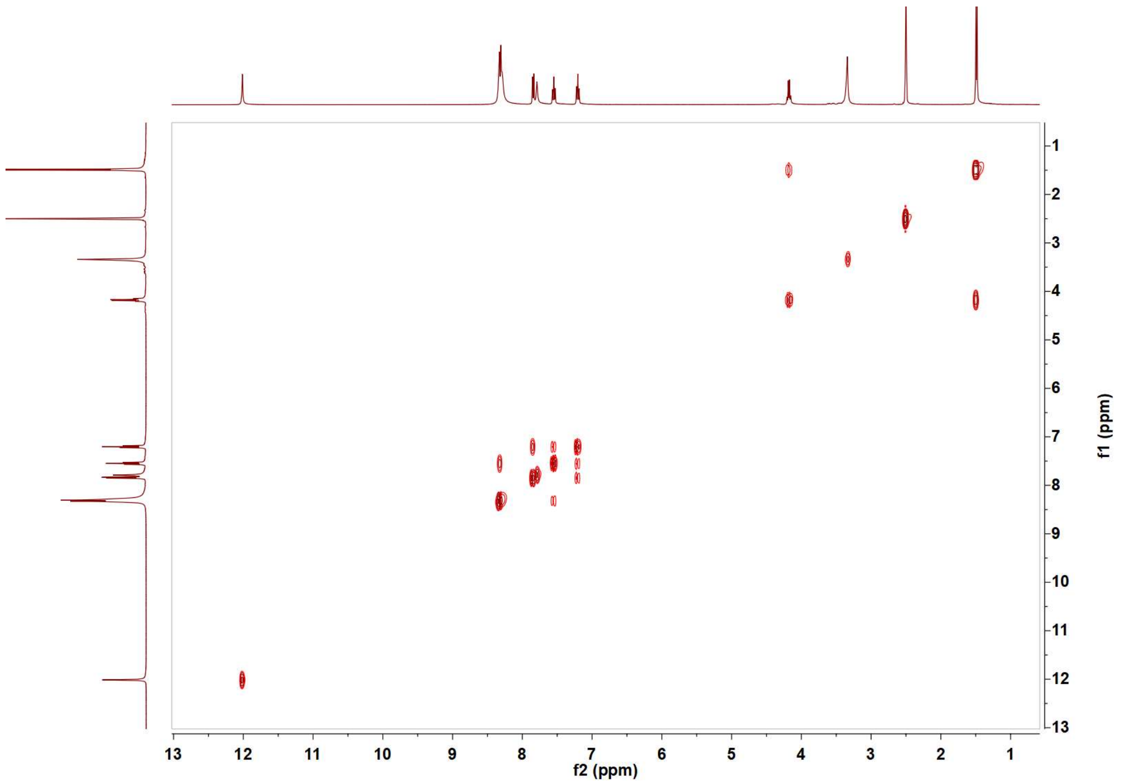

Supplementary Fig. 52 ^{1}H-^{1}H-COSY spectrum of compound **2** in DMSO-d_6.

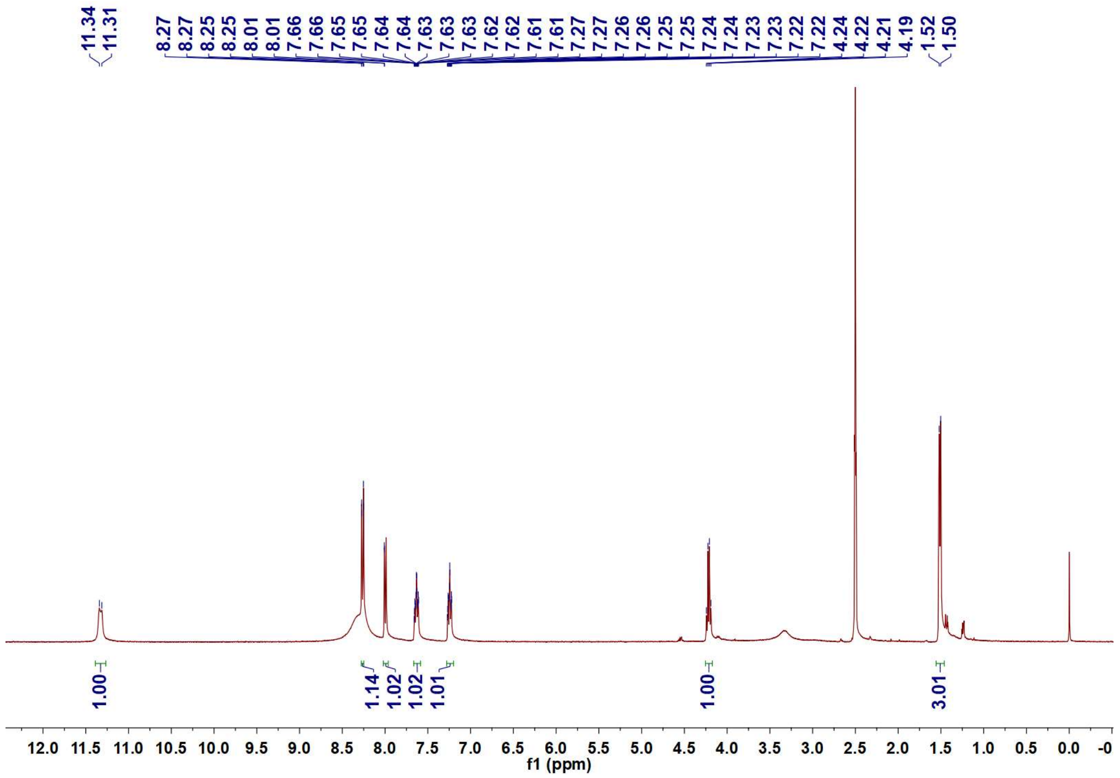

Supplementary Fig. 53 ^{1}H NMR spectrum of compound **3** in DMSO-d_6 (400 MHz).

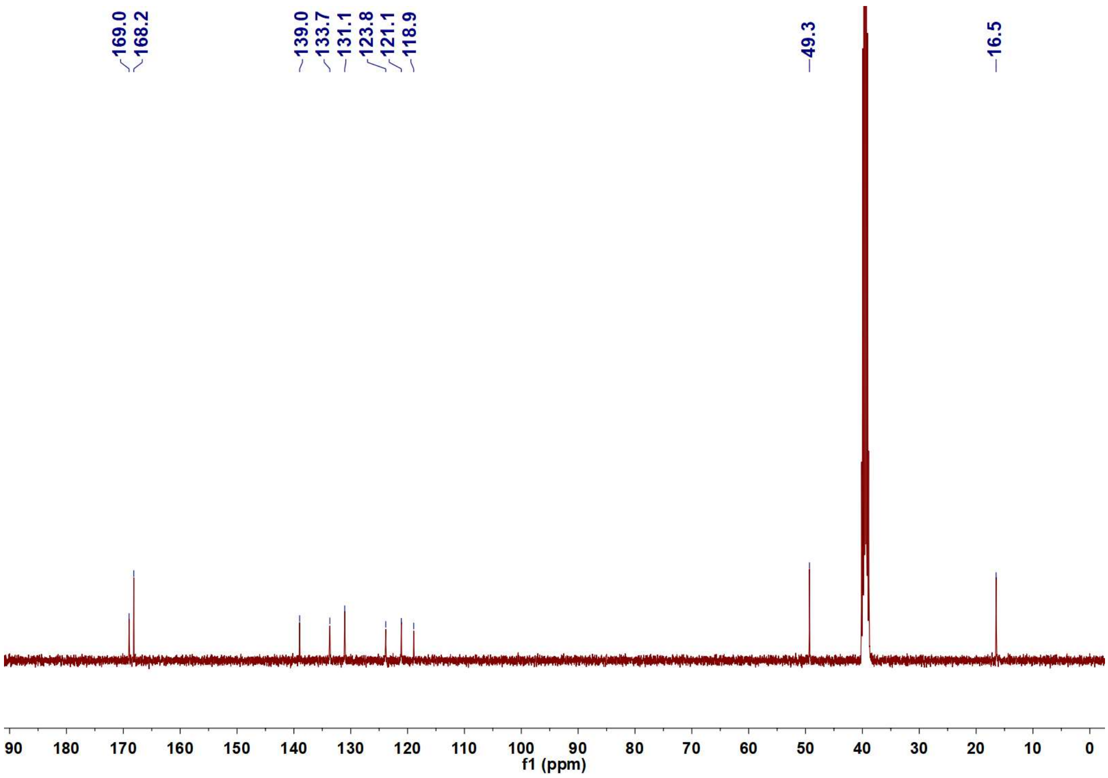

Supplementary Fig. 54 ^{13}C NMR spectrum of compound **3** in DMSO-d_6 (100 MHz).

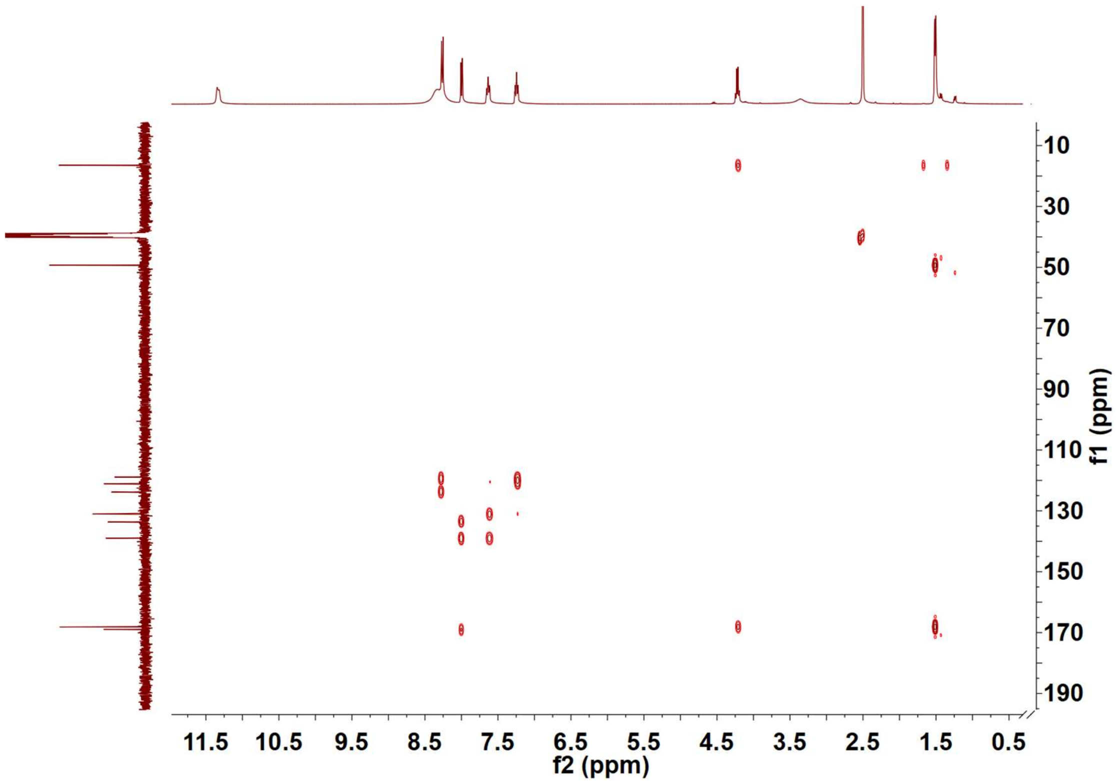

Supplementary Fig. 55 HMBC spectrum of compound **3** in DMSO-d_6.

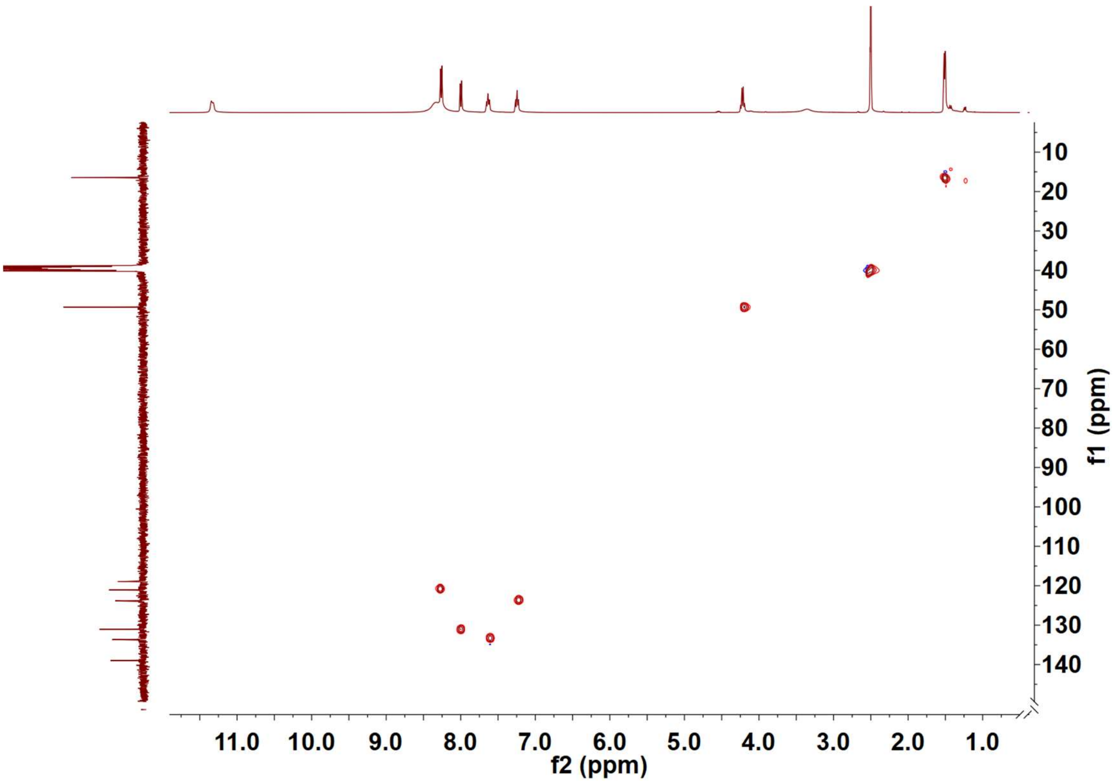

Supplementary Fig. 56 HSQC spectrum of compound **3** in DMSO-d_6.

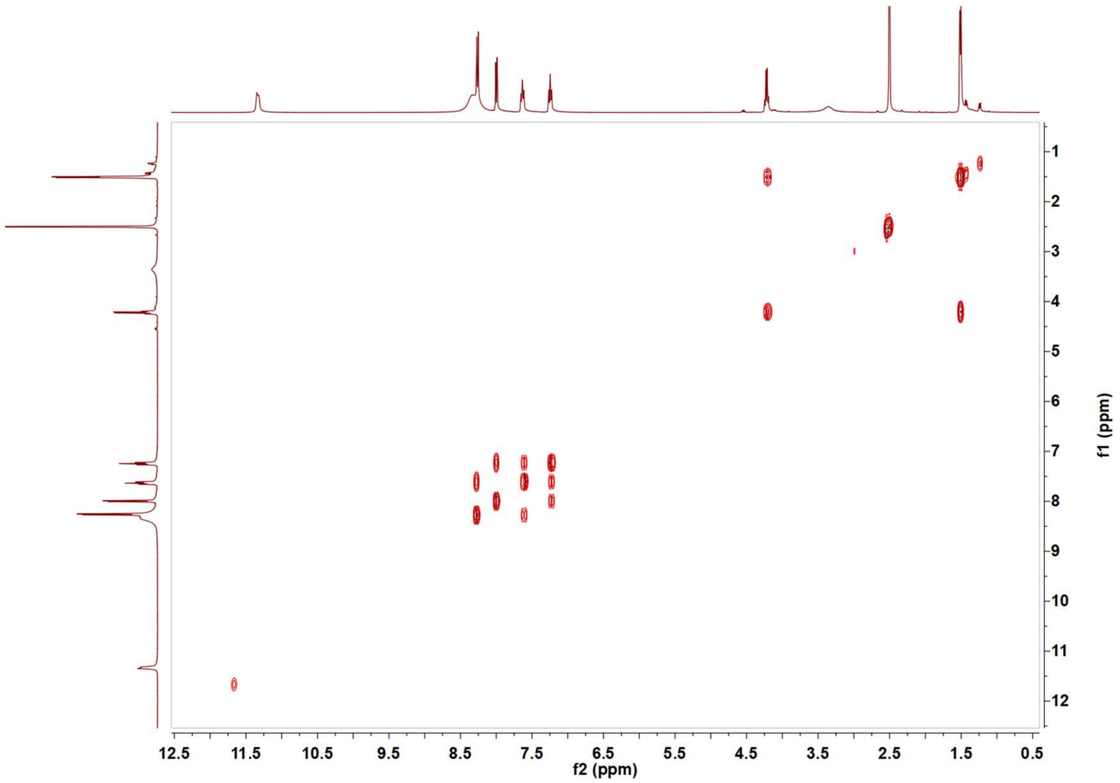

Supplementary Fig. 57 ^{1}H-^{1}H-COSY spectrum of compound **3** in DMSO-d_6.

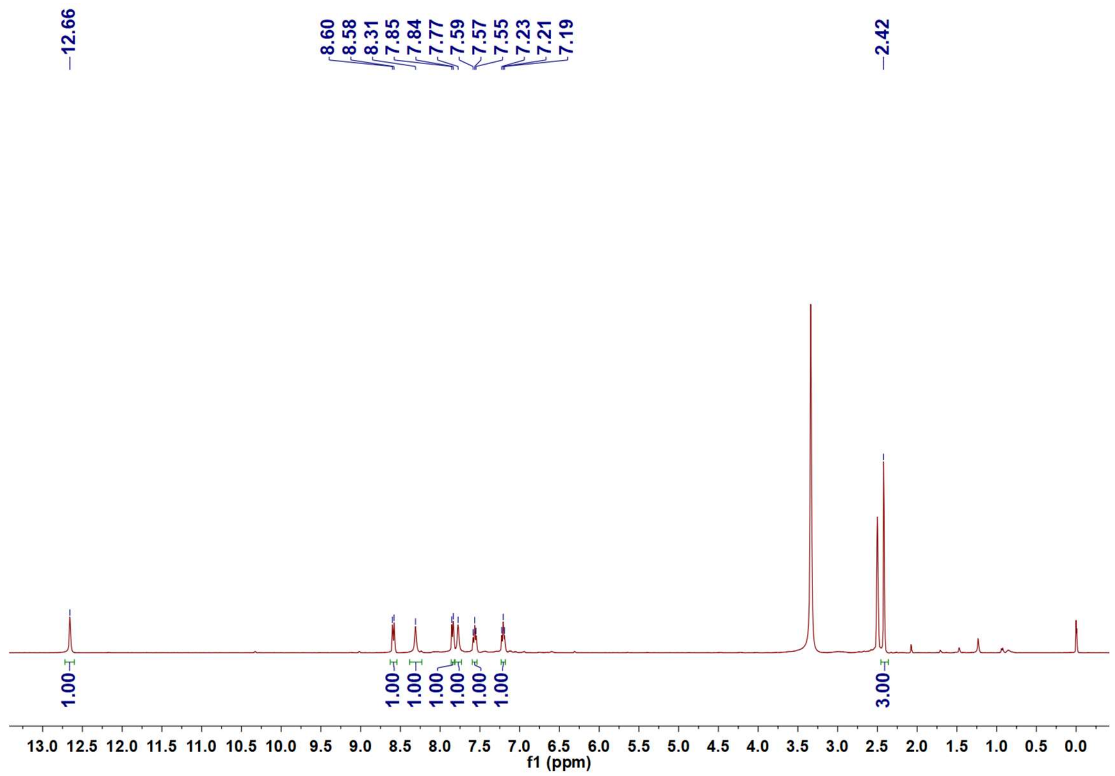

Supplementary Fig. 58 ^{1}H NMR spectrum of compound **4** in DMSO-d_6 (400 MHz).

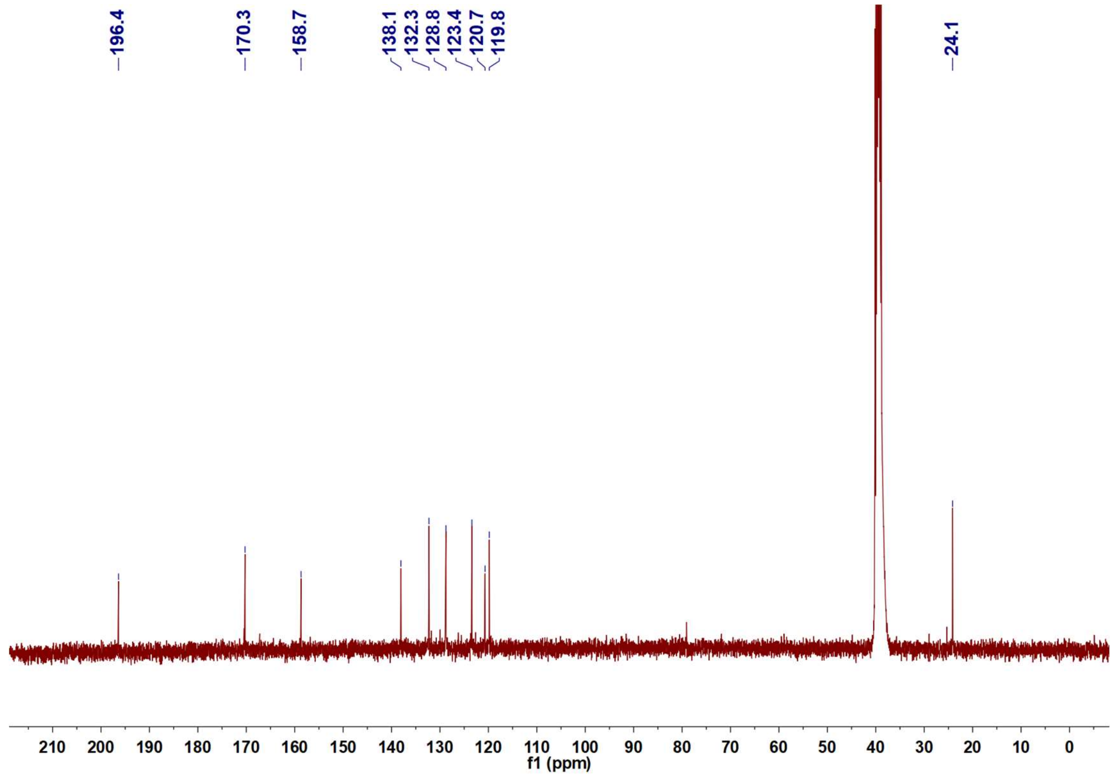

Supplementary Fig. 59 ^{13}C NMR spectrum of compound **4** in DMSO-d_6 (100 MHz).

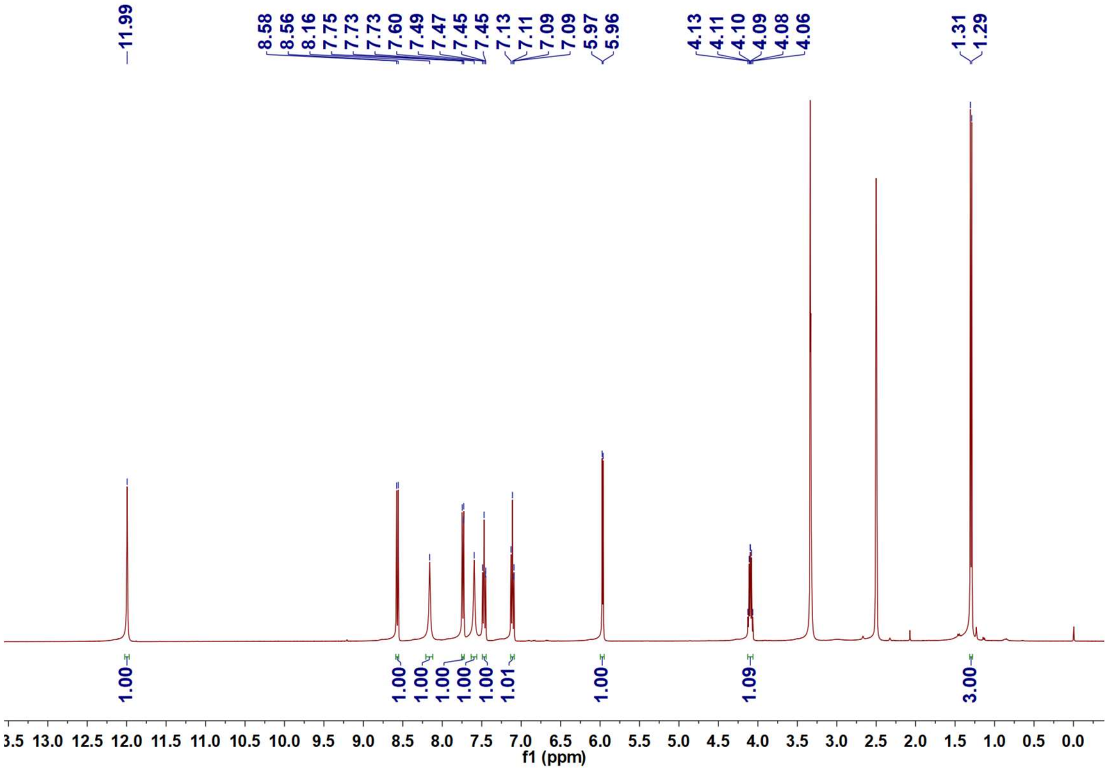

Supplementary Fig. 60 ^{1}H NMR spectrum of compound **5** in DMSO-d_6 (400 MHz).

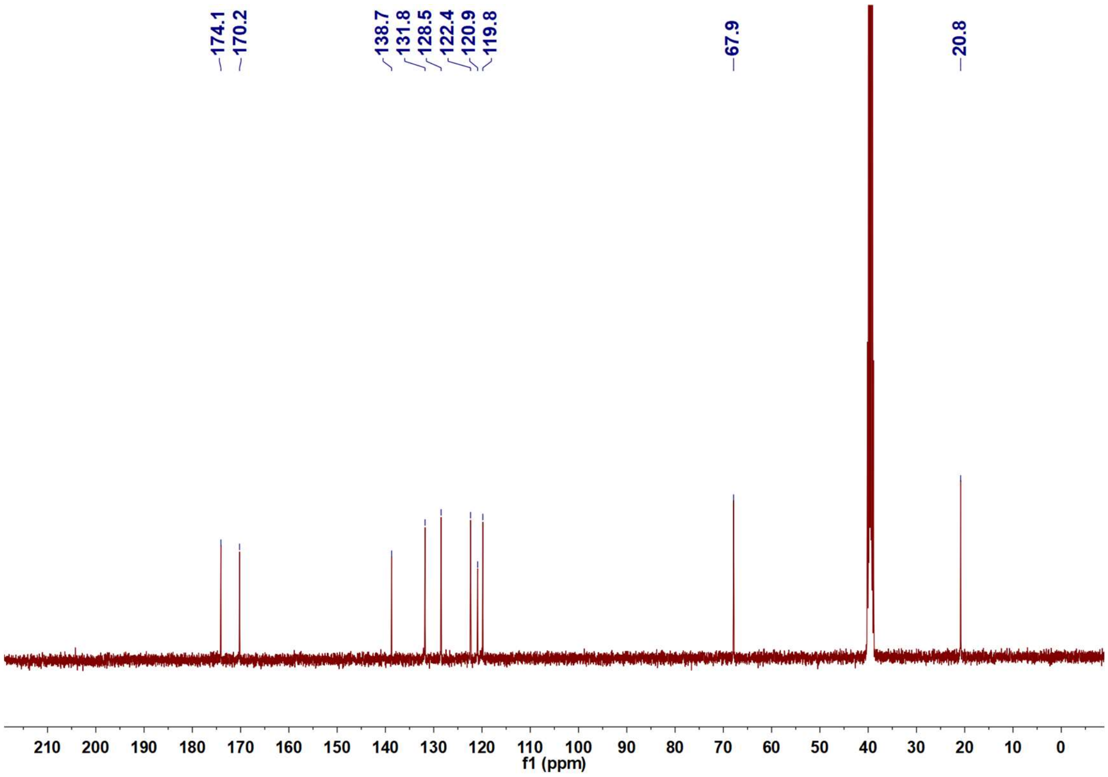

Supplementary Fig. 61 ^{13}C NMR spectrum of compound **5** in DMSO-d_6 (100 MHz).

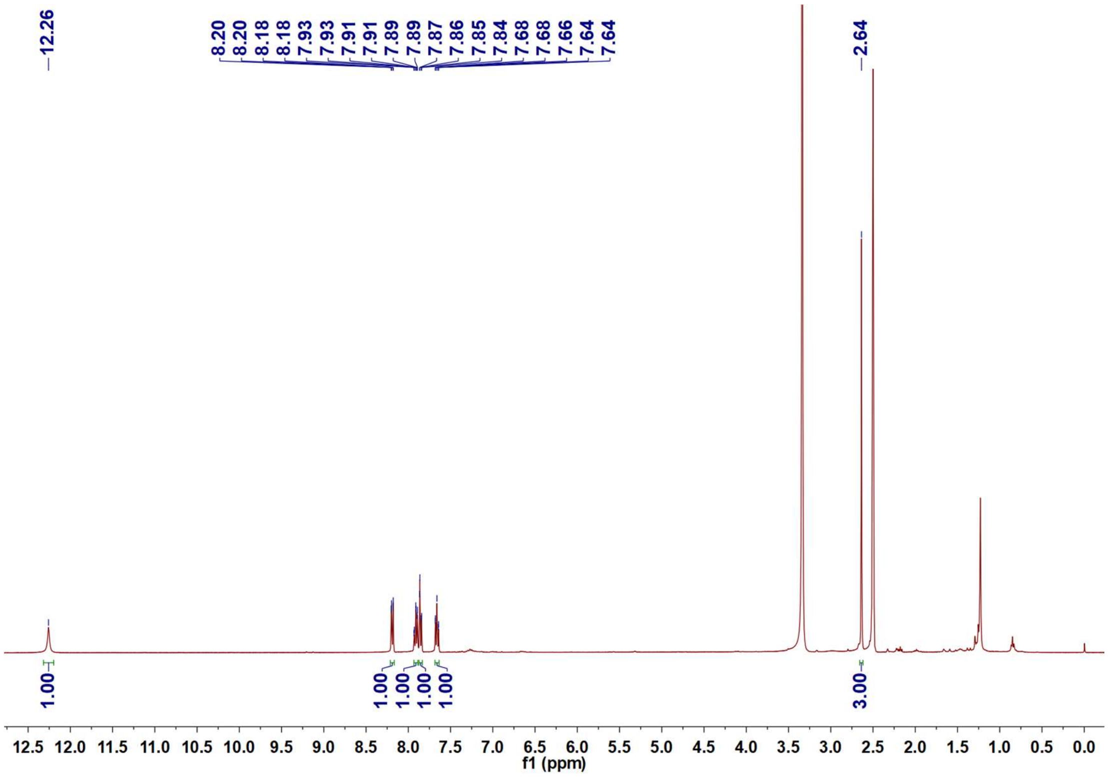

Supplementary Fig. 62 ^{1}H NMR spectrum of compound **6** in DMSO-d_6 (400 MHz).

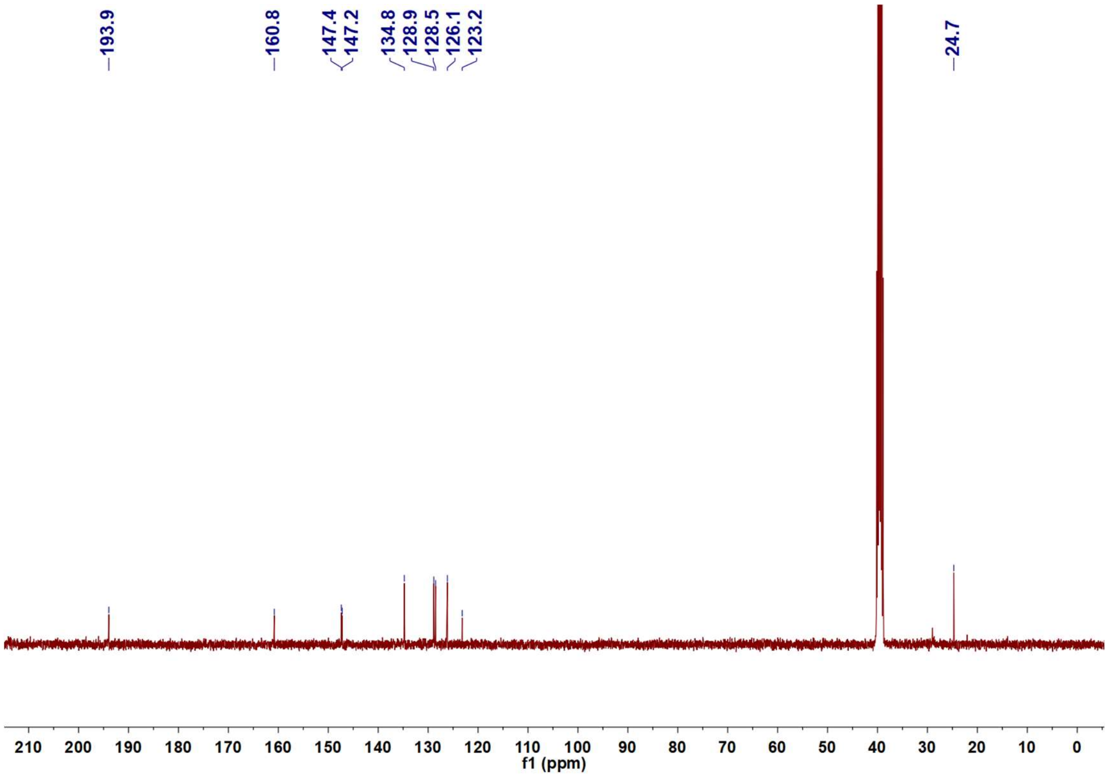

Supplementary Fig. 63 ^{13}C NMR spectrum of compound **6** in DMSO-d_6 (100 MHz).

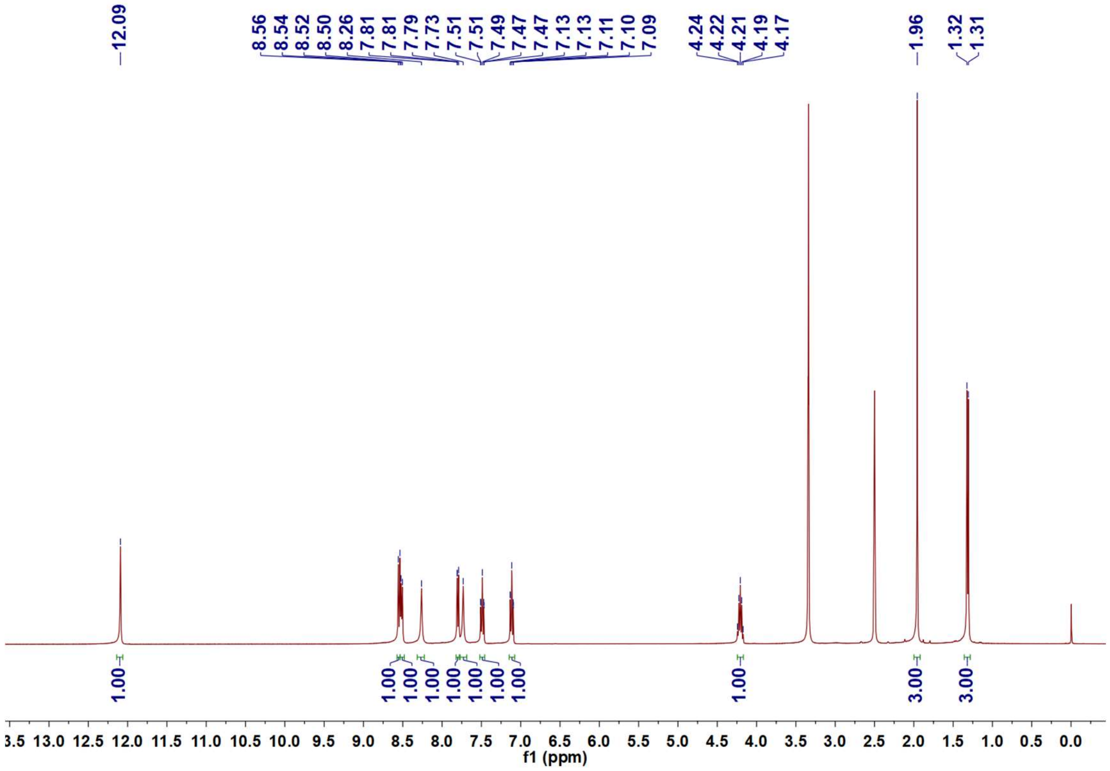

Supplementary Fig. 64 ^{1}H NMR spectrum of compound **7** in DMSO-d_6 (400 MHz).

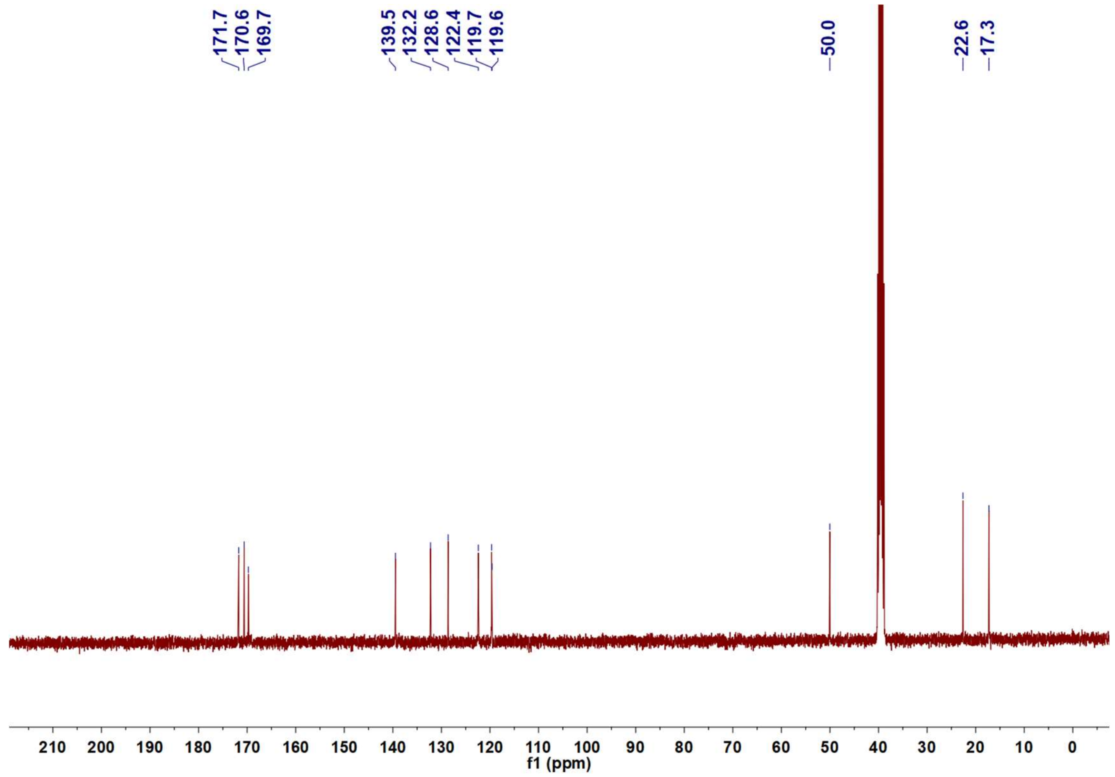

Supplementary Fig. 65 ^{13}C NMR spectrum of compound **7** in DMSO-d_6 (100 MHz).

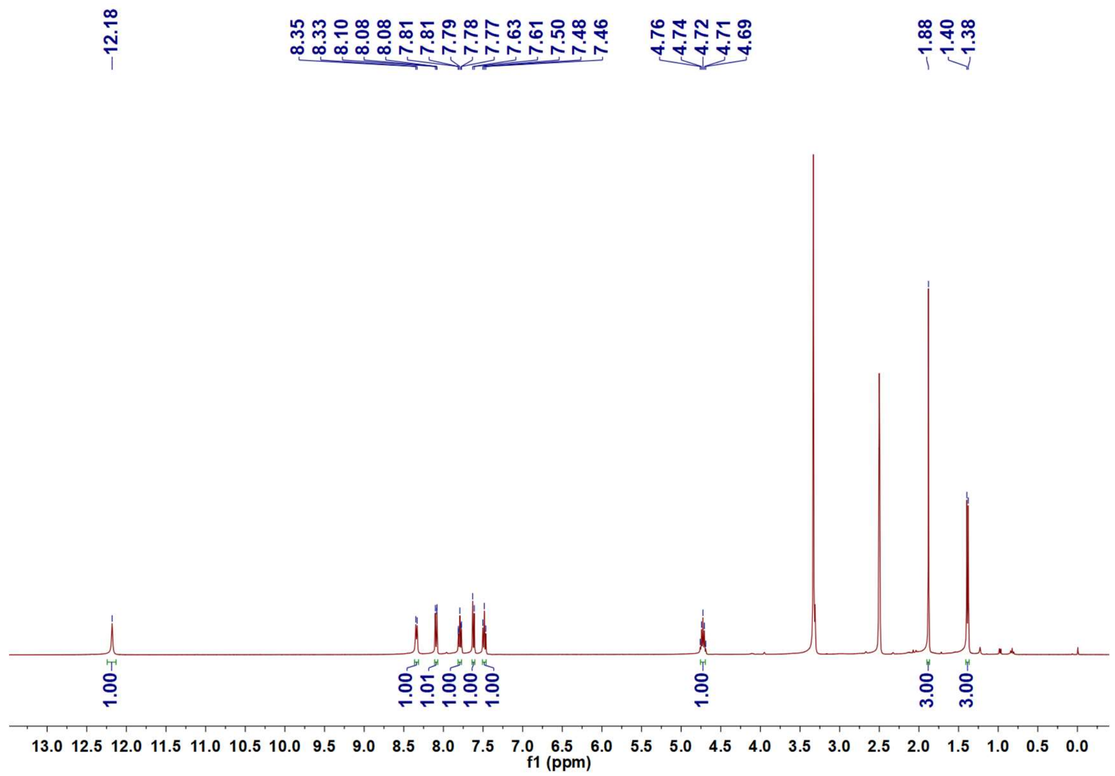

Supplementary Fig. 66 ^{1}H NMR spectrum of compound **8** in DMSO-d_6 (400 MHz).

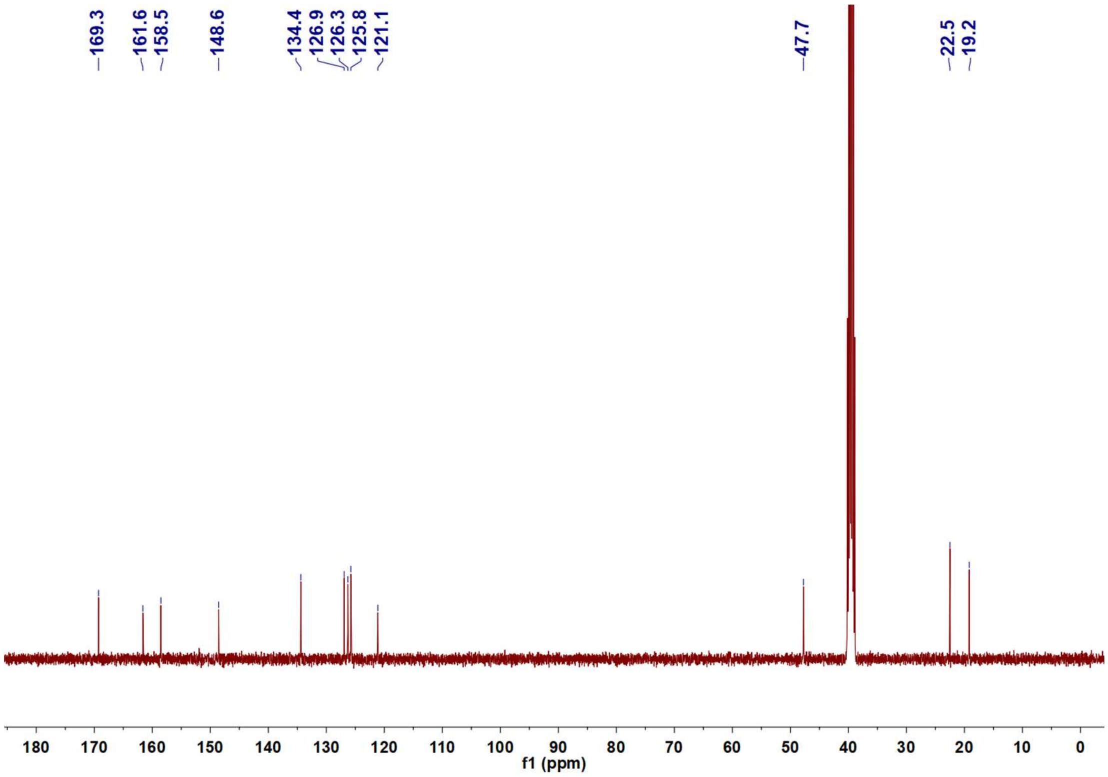

Supplementary Fig. 67 ^{13}C NMR spectrum of compound **8** in DMSO-d_6 (100 MHz).

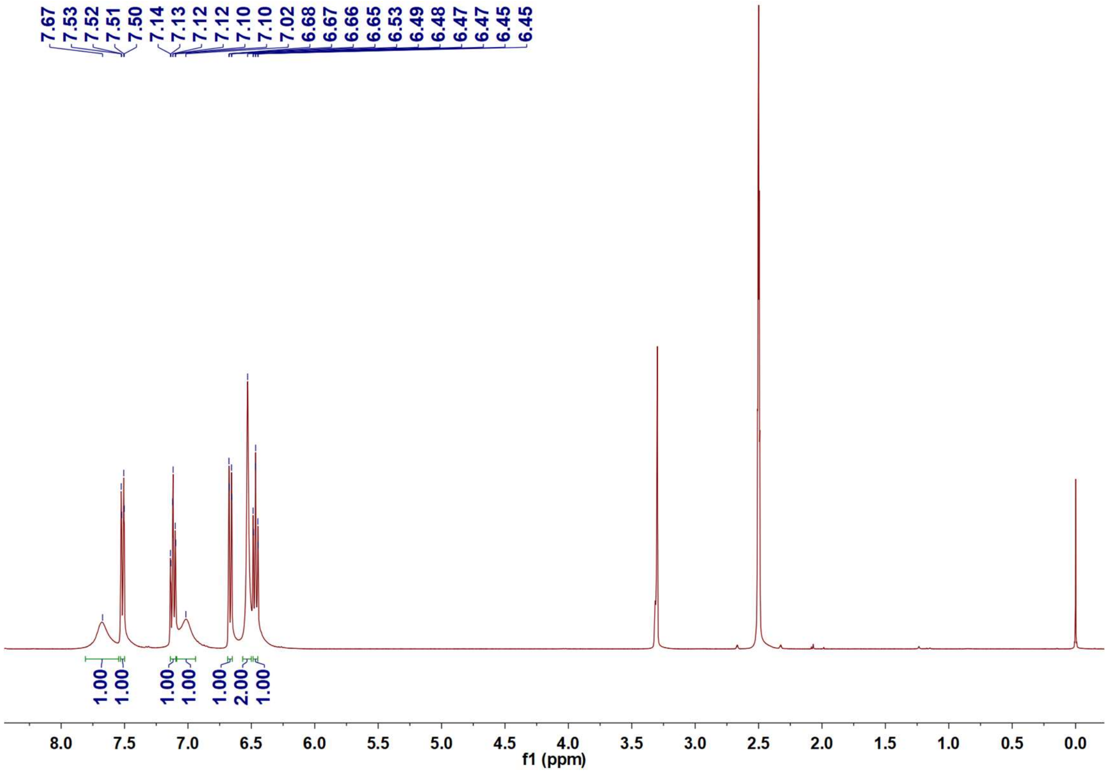

Supplementary Fig. 68 ^{1}H NMR spectrum of compound **9** in DMSO-d_6 (400 MHz).

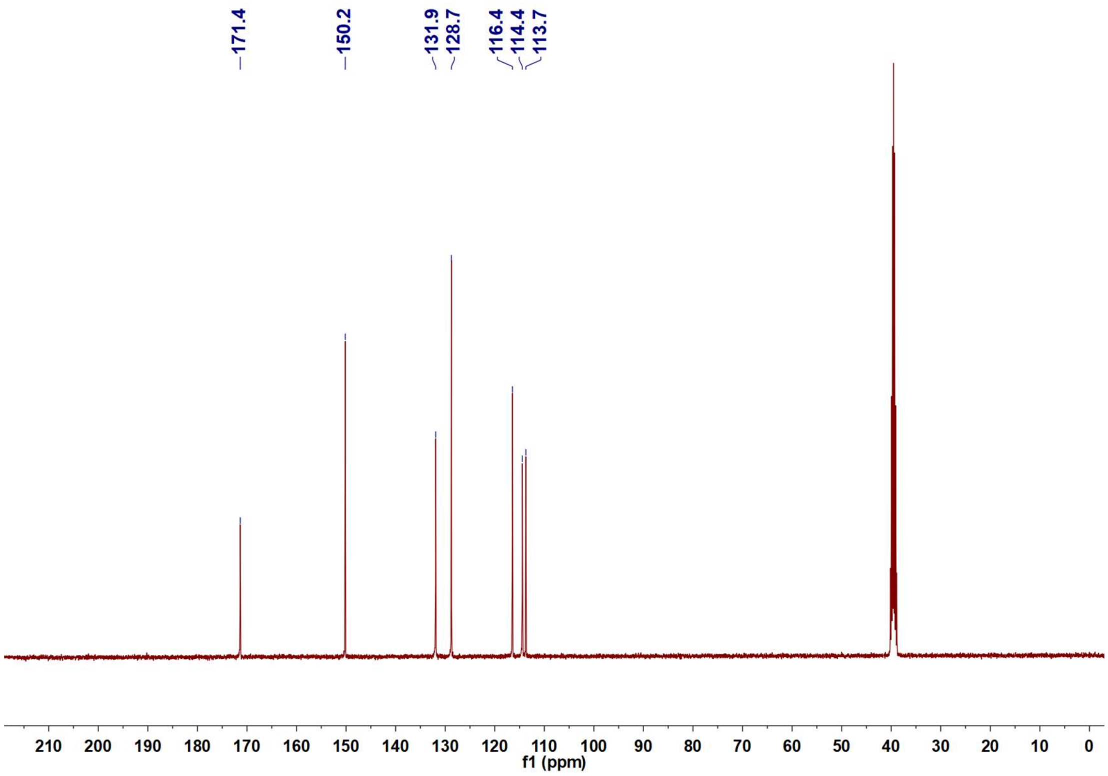

Supplementary Fig. 69 ^{13}C NMR spectrum of compound **9** in DMSO-d_6 (100 MHz).

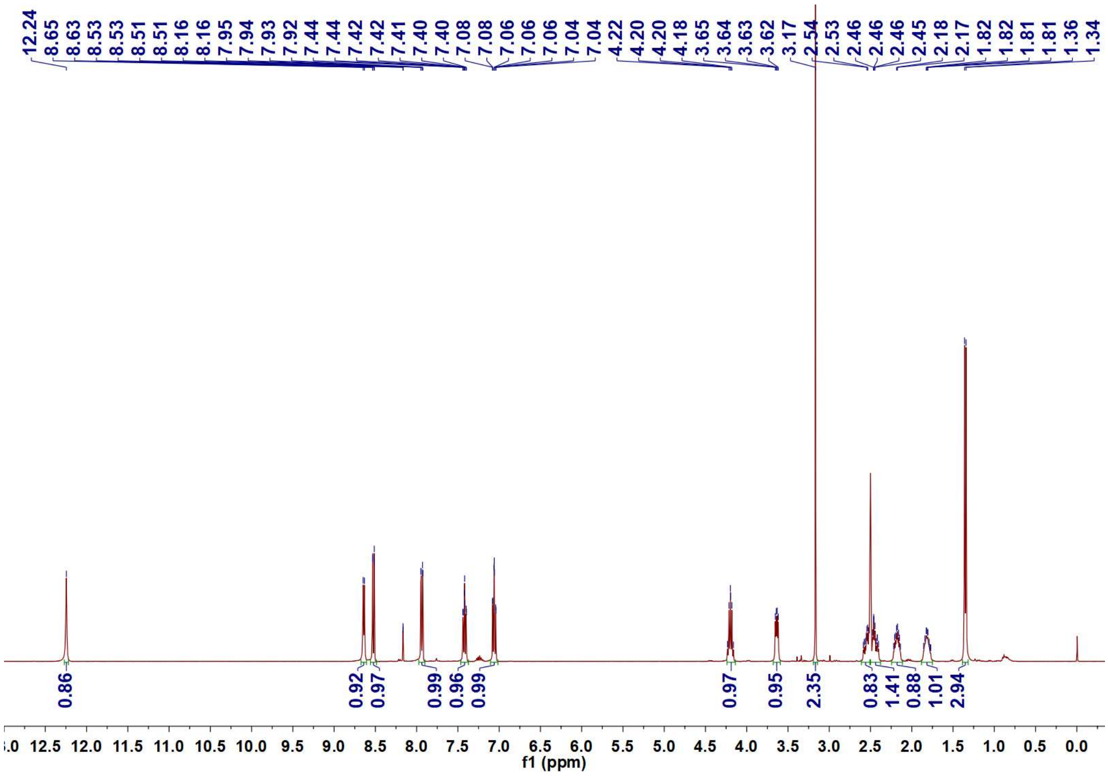

Supplementary Fig. 70 ^{1}H NMR spectrum of compound **10** in DMSO-d_6 (400 MHz).

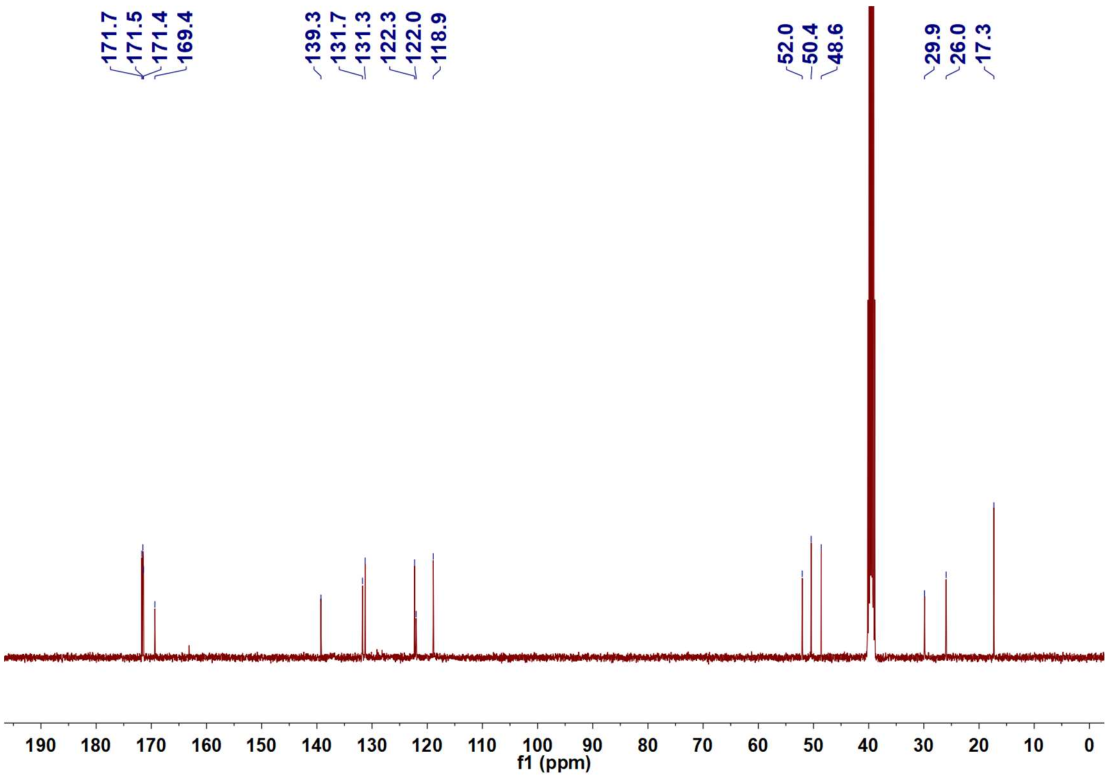

Supplementary Fig. 71 ^{13}C NMR spectrum of compound **10** in DMSO-d_6 (100 MHz).

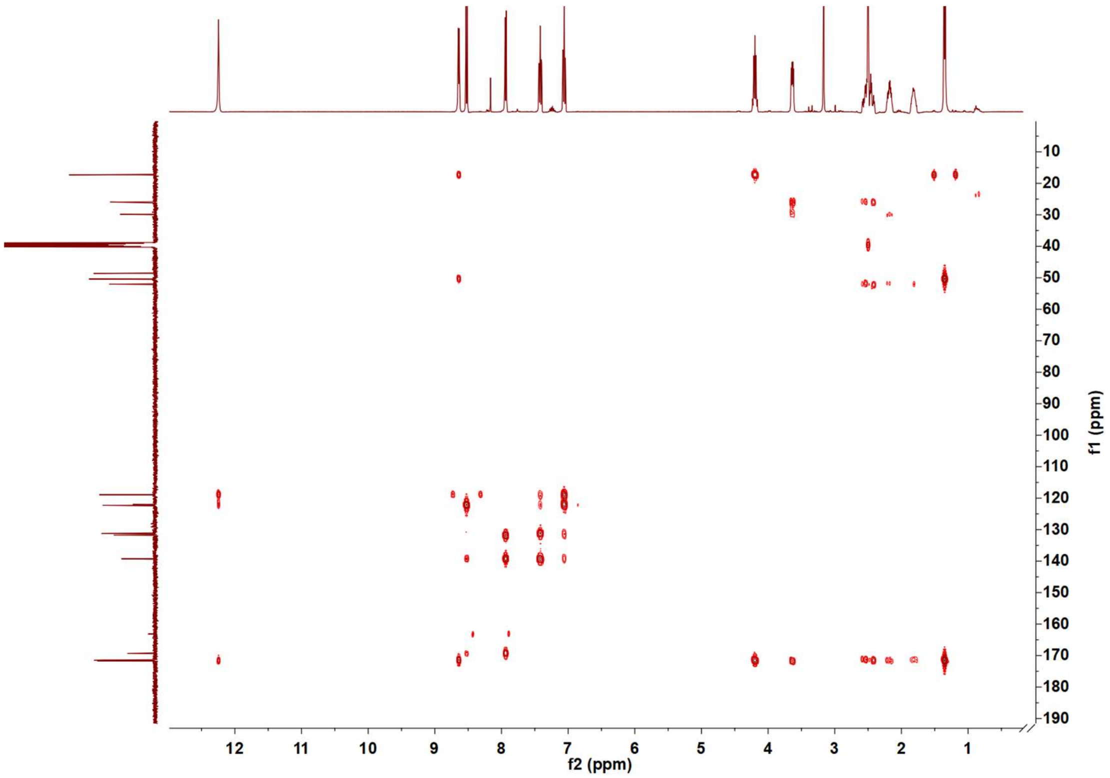

Supplementary Fig. 72 HMBC spectrum of compound **10** in DMSO-d_6.

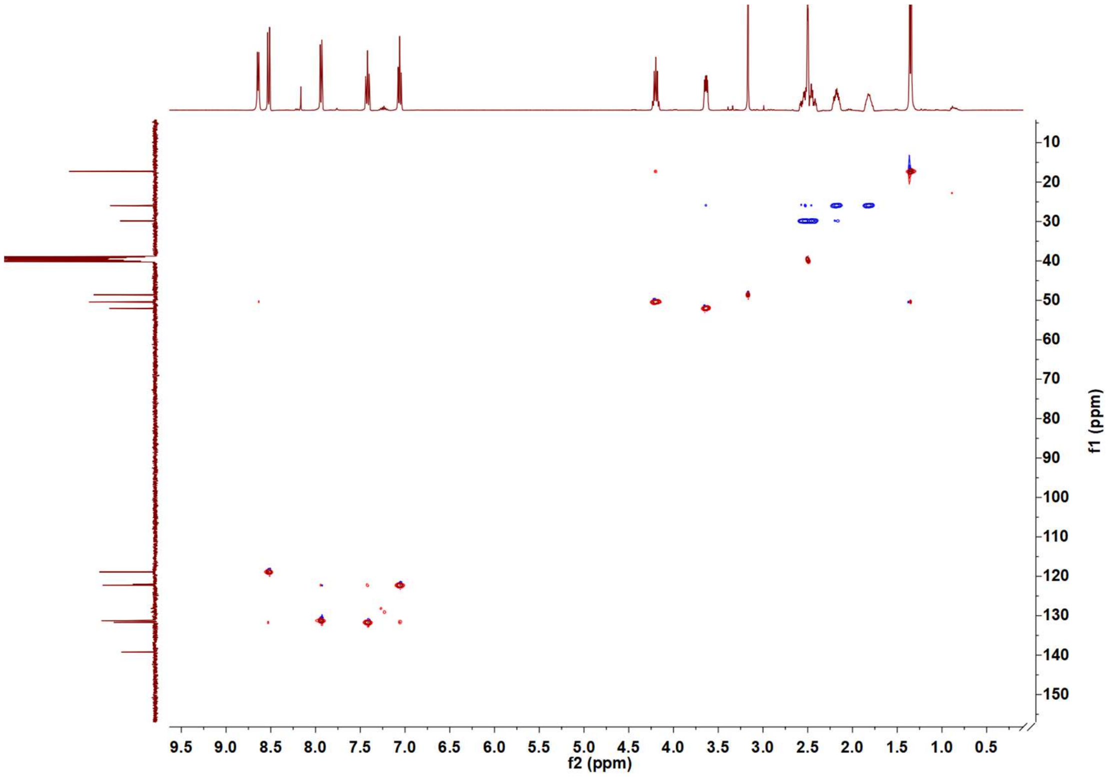

Supplementary Fig. 73 HSQC spectrum of compound **10** in DMSO-d_6.

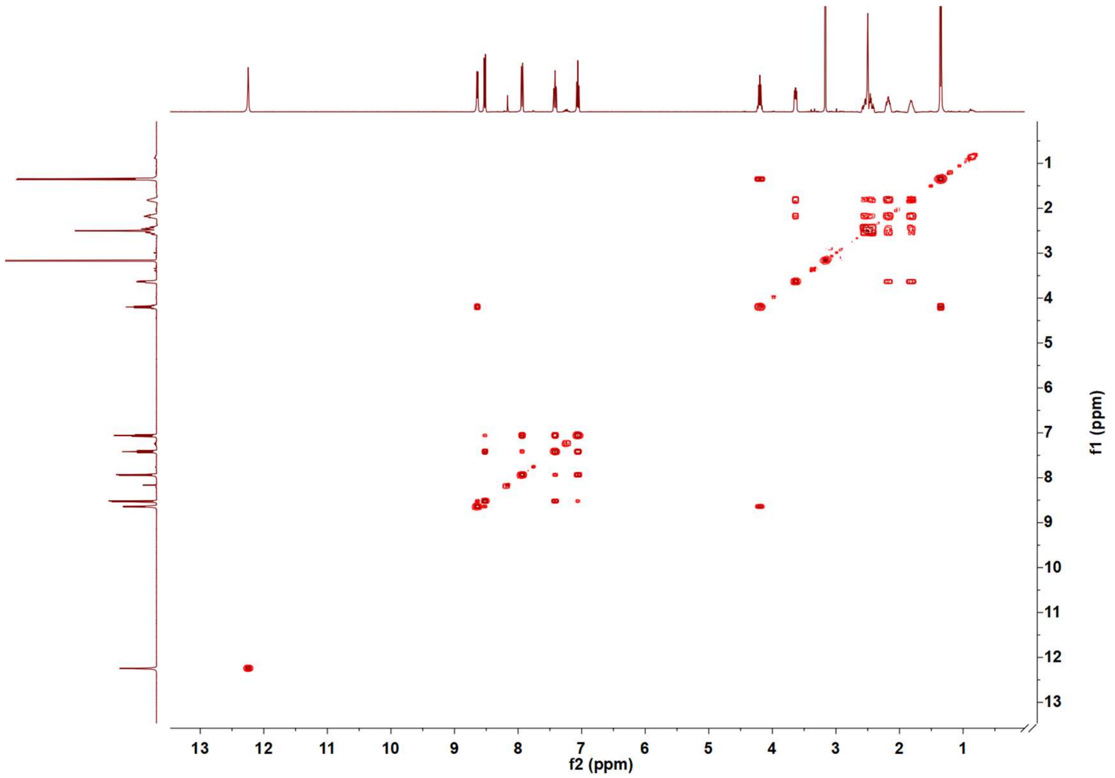

Supplementary Fig. 74 ^{1}H-^{1}H COSY spectrum of compound **10** in DMSO-d_6.

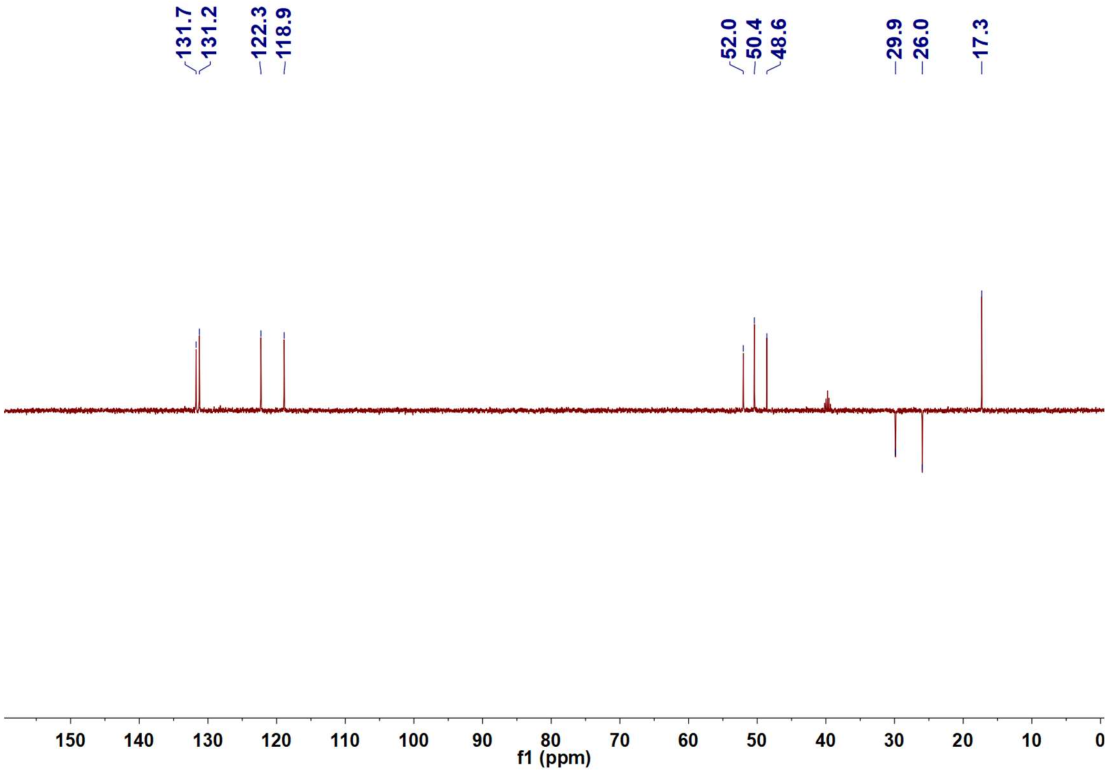

Supplementary Fig. 75 DEPT-135° spectrum of compound **10** in DMSO-d_6.

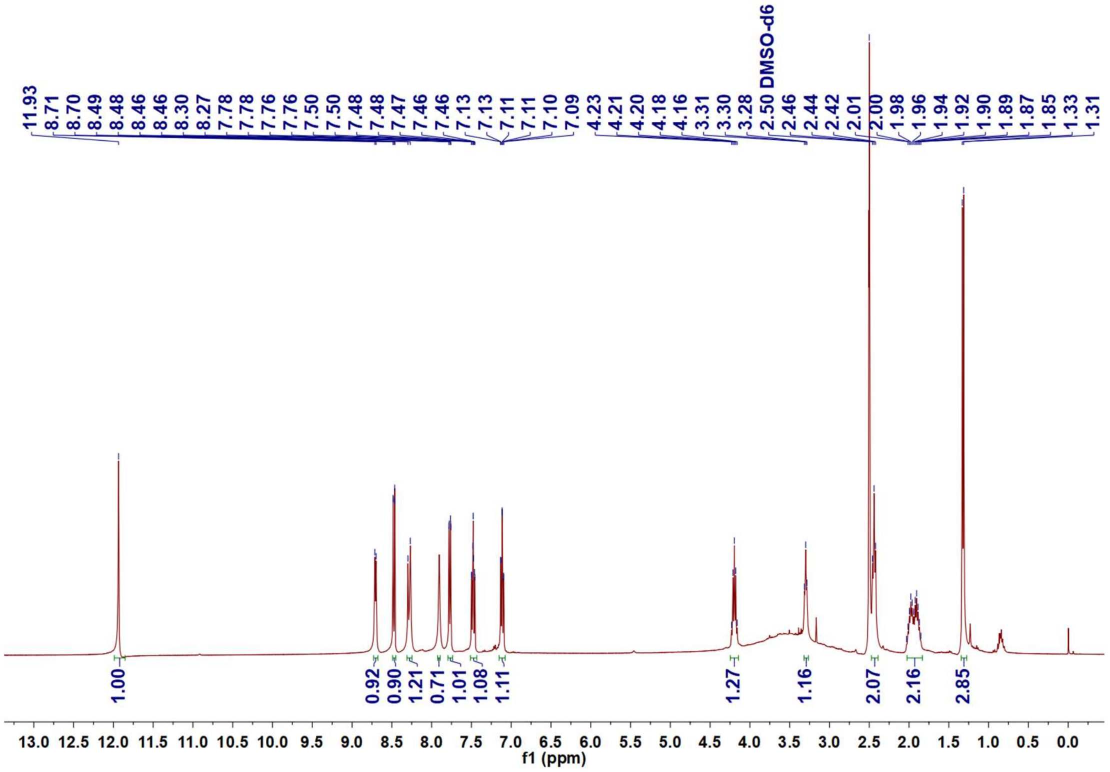

Supplementary Fig. 76 ^{1}H NMR spectrum of compound **11** in DMSO-d_6 (400 MHz).

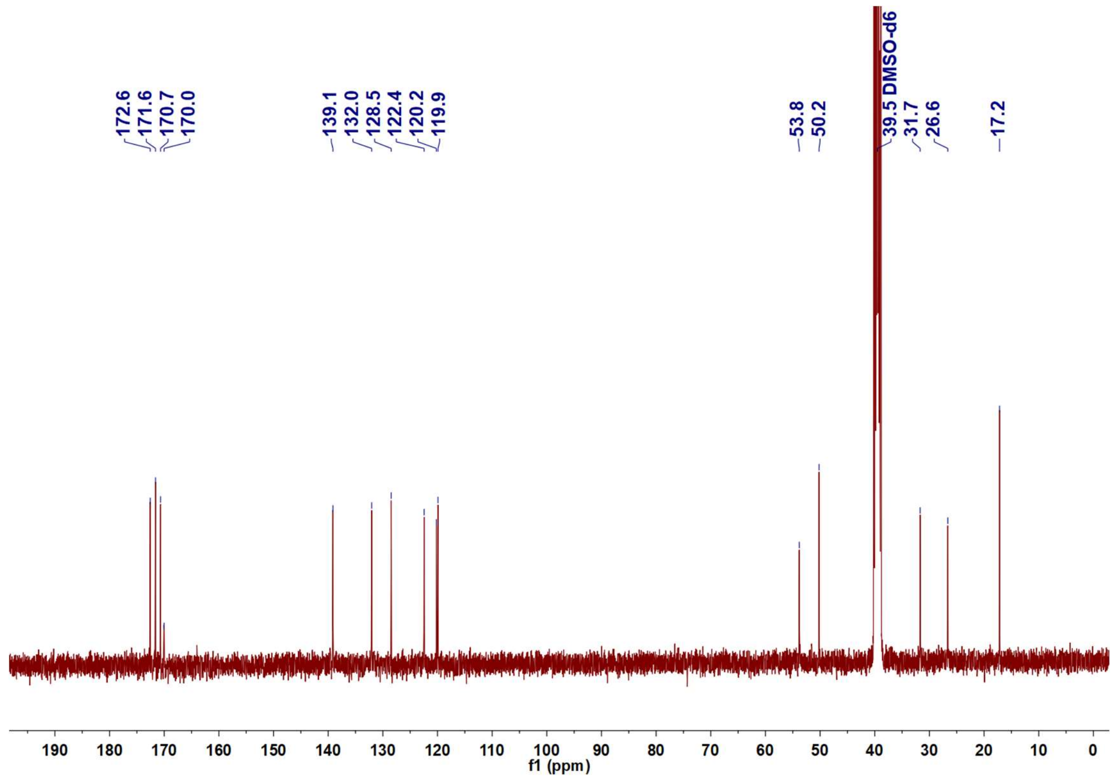

Supplementary Fig. 77 ^{13}C NMR spectrum of compound **11** in DMSO-d_6 (100 MHz).

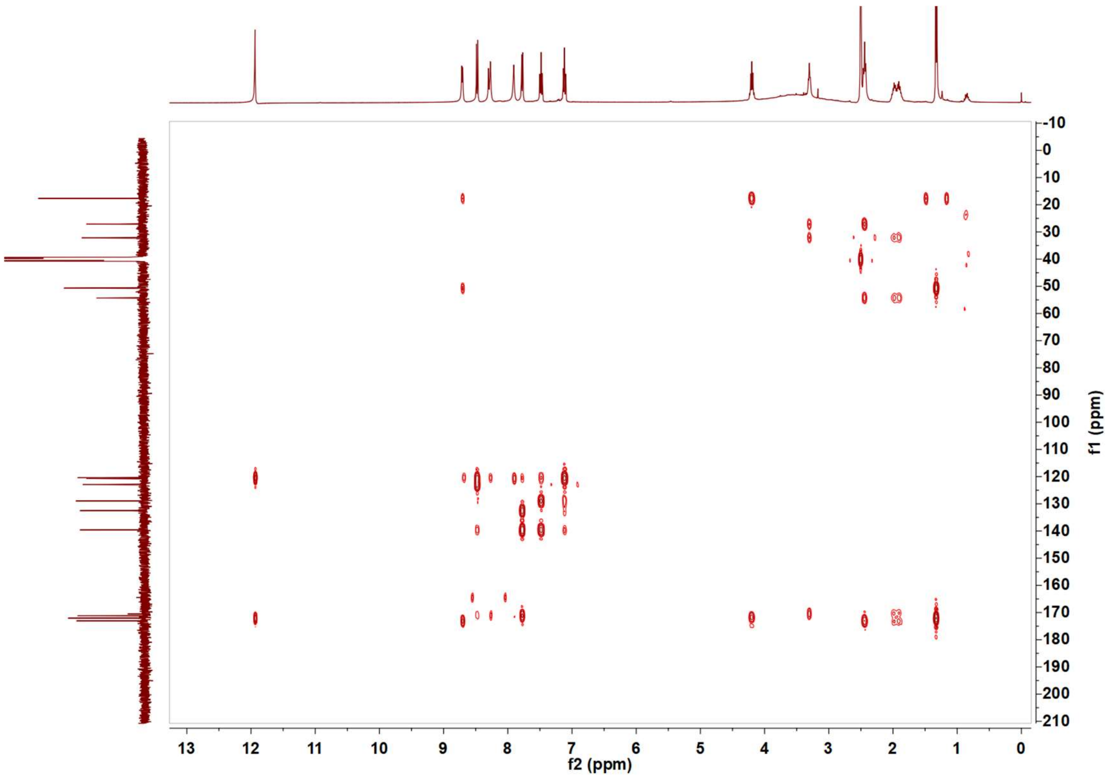

Supplementary Fig. 78 HMBC spectrum of compound **11** in DMSO-d_6.

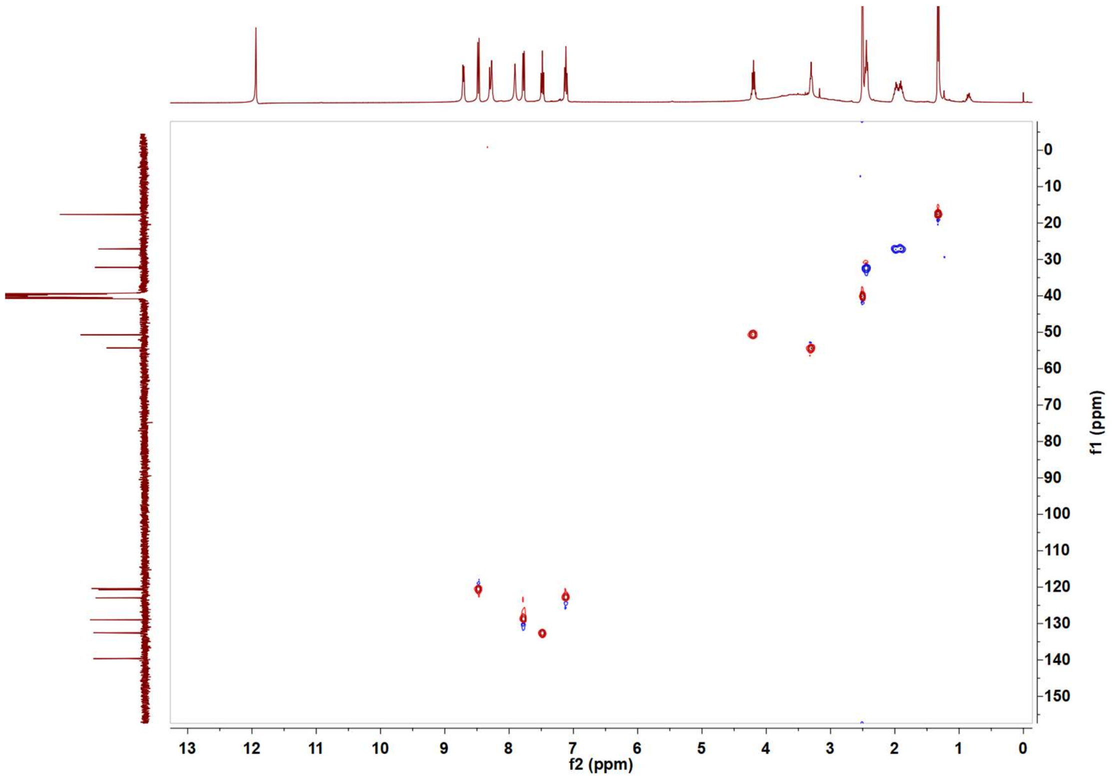

Supplementary Fig. 79 HSQC spectrum of compound **11** in DMSO-d_6.

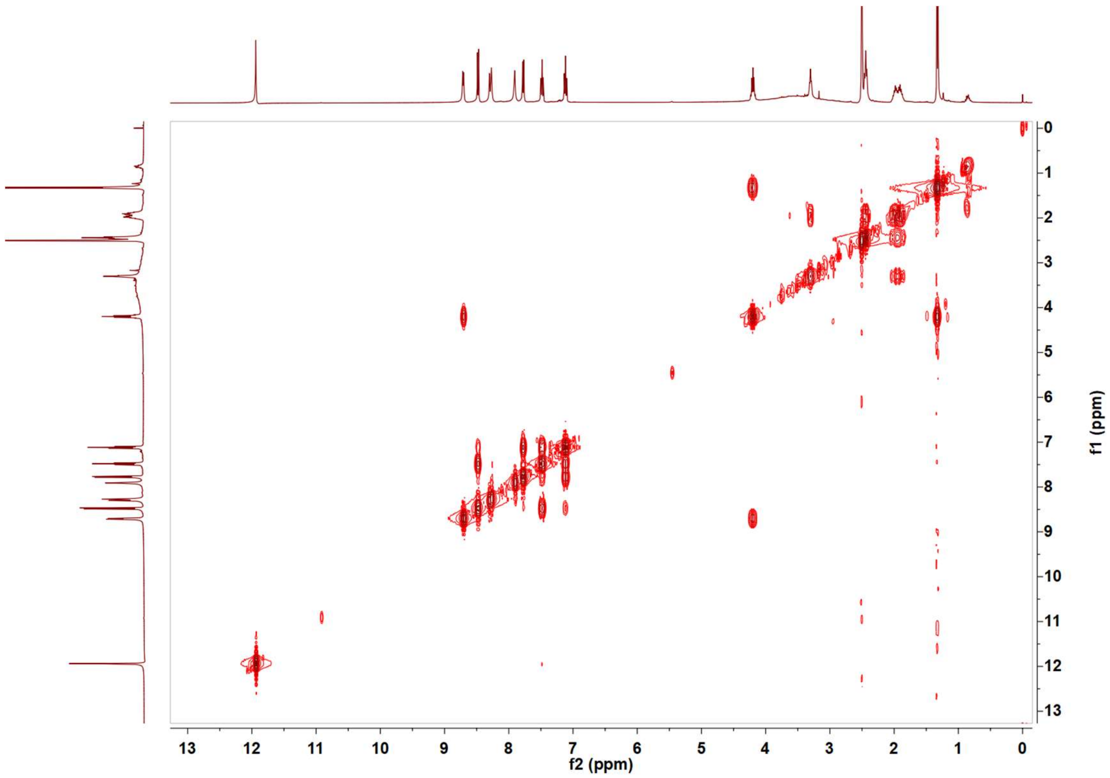

Supplementary Fig. 80 ^{1}H-^{1}H COSY spectrum of compound **11** in DMSO-d_6.

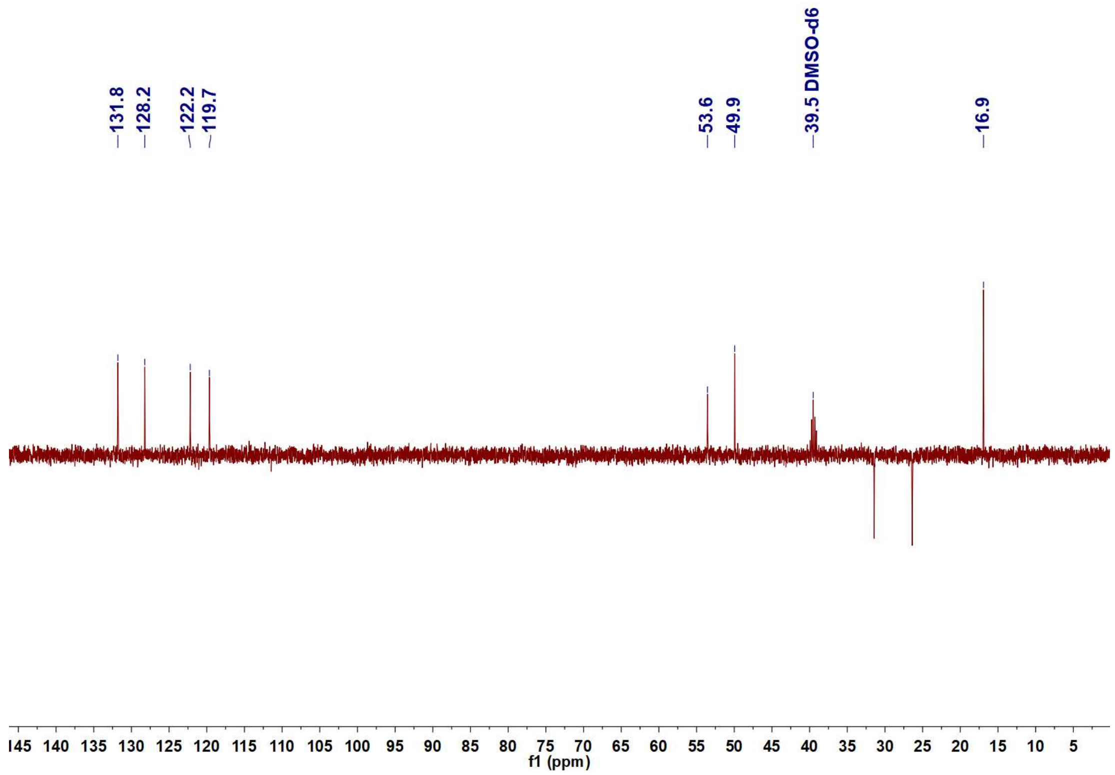

Supplementary Fig. 81 DEPT-135° spectrum of compound **11** in DMSO-d_6.

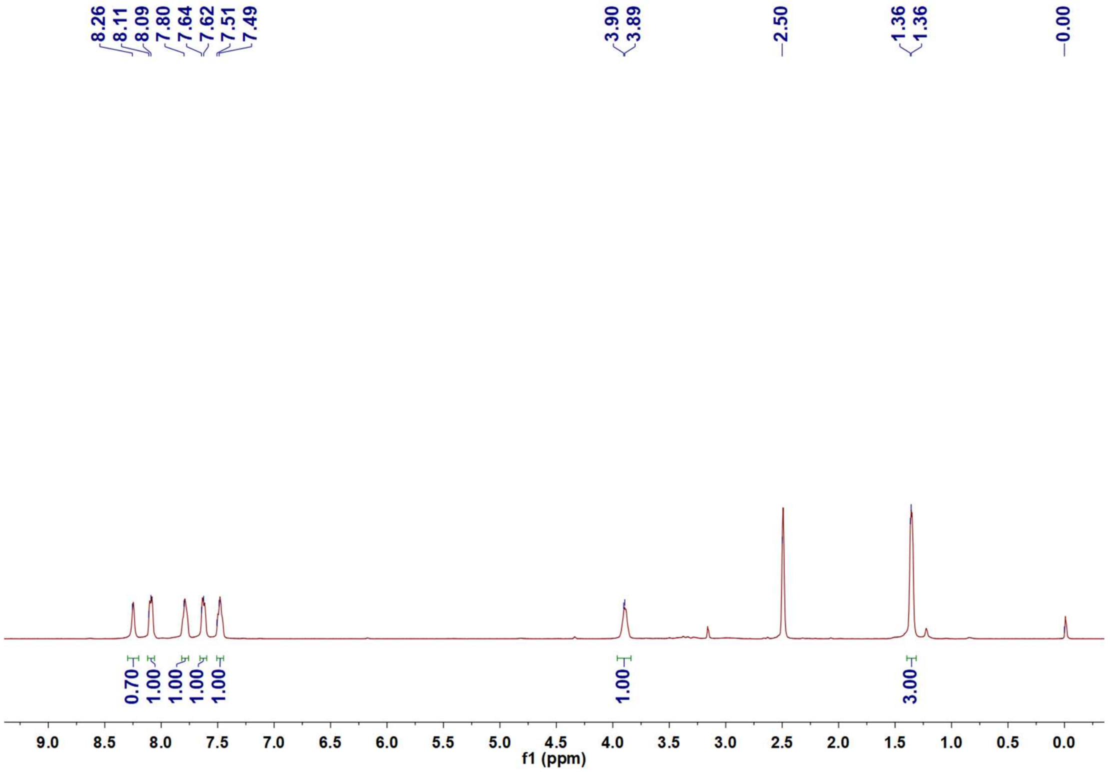

Supplementary Fig. 82 ^{1}H NMR spectrum of compound **14** in DMSO-d_6 (400 MHz).

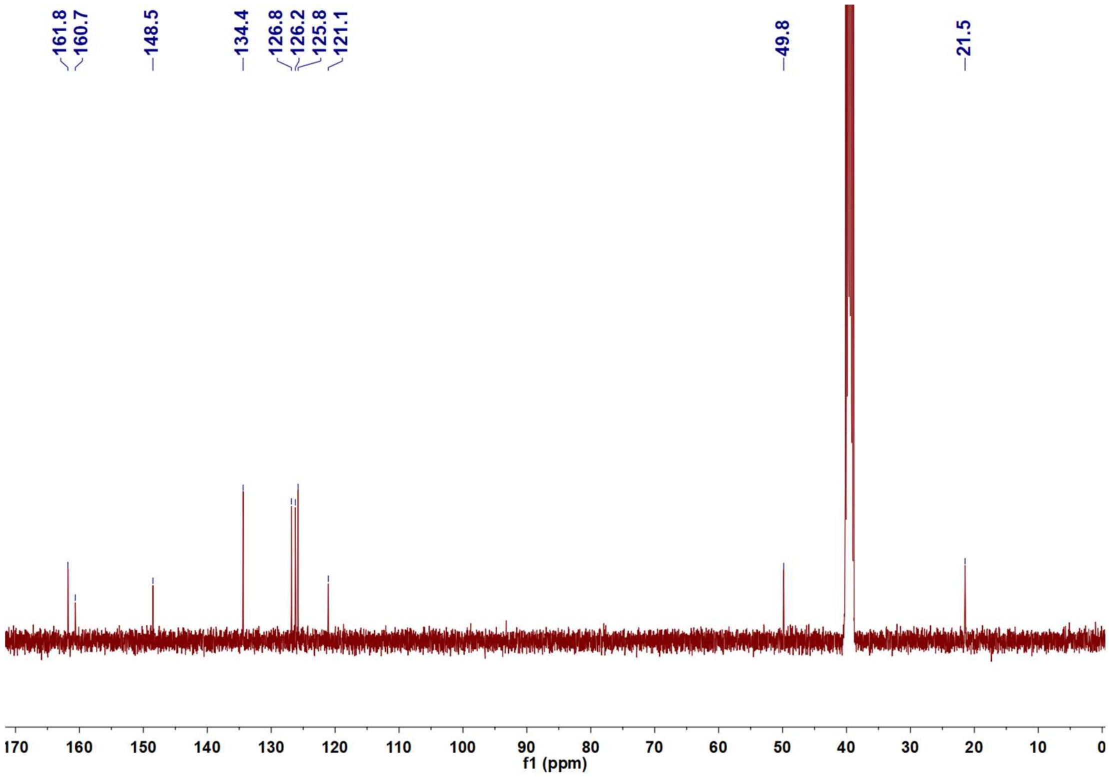

Supplementary Fig. 83 ^{13}C NMR spectrum of compound **14** in DMSO-d_6 (100 MHz).

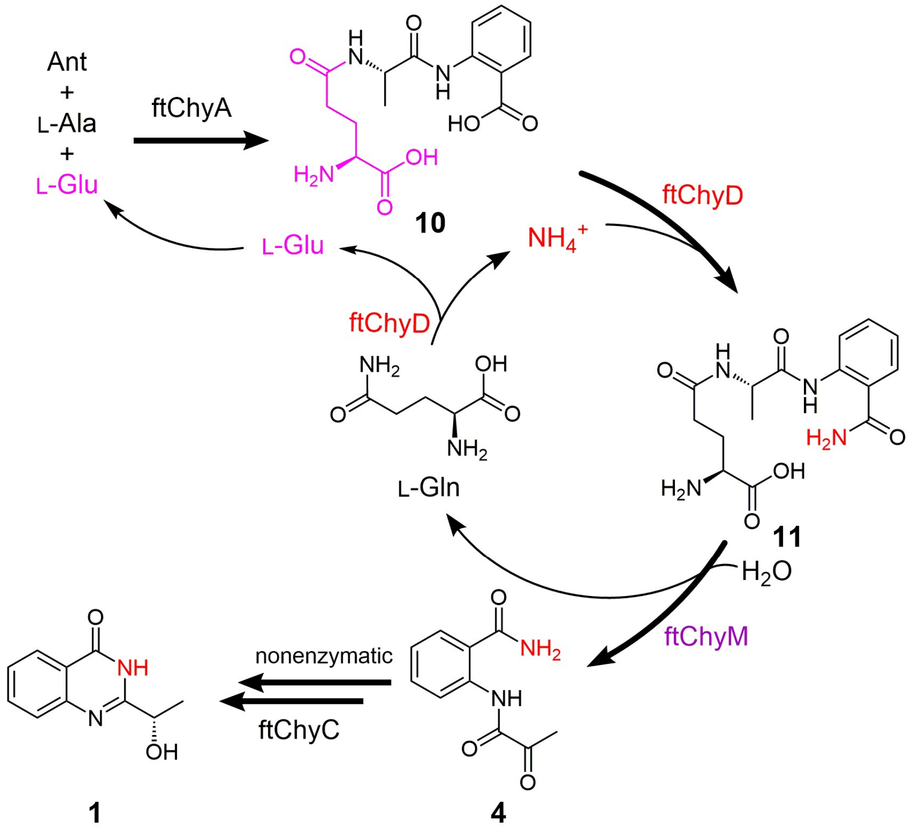

Supplementary Fig. 84 An efficient self-circulation system among ftChyA, ftChyD and ftChyM-catalysed reactions.

4. Supplementary References

1. Viggiano, A. et al. Pathway for the biosynthesis of the pigment chrysogine by *Penicillium chrysogenum*. *Appl. Environ. Microbiol.* **84**, e02246-17 (2018).
2. Zhuo, S. et al. Chemical profile of the secondary metabolites produced by a deep-sea sediment-derived fungus *Penicillium commune* SD-118*. *CHIN. J. OCEANOL. LIMNOL.,* **30***,* 305-314 (2012).
3. Tezuka, Y. et al. Studies on the metabolites of mycoparasitic fungi. I. metabolites of *Cladobotryum varium*. *Chem. Pharm. Bull.* **42**, 2612-2617 (1994).
4. Thi, Q.V. et al. Secondary metabolites from an actinomycete from vietnam's east sea. *Nat. Prod. Commun.* **11**, 401-404 (2016).
5. Bergman J. et al. Synthesis of chrysogine, a metabolite of *Penicillium chrysugenum* and some related 2-substituted 4-(3*H*)-quinazolinones. *Tetrahedron.* **46**, 1295-1310 (1990).
6. Onuki, H., Miyashige, H., Hasegawa, H. & Yamashita, S. NI15501A, a novel anthranilamide derivative from a marine fungus *Penicillium* sp. *J. Antibiot. (Tokyo)*. **51**, 442-444 (1998).
7. Jankowski, F. et al. Efficient microwave-assisted two-step procedure for the synthesis of 1,3-disubstituted-imidazo[1,5-a]quinazolin-5-(4*H*)-ones. *Tetrahedron* **66**, 128-133 (2010).
8. Chen, GD. et al. Benzamides and quinazolines from a mangrove actinomycetes *Streptomyces* sp. (No. 061316) and their inhibiting caspase-3 catalytic activity *in vitro*. *Chem. Pharm. Bull.* (*Tokyo*). **59**, 447-451 (2011).
