# Supplementary material for: Unexpected assembly machinery for 4(3H)-quinazolinone scaffold synthesis
Source: Nat Commun. 2022 Oct 31;13:6522. doi: 10.1038/s41467-022-34340-3 (PMC9622831; doi:10.1038/s41467-022-34340-3)
Supplement: Supplementary file 3 — Reporting Summary [file 41467_2022_34340_MOESM3_ESM.pdf]

nature portfolio

Corresponding author(s): Yi Zou

Last updated by author(s): Oct 21, 2022

Reporting Summary

Nature Portfolio wishes to improve the reproducibility of the work that we publish. This form provides structure for consistency and transparency in reporting. For further information on Nature Portfolio policies, see our Editorial Policies and the Editorial Policy Checklist.

Statistics

For all statistical analyses, confirm that the following items are present in the figure legend, table legend, main text, or Methods section.

n/a	Confirmed	
☐	☒	The exact sample size (*n*) for each experimental group/condition, given as a discrete number and unit of measurement
☐	☒	A statement on whether measurements were taken from distinct samples or whether the same sample was measured repeatedly
☐	☒	The statistical test(s) used AND whether they are one- or two-sided<br>*Only common tests should be described solely by name; describe more complex techniques in the Methods section.*
☒	☐	A description of all covariates tested
☒	☐	A description of any assumptions or corrections, such as tests of normality and adjustment for multiple comparisons
☐	☒	A full description of the statistical parameters including central tendency (e.g. means) or other basic estimates (e.g. regression coefficient) AND variation (e.g. standard deviation) or associated estimates of uncertainty (e.g. confidence intervals)
☐	☒	For null hypothesis testing, the test statistic (e.g. *F*, *t*, *r*) with confidence intervals, effect sizes, degrees of freedom and *P* value noted<br>*Give P values as exact values whenever suitable.*
☒	☐	For Bayesian analysis, information on the choice of priors and Markov chain Monte Carlo settings
☒	☐	For hierarchical and complex designs, identification of the appropriate level for tests and full reporting of outcomes
☒	☐	Estimates of effect sizes (e.g. Cohen's *d*, Pearson's *r*), indicating how they were calculated

Our web collection on statistics for biologists contains articles on many of the points above.

Software and code

Policy information about availability of computer code

Data collection	LC-MS analyses were performed on a Waters ACQUITY H-Class UPLC-MS system coupled to a PDA detector and a SQD2 mass spectrometer (MS) detector with an ESI source. NMR spectra were recorded on a Bruker AVANCE III NMR (400 MHz). HRMS data were obtained on a Fourier transform ion cyclotron resonance-mass spectrometer (FT-ICR-MS) (Bruker SolariII, Bremen, Germany) or quadrupole time-of-flight (QTOF) mass spectrometer (Bruker IMPACT II, Bremen, Germany). MALDI-TOF mass data was recorded by a Bruker MALDITOF mass spectrometer Autoflex Speed.
Data analysis	The softwares used for data analyses are list as follow: NCBI BLAST (https://blast.ncbi.nlm.nih.gov/Blast.cgi), 2nd Find website (http://biosyn.nih.go.jp/2ndFind/), interpro website (http://www.ebi.ac.uk/interpro/search/sequence/), initiative-enzyme similarity tool (EFI-EST) (https://efi.igb.illinois.edu/efi-est/), MEGA7.0 （containing ClustalW), DNAMAN8.0, Cytoscape (v3.8.2), MestReNova12.0.1, GraphPad Prism7.

For manuscripts utilizing custom algorithms or software that are central to the research but not yet described in published literature, software must be made available to editors and reviewers. We strongly encourage code deposition in a community repository (e.g. GitHub). See the Nature Portfolio guidelines for submitting code & software for further information.

Data

Policy information about availability of data

All manuscripts must include a data availability statement. This statement should provide the following information, where applicable:

- Accession codes, unique identifiers, or web links for publicly available datasets
- A description of any restrictions on data availability
- For clinical datasets or third party data, please ensure that the statement adheres to our policy

We declare that all the data generated in this study are available within the main text and the Supplementary Information file. The sequence of ftchy gene cluster is

provided in Source data or under the accession number OP651004 from NCBI: https://www.ncbi.nlm.nih.gov/. NCBI accession codes and hyperlinks for each protein used in this study can be found in Source data. Source data are provided as a Source Data file. Data is also available from the corresponding author upon request.

Field-specific reporting

Please select the one below that is the best fit for your research. If you are not sure, read the appropriate sections before making your selection.

☒ Life sciences ☐ Behavioural & social sciences ☐ Ecological, evolutionary & environmental sciences

For a reference copy of the document with all sections, see nature.com/documents/nr-reporting-summary-flat.pdf

Life sciences study design

All studies must disclose on these points even when the disclosure is negative.

Sample size	No sample size calculation was conducted. In this study, all experiments including both in vitro and in vivo activity assays were performed at three independent times, as they are sufficient for us to assess the presence/absence of a peak or MS signature (representative of a metabolite).
Data exclusions	No data were excluded from the analyses.
Replication	All experiments were repeated independently at least three times and showed similar results. All attempts at replication are successful.
Randomization	Randomization is not relevant to this study because the data in this manuscript mainly include biochemical and structure analyses, and pathway reconstitution. There are no need to allocate samples into experimental groups in essence.
Blinding	All information about the outputs of structure characterized was kept bind for the technicians who performed the NMR. This is a biochemical study and appropriate control experiments were included in vitro and in vivo assays. There are no need to allocate samples into experimental groups in essence.

Reporting for specific materials, systems and methods

We require information from authors about some types of materials, experimental systems and methods used in many studies. Here, indicate whether each material, system or method listed is relevant to your study. If you are not sure if a list item applies to your research, read the appropriate section before selecting a response.

Materials & experimental systems

n/a	Involved in the study	
☒	☐	Antibodies
☒	☐	Eukaryotic cell lines
☒	☐	Palaeontology and archaeology
☒	☐	Animals and other organisms
☒	☐	Human research participants
☒	☐	Clinical data
☒	☐	Dual use research of concern

Methods

n/a	Involved in the study	
☒	☐	ChIP-seq
☒	☐	Flow cytometry
☒	☐	MRI-based neuroimaging
